# A multicentre randomised controlled trial of a guided self-help cognitive behavioural therapy to MANage the impact of hot flushes and night sweats in patients with prostate CANcer undergoing androgen deprivation therapy (MANCAN2)

**DOI:** 10.1186/s13063-023-07325-w

**Published:** 2023-07-10

**Authors:** Simon Crabb, Alannah Morgan, Myra S. Hunter, Evgenia Stefanopoulou, Gareth Griffiths, Alison Richardson, Deborah Fenlon, Louisa Fleure, James Raftery, Cherish Boxall, Sam Wilding, Jacqueline Nuttall, Zina Eminton, Emma Tilt, Alice O’Neill, Roger Bacon, Jonathan Martin

**Affiliations:** 1 Southampton Clinical Trials Unit, University of Southampton, Southampton, UK; 2 University Hospital Southampton NHS Foundation Trust, Southampton, UK; 3 Kings College London, Institute of Psychiatry, Psychology and Neuroscience, Kings College, London, UK; 4 Turning Point - Registered Charity, London, UK; 5 University of Southampton, Southampton, UK; 6 Swansea University, Faculty of Medicine, Health and Life Sciences, Swansea University, Swansea, Wales; 7 Guys and St Thomas NHS Foundation Trust, St Thomas Hospital Westminster Bridge Road, London, UK; 8 Prostate Cancer Support Organisation (PCaSO), Emsworth, UK; 9 Research Department of Primary Care and Population Health, University College London, London, UK

**Keywords:** Prostate cancer, Androgen deprivation therapy, Cognitive behavioural therapy, Hot flushes and night sweats, Cancer nurse specialist, Process evaluation, Normalisation process theory, Randomised control trial, Study within a trial (SWAT), Quality of life

## Abstract

**Background:**

Androgen deprivation therapy (ADT) is prescribed to almost half of all men diagnosed with prostate cancer. Although ADT is effective treatment, with virtually all men with advanced disease showing initial clinical response, it is associated with troublesome side effects including hot flushes and night sweats (HFNS). HFNS can be both frequent and severe and can have a significant impact on quality of life (QoL). They can occasionally be so debilitating that patients stop ADT altogether, despite the increased risk of disease relapse or death. Previous research has found that guided self-help cognitive behavioural therapy (CBT) can be effective in reducing HFNS due to ADT when delivered by a clinical psychologist. MANCAN2 aims test whether we can train the existing NHS Prostate Cancer Nurse Specialist (CNS) team to deliver guided self-help CBT and whether it is effective in reducing the impact of HFNS in men undergoing ADT.

**Methods:**

MANCAN2 is a phase III multicentre randomised controlled trial and process evaluation. Between 144 and 196 men with prostate cancer who are currently receiving ADT and are experiencing problematic HFNS will be individually randomised in a 1:1 ratio in groups of 6–8 participants to either treatment as usual (TAU) or participation in the guided self-help CBT intervention plus TAU. A process evaluation using the normalisation process theory (NPT) framework will be conducted, to understand the CNS team’s experiences of delivering the intervention and to establish the key influencers to its implementation as a routine practice service. Fidelity of implementation of the intervention will be conducted by expert assessment. The cost-effectiveness of the intervention and participant adherence to the trial intervention will also be assessed.

**Discussion:**

MANCAN2 will advance the program of work already conducted in development of management strategies for HFNS. This research will determine whether the severity of ADT-induced HFNS in men with prostate cancer can be reduced by a guided self-help CBT intervention, delivered by the existing NHS prostate cancer CNS team, within a multicentre study. The emphasis on this existing team, if successful, should facilitate translation through to implementation in routine practice.

**Trial registration:**

ISRCTN reference 58720120. Registered 13 December 2022

**Supplementary Information:**

The online version contains supplementary material available at 10.1186/s13063-023-07325-w.

## Background

Prostate cancer is the commonest cancer in UK men, with a rising incidence now reaching around 52,000 per year (https://www.cancerresearchuk.org/health-professional/cancer-statistics/statistics-by-cancer-type/prostate-cancer). Survival rates are improving with 85% now living at least 5 years from diagnosis [1]. It is therefore critical to address the impact of cancer treatment on the increasing numbers who will live with, and beyond, a prostate cancer diagnosis.

Androgen deprivation therapy (ADT) is prescribed to almost half of all men that have been diagnosed with prostate cancer [2]. ADT is a hormonal treatment used to inhibit prostate cancer through the reduction of systemic levels of male androgenic hormones, including testosterone. ADT is used in two main contexts: firstly to reduce relapse rates after a course of radical (curative) radiotherapy when it is typically given as an adjuvant treatment for around 2 years and secondly as palliative life-extending treatment for advanced metastatic disease when it is typically used permanently until the end of life and where the median duration of therapy is 4–6 years [3, 4]. In the UK, ADT is usually administered by a community-based nurse, and through monthly, 3-monthly, or 6-monthly injections of an LHRH analogue. A minority of patients receive LHRH antagonists or undergo surgical castration, as alternative approaches.

Although ADT is an effective treatment, with virtually all men with advanced disease showing an initial clinical response, it is associated with troublesome side effects. Up to 80% of men undergoing ADT suffer from HFNS [5]. Even if discontinued, nearly half of patients experience HFNS for up to 5 years following ADT. HFNS can be both frequent and severe and lead to a significant decrease in quality of life [6]. They may also cause anxiety, low mood and sleep disturbances [7]. HFNS can occasionally be so severe and debilitating that patients become prepared to stop ADT altogether despite the increased risk of disease relapse (for adjuvant treatment) or reduced survival (for palliative treatment) [8].

Current UK practice for management of ADT induced HFNS is rather inconsistent, reflecting the lack of positive and methodologically sound data on which to make recommendations.

Currently, there are few validated safe and effective treatments for men with HFNS [9].

A recent systematic review to determine best practice for managing ADT induced HFNS identified fifteen studies. Eight were of pharmacologic interventions and the remainder for complementary and alternative medicine. It concluded that evidence is insufficient to support even the limited current intervention options [10]. Steroidal progestins (e.g. medroxyprogesterone, cyproterone) showed some benefit at reducing HFNS but were associated with side-effects that were not well tolerated including nausea, weight gain, muscle spasms, depression, insomnia and headaches [11]. Anticonvulsants (gabapentin) and an alpha-agonist antihypertensive (clonidine) did not appear to improve HFNS [10]. Acupuncture demonstrated potential benefit in reducing HFNS and did so without side effects. This was, however, based on data from a handful of small studies limiting interpretation. Acupuncture is not currently routinely available within the NHS for this indication further limiting the practical utility for most patients.

Cognitive behavioural therapy has been found effective in the management of HFNS. Our previous study (MENOS4) in women with breast cancer demonstrated that breast cancer nurse delivered group CBT was both an effective and safe intervention for reducing HFNS symptoms. This multicentre RCT of 130 patients compared group CBT to TAU. The study showed a 46% (6.9–3.7) reduction in the primary endpoint of mean HFNS problem rating score at 26 weeks post randomisation in the CBT arm compared to a 15% (6.5–5.5) reduction in the TAU arm (adjusted mean difference − 1.96, 95% CI − 3.68 to − 0.23, *p* = 0.039) [12]. There were also improvements in secondary outcomes including HFNS frequency, sleep quality, anxiety and depression [12]. Breast cancer nurses from 6 NHS centres were trained to deliver the manualised CBT intervention, delivered in weekly group CBT sessions of 90 min for 6 weeks. Rating for manual adherence, by an independent psychologist from session recordings, showed that a high degree of fidelity was maintained.

We have also adapted our approach to CBT intervention for men undergoing prostate cancer hormonal therapy. MANCAN was a single centre RCT of 68 patients which compared guided self-help CBT to TAU which showed that self-help CBT, delivered by a clinical psychologist, significantly reduced the impact of HFNS at 6 weeks post-randomisation (primary endpoint, adjusted mean difference: − 1.33, 95% CI − 2.07 to − 0.58; *p* = 0.001) [13]. A trend to improvement was maintained at 32 weeks (a secondary endpoint) although at this later time point group differences did not retain statistical significance. There were also significant reductions in negative HFNS beliefs and behaviours, but not in mood or quality of life. A qualitative study with men who participated in the intervention found that the self-help format was acceptable and that the intervention helped them to undertake positive lifestyle changes [14]. We have also developed and validated the HFNS Beliefs and Behaviour Scale for Men assessing men’s beliefs and behaviours in relation to their HFNS [15].

MANCAN2 will advance this program of work by determining whether ADT-induced HFNS in men with prostate cancer can be impacted by a guided self-help CBT intervention (delivered virtually, face to face or hybrid). Critically, this will be undertaken by the existing prostate cancer CNS team within a multicentre study. The emphasis on this existing team, compared to a requirement for clinical psychology involvement, if successful, should facilitate translation through to implementation in routine practice.

## Methods/design

MANCAN2 is a phase III multicentre, randomised controlled trial and process evaluation of guided self-help cognitive behavioural therapy to manage the impact of HFNS symptoms in patients with prostate cancer undergoing androgen deprivation therapy.

### Objectives

The primary objective is to determine whether the addition to treatment as usual (TAU) of virtual self-help CBT, delivered by a patient’s existing prostate cancer CNS team, reduces the impact of HFNS at 6 months post-randomisation in men with prostate cancer undergoing ADT. Secondary objectives will determine the effect of the intervention on the severity of HFNS at 6 weeks post-randomisation and, additionally, the effect of the intervention on HFNS frequency; HFNS beliefs and behaviours and QoL. Other symptoms including anxiety, depression, mood and sleep and men’s compliance with ADT will be examined.

A process evaluation will run parallel to the RCT. The purpose of the process evaluation is to explore the possible barriers and facilitators to implementing the intervention into routine practice, the prostate cancer CNS team’s experiences of introducing the intervention as a new treatment and participant acceptability of the intervention.

The health economics of the intervention, the fidelity of the intervention delivery by the CNS team and the participant adherence to the trial intervention will be examined also.

### Study design

MANCAN2 is a mixed-method (RCT and process evaluation), multicentre, individually randomised controlled trial of a prostate cancer CNS team delivering a 4-week self-help CBT intervention with virtual pre- and post-intervention group workshops plus TAU versus TAU alone.

### Intervention

#### Treatment as usual

All patients, regardless of randomised allocation, will receive TAU for HFNS symptoms as determined by their local care team and institutional practice. TAU will be defined as care consistent with NICE guideline NG131, Prostate cancer: diagnosis and management (https://www.nice.org.uk/guidance/ng131). This includes access to a specialist prostate cancer urologist or oncologist and a named prostate cancer CNS team member, as well as locally and nationally available cancer information. It also allows for options for pharmacotherapy for HFNS which may include, but are not restricted to, those recommended in NG131. At the end of the study, all participants that are randomised into the TAU alone arm will be offered a copy of the CBT Booklet and CD/Audio files (demonstrating the breathing and relaxation exercises).

#### Treatment as usual and guided self-help CBT

The MANCAN2 intervention consists of a 4-week self-help CBT intervention with virtual, face-to-face or hybrid (a mix of virtual and face-to-face delivery) pre- and post-intervention workshops (in groups of 6 to 8 men) delivered by the prostate cancer CNS team in addition to TAU. The intervention for this trial was developed and written by the trial management group (TMG): professor Myra Hunter and Dr. Evgenia Stefanopoulou, with contributions from professor Simon Crabb and Mr. Roger Bacon. The intervention content comprises an instructional booklet (electronic and paper copies available) and guided paced breathing and relaxation exercises audio (downloadable MP3/and CD). The approach is psycho-educational with individual treatment goals and an active focus upon cognitive and behavioural changes. The treatment includes:

- Information about causes of and factors affecting HF/NS
- Monitoring and modifying precipitants, e.g. spicy food, alcohol
- Relaxation and paced breathing, to reduce stress and apply at onset of HF/NS
- Cognitive therapy for unhelpful thoughts and beliefs about HF/NS
- Behavioural strategies to reduce stress and deal with HF/NS in social situations
- Managing sleep and NS, drawing upon CBT for insomnia
- Managing HF/NS and maintaining changes in the context of having prostate cancer.

Participants that do not attend their scheduled group workshop will be offered a short (10-min) 1 to 1 telephone session with an appropriately trained member of the CNS team. The CNS will provide a brief overview of the workshop content. Where CNS teams are unable to offer the 1-to-1 telephone call, or where participants do not respond to the 1-to-1 telephone call, the site research team will send a motivational (missed workshop 1) or maintenance (missed workshop 2) text message to the participant.

#### Process evaluation

In parallel to the RCT, a theory-informed process evaluation will be conducted to evaluate the implementation of the MANCAN2 trial and the possibility of embedding the service as part of routine care thereafter. NPT is a middle-range sociological theory that conceptualises the implementation and integration of innovation in healthcare settings [16]. Recent systematic reviews have supported the use of NPT to explain implementation outcomes [17, 18], supporting the use of NPT as an appropriate conceptual framework.

Semi-structured interviews and a NoMAD (https://normalization-process-theory.northumbria.ac.uk/nomad-study/) questionnaire will be conducted prior to and after site intervention with CNS staff. Up to two staff delivering the intervention at each site will be invited to take part in the interview and questionnaire. The semi-structured interviews will be informed by the four NPT constructs and will include questions to help understand site team attitudes, dynamics, perception of the intervention and influencers that help or hinder its implementation into current work.

Upon approach, staff will be provided with a separate participant information sheet (process evaluation interviews) and an informed consent form, and those with consent (Additional File 1) will take part in remote interview via telephone or video call.

#### Study within a trial (SWAT)

A study within a trial (SWAT) to explore whether a theory-based cover letter will improve the 6-month paper questionnaire return rate will be embedded into the trial. The full SWAT protocol can be found in Additional File 6.

#### Cancer nurse specialist team training

Virtual training of the prostate cancer CNS team to deliver the virtual pre- and post-group intervention workshops will be conducted by a study clinical psychologist. The virtual group training sessions will take place over 2 days, in groups of 10 maximum. The training session will include the manual, the self-help book and specific training about CBT, as well as prostate cancer-specific issues and how to manage groups. The training will provide the background theoretical knowledge and practical skills to facilitate self-help CBT by examining how thinking and behaviour can have a significant impact on men’s experience of HFNS during prostate cancer treatment and through helping men to develop strategies to manage them. These include understanding negative emotions and HFNS, managing unhelpful thoughts and behaviour, improving sleep and using paced breathing to manage flushes and night sweats. Training sessions cover:

- Why CBT is used for HFNS: theory and evidence
- Techniques of stress management, managing hot flushes, sleep and night sweats, maintaining changes and paced breathing

The CNS team will receive ongoing supervision (from the study clinical psychologist) of their delivery of the virtual self-help CBT.

#### Setting

The trial is recruiting from UK NHS sites that host a prostate cancer multi-disciplinary team. To optimise generalisability of results, site selection considerations included ensuring ethnic, geographic and NHS setting diversity.

#### Sample size and recruitment

We regard a ≥ 1.5 point difference in HFNS problem rating as clinically relevant; based on similar interventions, this effect size is also considered realistic [3, 4]. To detect a ≥ 1.5 point difference in mean HFNS problem rating between CBT + TAU versus TAU alone at 6 months post-randomisation, with a standard deviation of 2.21 (32), 90% power and 5% type 1 error rate, requires a sample size of 94. The sample must also account for clustering introduced by the intervention (those attending the same CBT workshop may have more similar outcomes than those outside the group). Assuming 8 participants per group in the intervention, and intra-class correlation (ICC) of 0.01, requires 111 participants. An ICC of 0.01 is thought appropriate given the intervention is largely self-management and smaller ICCs have been observed in similar studies [3]. The sample size increases to 150 (75/arm) allowing for 26% loss to follow-up (as per MENOS4). To recruit 150 patients, a minimum of 6 participating NHS sites were to be involved with flexibility to increase up to 9 sites. Each site was planned to potentially run four groups (2 per randomised arm) consisting of 6 to 8 men, ensuring that a comprehensive process evaluation can be conducted. Across 6 sites this leads to between 144 and 196 participants.

#### Ethical and regulatory aspects

The MANCAN2 trial received full ethical approval from West Midlands-South Birmingham Research Ethics Committee (REC) and the Health Research Authority (HRA) on 16 December 2021 (Additional File 2). The REC reference is 21/WM/0259; the HRA reference is IRAS 304500. Southampton Clinical Trials Unit (SCTU), a Cancer Research UK core funded and UK Clinical Research Collaboration registered Clinical Trials Unit (CTU), is coordinating the trial. University Hospital Southampton NHS Foundation Trust is the sponsor. The trial is funded by the National Institute for Health and Care Research (NIHR) Research for Patient Benefit programme (NIHR201542, Additional File 3). The trial is registered on the UK NIHR trial portfolio (CPMS ID 51149).

#### Study participants

The trial is recruiting men with a diagnosis of prostate cancer, of localised or advanced stage, who are currently receiving ADT and intended to receive a minimum of 6 months of further continuous treatment. Participants may have had potentially curative treatments (including, but not limited to, radiotherapy, brachytherapy, or surgery), with a minimum of 4 weeks between the final fraction/treatment/dose and registration into the trial. Participants must be experiencing problematic HFNS symptoms, defined as a score of 2 or more, on the HFNS rating scale, and willing to attend group workshops (virtual or face-to-face). The full eligibility criteria are listed in Table 1.

**Table 1:** Inclusion and exclusion criteria

MANCAN2 inclusion criteria (items 1–7)	MANCAN2 exclusion criteria (items 8–14)
1. A diagnosis of prostate cancer 2. Localised or advanced disease stage. Patients may have had potentially curative treatments including, but not limited to, radiotherapy, brachytherapy, or surgery 3. Currently receiving ADT and anticipated to require a minimum of 6 months further continuous treatment at the point of registration into the trial. Treatment may have been planned for either a fixed duration (for example, but not limited to, 2 years after radiotherapy) or permanent. Treatment may be with either adjuvant (following potentially curative treatment) or palliative intent. LHRH analogues, LHRH antagonists and surgical castration are all acceptable forms of androgen deprivation. Androgen receptor antagonists, including, but not limited to, bicalutamide, enzalutamide, apalutamide or darolutamide or abiraterone, may be given in combination with androgen deprivation according to local practice 4. Presence of problematic HFNS symptoms defined as a HFNS rating scale score of two or more 5. Ability to read and understand English without assistance 6. 16 years or older 7. Ability to attend virtual (or face-to-face) group workshops through video conferencing software. If this is not feasible, participants must be able to participate in one-to-one workshops by telephone	8. Currently with uncontrolled biochemical, radiological or clinical disease progression or relapse if this would be anticipated to interfere with trial participation as determined by the local principal investigator or co-investigator 9. Currently receiving chemotherapy. Prior chemotherapy must have been completed with a minimum of 4 weeks elapsed between the date of the final dose and confirmation of eligibility. Concomitant use of bone health agents, including zoledronate and denosumab is allowed 10. Currently receiving radical multi-fraction external beam radiotherapy or brachytherapy These must have been completed with a minimum of 4 weeks elapsed between the date of the final fraction/treatment and confirmation of eligibility. Single fraction radiotherapy to sites of painful bony metastatic disease or ‘STAMPEDE style’ palliative prostate radiotherapy is allowed 11. Intention to receive ADT on an intermittent schedule 12. Use of experimental drugs within other interventional clinical trials. Co-recruitment to observational studies, or studies of surgery or focal ablation techniques where the interventional component is complete, is acceptable 13. Currently receiving androgen deprivation as a neoadjuvant treatment 14. Medical or psychiatric conditions or other factors that, in the view of the local PI, are likely to impact on the ability of the patient to participate in the trial procedures and interventions

#### Withdrawal criteria

Participants are free to withdraw their consent from participation at any time. Those withdrawing from the trial intervention only will still be followed-up as per protocol.

### Study procedure

#### Informed consent

Prospective trial participants are approached during conventional or virtual clinic appointments by the site research team. Patients will receive a copy of the MANCAN2 invitation pack including the trial Invite Letter; Screening Questions Form; Patient Contact Details Form; Participant Information Sheet (PIS); Informed Consent Form (ICF) and Baseline Questionnaires (Additional File 4). Interested patients will be asked to return their signed and dated informed consent form to the site research team alongside the additional forms contained in the MANCAN2 Invitation Pack. Optional items on the ICF include participation in process evaluation interviews.

#### Screening

Following the postal return of a signed and dated informed consent form, alongside the screening questions, contact form and baseline questionnaires, the site research team will confirm patient eligibility by completing the eligibility checklist and the clinical baseline form. Screening includes completion of the Hot Flush Rating Scale. Eligibility requires a score 2 or more (on a scale of 1 to 10, where 1 is no problem at all and 10 is very much a problem). Site research teams will screen potentially eligible patients against the inclusion and exclusion criteria (Table 1). A trial screening log captures information such as the date of screening, method of approach and patient ethnicity. Reasons for screen failure are collected for all ineligible patients. Each site team will continue recruitment until a group of 12 to 16 men has formed. A screening review is conducted to verbally reconfirm eligibility and patient interest.

#### Treatment and follow-up visits

Participants are individually randomised (1:1), within groups, to receive TAU plus a guided self-help cognitive behavioural intervention (CBT) or TAU alone. Patients will be randomised by a trial statistician at Southampton Clinical Trials Unit using a pre-generated permuted block method which is stratified by cohort and treatment (curative or palliative). Treatment allocation is unblinded. Sites are informed of the randomisation results via email within 1 day of randomisation, who then contact the patients to discuss study procedures.

Men randomised to the TAU plus guided self-help CBT arm receive a 4-week self-help treatment schedule. The intervention content comprises an instructional self-help booklet (electronic and paper copies available) including information and exercises addressing stress management, paced breathing cognitive and behavioural strategies to improve wellbeing, managing their hot flushes, night sweats and sleep, and a downloadable MP3 audio (and a CD) demonstrating breathing and relaxation exercises. Additionally, participants are invited to participate in two group workshops that are delivered by trained members of the existing prostate CNS team. Sites can deliver workshops in a face-to-face group setting, virtually, or a hybrid approach. The choice of workshop delivery method is at the local CNS team discretion. Workshop 1 takes place during week 1 of the intervention (day 0) and offers practical help on how to use the self-help CBT guide (booklet) appropriately and provide participants with an opportunity to meet other men experiencing similar symptoms. Workshop 2 takes place during week 4 of the intervention with focus on practical tips and strategies to help participants maintain their practice in the future.

Day 0 is defined as the date of workshop 1 for all randomised participants in that cohort. Follow-up questionnaires are posted, from SCTU, to all participants for completion at 6 weeks and 6 months. Where questionnaires have not been returned within 10 days, participants are contacted by telephone and primary outcome data may be collected by telephone. A 1:1 randomised study within a trial (SWAT) is included to explore whether a theory-based cover letter improves the 6-month paper questionnaire return rate. Men randomised to the intervention arm complete a patient evaluation questionnaire following workshop 2.

#### Data collection and management

All data will be pseudonymised and participants will be assigned a unique participant ID. Data are collected on paper case report forms (CRF) and subsequently transferred to an electronic data collection tool (Medidata, Rave). SCTU trial management team regularly check data for missing or anomalous values. Data queries are either automatically generated within the eCRF or manually raised with site by the SCTU trial management team.

#### Plans for assessment and collection of outcomes

Primary and secondary outcomes will be collected on paper case report forms, logs, audio recordings and interviews. Tables 2 and 3 detail the primary and secondary outcomes and the collection methods.

**Table 2:** Schedule of observations and procedures (SPIRIT figure)

**Domain/data type**	**CRF/assessment questionnaire**	**Assessor/person completing the form**	**12–16 participants**		**Week**					
			**Screening**	**Baseline and randomise**	**Intervention delivery**				**Follow-up**	
					**1**	**2**	**3**	**4**	**6**	**26**
**Window**									** ± 2 weeks**	** ± 4 weeks**
Reply slip/contact details	Letter of invitation/contact information form	Participant	X							
Eligibility screening	Baseline checklist CRF	Research nurse (RN)	X							
Route of recruitment	Screening log	RN	X							
Reason not interested	Screening log	RN	X							
Re-eligibility screening check	Baseline checklist CRF	RN		X						
Demographics	Baseline CRF	Participant		X						
Clinical information	Baseline CRF	RN/participant	x							
Consent	Consent form	Participant/RN		X						
Intervention allocation	Randomisation CRF	CTU/statistician		X						
Hot flushes and night sweats	- HFNS rating scale (and HFNS Subscale) - HFNS belief and behaviour scale	Participant		X					X	X
Health-related QoL	- EORTC QLQ-C30	Participant		X					X	X
Anxiety, depression and mood	- GAD7 - PHQ9 - WSAS	Participant		X					X	X
Sleep	PSQI (item 6 only)	Participant		X					X	X
Compliance to ADT	ADT compliance questionnaire	Participant		X					X	X
CNS training	CNS training evaluation questionnaire	CNS	X							
Attendance	Attendance log	CNS			X	X	X	X		
Reflections and questions post CBT group workshops Number of length of supervision sessions	Supervision notes	CNS			X	X	X	X		
Fidelity: pre- and post-intervention workshop delivery	Audio recordings of virtual pre/post group workshops	CNS to record all pre/post group workshops			X			X		
Adherence: self-guided CBT Booklet engagement and lifestyle changes	Patient evaluation questionnaire	Participant (intervention arm only)						X		
Fidelity: pre- and post-intervention workshop delivery	Fidelity checklist	Independent person			2 recordings per site randomly allocated					
Resource usage	Participant resource use questionnaire	Participant							X	X
Management of HFNS	Participant resource use questionnaire	Participant							X	X
Clinic costs	Nurse logs	CNS		X	X	X	X	X		
Nurse time	Nurse logs	CNS		X	X	X	X	X		
Training costs	Nurse logs	CNS		X						
QoL	- EORTC QLQ-C30	Participant		X					X	X
Evaluation of intervention	Patient evaluation questionnaire	Participant (intervention arm only)						X		
Experiences of participating in the CBT group	(Optional) Participant interviews	Participant (intervention arm only)/SCTU research team						X		
Implementation and impact on current services	NoMad Questionnaire/CNS interviews	CNS team member (× 2)	X							X
Implementation of program	Managers interviews	Managers/SCTU research team								X
	Medical staff interviews	Medical staff/SCTU research team								X
SAE collection									X	X

**Table 3:** Primary and secondary outcomes

	Objective	Endpoint used to evaluate
Primary:	To determine whether the addition (to treatment as usual (TAU)) of a 4-week self-help CBT intervention with virtual pre- and post-intervention group workshops, delivered by a patient’s existing prostate cancer clinical nurse specialist (CNS) team, reduces the impact of HFNS at 6 months post randomisation in men with prostate cancer undergoing ADT	HFNS rating scale at 6 months compared to baseline
Secondary:	1. The effect of the intervention on the impact of HFNS at 6 weeks post randomisation	HFNS rating scale at 6 weeks compared to baseline
	2. The effect of the intervention on HFNS frequency	A subscale of the HFNS rating scale, at 6 weeks and 6 months, compared to baseline
	3. The effect of the intervention on men’s HFNS beliefs and behaviours	HFNS beliefs and behaviour scale, at 6 weeks and 6 months, compared to baseline
	4. The effect of the intervention on QoL	- EORTC QLQ-C30, at 6 weeks and 6 months, compared to baseline
	5. The effect of the intervention on other symptoms including anxiety, depression, mood and sleep	Anxiety, depression and mood: - Generalised Anxiety Disorder Questionnaire (GAD7), at 6 weeks and 6 months, compared to baseline - Patient Health Questionnaire-9 (PHQ9), at 6 weeks and 6 months, compared to baseline - Work and Social Adjustment Scale (WSAS), at 6 weeks and 6 months, compared to baseline
		Sleep: - Pittsburgh Sleep Quality Index (PSQI, item 6), at 6 weeks and 6 months, compared to baseline
	6. The effect of the intervention on men’s compliance to ADT	Percentage of men compliant with the planned duration of ADT at 6 weeks and 6 months
	7. The level fidelity of CBT when delivered by the prostate cancer CNS team	- Audio recordings of the virtual (pre- and post) intervention group workshops. An independent person will rate a random selection of these for adherence to the treatment manual - Workshop attendance logs (completed by CNS) will measure patient compliance to the virtual pre- and post-group intervention workshops - Post-intervention questionnaire completed by patients will measure how much of the booklet/CD patients engaged with and what lifestyle changes they have made
	8. Resource usage analyses	- CNS team logs to record staff training cost, time to deliver intervention in a virtual capacity - Participant resource use questionnaire
	9. Prostate cancer CNS team experiences of delivering this new service	Interviews with prostate cancer CNS team members
	10. Participants’ acceptability of the intervention	Interviews with participants
	11. Explore barriers and facilitators to implementing the intervention into routine practice	Interviews with CNS team, medic and manager
	12. Health economics of the intervention	- Measurement of quality-adjusted life years (QALY) - Collection of data on expected service use - Collection of data pertaining to the cost of the intervention

#### Oversight and monitoring

The MANCAN2 TMG is responsible for trial progress oversight and is chaired by the chief investigator. The TMG includes representatives with expertise in oncology, nursing, psychology, process evaluation and medical statistics with a patient and public involvement member and SCTU staff involved in the day-to-day management of the trial.

No DMEC will be convened for MANCAN2; this role will be assumed by the trial steering committee. An independent (TSC) for MANCAN2 exists to safeguard the interests of trial participants and to monitor the main outcome measures and overall conduct of the trial on behalf of the sponsor and funder. Monitoring is conducted centrally by the SCTU trial management team.

### Statistical analysis

#### Primary and secondary outcome analysis

The primary outcome measure, HFNS rating scale compared to baseline, will be compared between the CBT intervention + TAU arms at 6 months post randomisation. Difference in HFNS rating scale between the two groups will be analysed using a linear mixed model, adjusting for baseline HFNS rating scale and curative/palliative status. Cohort will be included in the model as a random effect. Interpretation of the effect of the intervention will be based on the regression coefficient for arm and corresponding 95% confidence interval. The difference in HFNS rating scale will also be compared at 6 weeks post randomisation. Secondary outcomes including the frequency of HFNS, the HFNS Beliefs and Behaviour Scale, summary scores of the EORTC QLQ-C30, GAD7, PHQ9, WSAS and PSQI questionnaires at 6 weeks and 6 months post-randomisation will be analysed similarly, accounting for stratification factors and clustering.

Analyses will be based on a modified intention-to-treat sample (i.e. excluding participants not contributing data). This will be supported by an analysis that deals with missing data should missingness lead to > 10% of the sample being excluded. Methods may involve either multiple imputation using chained equations or full information maximum likelihood. MANCAN2 will be analysed according to the principles of the International Conference on Harmonisation E9 guidelines and reported using Consolidated Standards of Reporting Trials (CONSORT). A full statistical analysis plan (SAP) will be developed prior to the final analysis.

#### Health economic analysis

The economic analysis will take an NHS perspective to estimate both cost effectiveness (£ per change in HFRS) and cost utility (Incremental £/QALY) as well as a budget impact. QALYs will be estimated from EORTC using the SCHARR algorithm [19]).

Costs will include that of the intervention and on any changes in use of NHS services. As the intervention cost might differ between that in the trial and in routine practice, we will take two approaches, one detailing changes in CNS team total time allocations in each arm during the trial and another collecting data on views of both the CNS team and managers on the extent to which the intervention could be incorporated into standard practice (and under what circumstances such as being included in training and in guidelines). While both these costs will be reported, the latter would be more relevant in estimating budget impact if implemented in routine practice. The cost of changes in service use will be based on a customised resource use patient questionnaire at 6 weeks and 6 months.

Estimates of both cost and QALY increments will be estimated for each patient and incorporated into the planned statistical analyses described above. If a clinically relevant gain in the primary outcome is found to be statistically significant, more detailed economic modelling will be carried out, to explore uncertainty and to extend the time frame beyond that of the trial. This will build on the recent cost effectiveness modelling for similar interventions for hot flushes in breast cancer [20].

All relevant resource items identified will be costed using published national cost data (British National Formulary and Personal Social Services Research Unit, and NHS reference cost). Accumulated costs and QALYs per patient will be estimated by means of area under the curve. Where appropriate, we will estimate incremental cost-effectiveness ratios (ICERs). We will estimate mean values and 95% percentiles using nonparametric bootstrapping. We will produce cost-effectiveness acceptability curves (CEACs) to illustrate the uncertainty of such estimates. Major assumptions made in the costing and QALYs will be tested by means of sensitivity analyses.

#### Qualitative analysis of process evaluation

Interviews by video conferencing software or telephone will be conducted with participants and key stakeholders from each centre at completion of the intervention, to determine acceptability and factors that might influence routine practice adoption. Interview recordings will be transcribed and identifying information will be anonymised.

A two-stage approach to analysis will take place in parallel from completion of the first interview. Initially, transcripts will be coded using inductive thematic analysis using the constant comparative method and, subsequently, map emergent themes onto the normalisation process theory (NPT) framework. This will be done to test the robustness of the NPT constructs against emergent themes and to facilitate a clear and thorough data-driven analysis. Analysis will be an iterative process between coding, emergent themes and NPT mapping and will involve interrogating the data for disconfirming evidence that does not fit inside the NPT framework, increasing the rigour and validity of the analysis process. Members of the research team will hold coding and analysis meetings to discuss coding strategy, proposed themes and NPT mapping and subsequently tested in the data. The NVivo 10 software program will be used to facilitate data storage, categorisation and retrieval.

#### Measure of intervention fidelity (CNS workshop delivery)

Fidelity will be measured by recording all pre- and post-intervention group workshops (with consent obtained from participants), and 20% will be randomly selected (with a computer-generated random number sequence), ensuring two sessions per site. An independent person will rate these for adherence to the treatment manual. Some recordings may be used by the trial team to provide feedback to CNS team members to ensure adherence to the treatment model.

#### Measure of intervention adherence

Adherence to self-help CBT will be measured by the number of booklet chapters read and the number of times a participant reports practising relaxation and paced breathing weekly.

### Adverse event reporting and harms

Data on adverse events will be collected at 6 weeks and 6 months post intervention. The PI will assign the seriousness, causality and expectedness of events. SCTU will notify the REC of all related and unexpected serious adverse events occurring during the study within 15 days of report receipt.

### End of trial

The end of the trial will occur following the collection of data for the 6-month assessment point of the last cohort of participants randomised to the trial.

## Discussion

MANCAN2 will advance the programme of work already conducted in the development of management strategies for HFNS, by determining whether ADT-induced HFNS in men with prostate cancer can be alleviated by a guided self-help CBT intervention, with pre- and post-intervention workshops delivered by the existing prostate cancer CNS team within a multicentre study. The emphasis on this existing team, if successful, should facilitate translation through to implementation in routine practice.

## Trial status

MANCAN2 has opened nine sites to recruitment between 04 March 2022 and 29 August 2022. The current protocol is version 3, dated 27 July 2022.

REC/HRA-approved protocol amendments will be communicated to sites via email and updated trial documentation provided centrally via the trial website. Trial registries will be amended where relevant with explanations for these changes.

Approximate end date for recruitment: 31 March 2023.

## Supplementary Information

**Additional file 1. **A copy of the Process Evaluation Participant Information Sheet (Medic, Manager, CNS) and Informed Consent Form.

**Additional file 2. **REC Favourable Opinion.

**Additional file 3. **Confirmation of NIHR Funding.

**Additional file 4. **Patient Invitation Pack (including Patient Information Sheet and Informed Consent Form.

**Additional file 5. **(The SPIRIT Checklist). Standard Protocol Items: Recommendations for Interventional Trials (SPIRIT): a checklist for a set of scientific, ethical and administrative elements recommended to be listed in a protocol [21] .

**Additional file 6. **Full Protocol

## Data Availability

Pseudonymised individual participant data within the clinical trial dataset will be available for sharing via controlled access by authorised SCTU staff (as delegated to SCTU by the trial sponsor). Data access can be requested via a SCTU Data Release application form, detailing the specific requirements and the proposed research, statistical analysis, publication plan and evidence of research group qualifications. Please email the completed form to the SCTU Data Release Committee Coordinator at ctu@soton.ac.uk. Data access requests are reviewed against specific eligibility criteria by the SCTU data custodian and key members of the trial team, including a statistician and chief investigator or by an external independent review panel. Decisions about requests are made promptly and usually no more than 3 months after receipt of request. Responses to all data requests, with a clear rationale for any refusals, will be sent promptly to the data requester.

## Abbreviations

ADT: Androgen deprivation therapy
HFNS: Hot flushes and night sweats
CBT: Cognitive behavioural therapy
TAU: Treatment as usual
RCT: Randomised controlled trial
AE: Adverse event
eCRF: Electronic case report form
CRF: Case report form
ICF: Informed consent form
TMG: Trial management group
TSC: Trial steering committee
SCTU: Southampton Clinical Trials Unit
