# Supplementary material for: A multicentre randomised controlled trial of a guided self-help cognitive behavioural therapy to MANage the impact of hot flushes and night sweats in patients with prostate CANcer undergoing androgen deprivation therapy (MANCAN2)
Source: Trials. 2023 Jul 10;24:450. doi: 10.1186/s13063-023-07325-w (PMC10332063; doi:10.1186/s13063-023-07325-w)
Supplement: Supplementary file 1 — Additional file 1. A copy of the Process Evaluation Participant Information Sheet (Medic, Manager, CNS) and Informed Consent Form. [file 13063_2023_7325_MOESM1_ESM.pdf]

MANCAN2 PROCESS EVALUATION: PARTICIPANT INFORMATION SHEET

<u>MANAGER</u>

**MANCAN2 (MANaging symptoms during prostate CANcer treatment):**<br>A multicentre randomised controlled trial (RCT) of a virtual self-help cognitive behavioural therapy (CBT) intervention to reduce the impact of hot flush and night sweat (HFNS) symptoms in men with prostate cancer undergoing androgen deprivation therapy (ADT)

YOUR PERCEPTIONS OF THE MANCAN2 STUDY

You are invited to take part in the MANCAN2 Process Evaluation. If you wish to participate, we will ask for you to take part in an interview with a member of the Southampton Clinical Trials Unit (SCTU) research team. The purpose of this interview is to understand your perceptions of the study intervention and to establish whether the intervention has potential to become a routine practice service.

What is the purpose of the MANCAN2 Study?

Half of all men that are diagnosed with prostate cancer receive treatment to reduce or block their levels of the male hormone, testosterone (called androgen deprivation therapy or ADT).

Although ADT is an effective treatment, it is associated with troublesome side effects that commonly include Hot Flushes and Night Sweats (HFNS). In fact, up to 80% of men undergoing ADT suffer from these.

HFNS can be both frequent and severe and can lead to a significant decrease in quality of life. They may also cause anxiety, low mood and sleep disturbances. Sometimes, they can be so severe and debilitating, that some men decide to stop treatment altogether.

Previous research has found that guided self-help Cognitive Behavioural Therapy (CBT) can be effective in reducing HFNS in men, when delivered by a clinical psychologist. We now want to find out if we can train NHS Cancer Nurse Specialists to deliver virtual guided self-help CBT and whether it is effective in reducing the impact of HFNS in men undergoing ADT.

MANCAN2 will test whether a 4-week guided self-help CBT intervention with pre and post group workshops (delivered virtually by the Cancer Nurse Specialist team), alongside the care that prostate patients would normally receive from their doctor, reduces the impact of HFNS more than normal care alone

For further details about the MANCAN2 trial, please refer to the enclosed patient participant information sheet.

What is the purpose of the questionnaires and interviews?

If MANCAN2 is found to be effective, there is potential to incorporate this intervention into currently existing staffing structures designed to support men living with and beyond cancer. Specifically, the guided self-help CBT could be delivered to patients (by their CNS team) as part of their usual treatment programme.

It is therefore important for us to understand site team attitudes, dynamics, perception of the intervention and ability to integrate it into current work.

Why have I been invited to take part?

You have been invited to take part because the <site name> MANCAN2 Principal Investigator <name> has identified you as a suitable person to approach for the interview.

Please note: *In order to be suitable you must be a member of management who has influence on the implementation of a new service within the NHS.*

Do I have to take part?

No, you are free to decide whether or not to take part. If you decide to take part, you are still free to stop at any time without giving a reason. No questions will be asked if you stop. Furthermore, during the interview if there is a question you prefer not to answer, that question can be left out.

What will happen if I take part?

If you decide that you would like to take part, the first step is for you to complete the enclosed Informed Consent Form and Contact Details Form. Once you have completed, please send your completed forms to the research team at Southampton Clinical Trials Unit (SCTU) via email, using the secure University of Southampton safesend (instructions on how to send using safesend can be found on the Informed Consent Form).

Once you have returned your informed consent form and contacts form to us, a member of the SCTU research team will contact you.

You will be invited to participate in an interview. The interview will take place after the first group of patients has completed the intervention, prior to recruitment end at site. The interview will explore:

1. The process of encouraging CBT take-up in patients
2. How patients access the intervention
3. The potential for running the intervention in routine practice
4. If the intervention fits with organisational priorities.

The SCTU research team will arrange a convenient time with you to conduct a telephone (or video conference) interview lasting between 45 minutes and 1 hour. Interviews will be recorded.

What will happen after the interview?

The interview recording will be labelled with a code number and will be transferred securely to university approved transcriber who will type out everything you said in the interview. The transcriber signs an agreement to keep everything you say in the interview confidential. We will remove any names, which would identify you or any other individuals.

The digital recording and the typed- up record (transcript), identified only by the code number, will be kept in a secure place with the University of Southampton.

What will happen to the results of the study?

At the end of the study we will submit our results to be published in medical journals. We may use some anonymised quotes from the information you gave us, but we will never include anything that would allow someone to identify you.

A summary of our research findings will be sent to everyone who requests to receive these. There is a section on the informed consent form where you can opt to receive the results.

Can I withdraw?

Yes. You have the right to change your mind and withdraw at any time without giving a reason and without your participant rights being affected. You can request for any data collected to be deleted.

However, it may not be possible for you to withdraw your data once the analysis has started because the data collected will already be pseudonymised and have been used but you can inform the research team if you do not want your anonymised interview content to be used in publications.

Are there any risks involved?

No, but you will be giving up some of your time to give the interview. You have the right not to answer any question and to ask for a break if you wish.

Will my participation and the information that I provide be kept confidential?

Yes. The interview recordings will be typed up and your name or any other details that can identify you will be removed, to ensure your privacy.

The recordings will be kept securely on the computer system at the University of Southampton, which only the research team can access. An audio recording of the interview will be sent to a University approved transcription company who will securely manage the audio recording and return a transcribed word document via email, where a copy will be stored on the secure network for analysis.

These anonymised transcripts (which don't include information which identifies who you are) will be stored on a password protected file on secure University of Southampton computers for up to 3 years. After this time, they will be destroyed. Interview recordings will be destroyed at the end of the study.

The findings from this study will be used in research report publications but no names will be included so any quotes used from the interview will be anonymous. We will remove any names, which would identify you or any other individuals.

Your contact details will be held separately from your interview information in a secure password-protected file on a University computer, we will use these details only to arrange the interview. These details will be deleted after your interview.

Data Protection Privacy Notice

See *Addendum 1* for information regarding the University of Southampton's Data Protection Privacy Notice.

What if there is a problem?

If you wish to complain, or have any concerns about any aspect of this study please contact the study Trial Manager by email (mancan2@soton.ac.uk) or telephone (023 8120 5589).

Who is organising and funding the research?

The study is being funded by the National Institute for Health Research (NIHR) Research for Patient Benefit (RfPB) programme. The study Sponsor is the University Hospital Southampton NHS Foundation Trust. The University of Southampton Clinical Trials Unit (SCTU) is responsible for oversight and management of the study.

Who has reviewed the study?

This study has been reviewed and approved by the <ethics committee> on <dd/mmm/yyyy> and the REC reference number is <insert>.

How can I find out more?

If you have any questions at this stage and would like to discuss them with the SCTU research team please call:

MANCAN2 Trial Manager: mancan2@soton.ac.uk. Tel: 023 8120 5589

Addendum 1: Data Protection Privacy Notice

The University Hospital Southampton NHS Foundation Trust are the Sponsor for this study, and retain legal responsibility for the study. The Sponsor have delegated the responsibility of overall study coordination and data controller to the Southampton Clinical Trials Unit (SCTU).

SCTU are governed by the University of Southampton. The University of Southampton conducts research to the highest standards of research integrity. As a publicly-funded organisation, the University has to ensure that it is in the public interest when we use personally-identifiable information about people who have agreed to take part in research. This means that when you agree to take part in a research study, we will use information about you in the ways needed, and for the purposes specified, to conduct and complete the research project. Under data protection law, 'Personal data' means any information that relates to and is capable of identifying a living individual. The University's data protection policy governing the use of personal data by the University can be found on its website (https://www.southampton.ac.uk/legalservices/what-we-do/data-protection-and-foi.page).

This Participant Information Sheet tells you what data will be collected for this study and whether this includes any personal data. Please ask the research team if you have any questions or are unclear what data is being collected about you.

Our privacy notice for research participants provides more information on how the University of Southampton collects and uses your personal data when you take part in one of our research projects and can be found at:
http://www.southampton.ac.uk/assets/sharepoint/intranet/ls/Public/Research%20and%20Integrity%20Privacy%20Notice/Privacy%20Notice%20for%20Research%20Participants.pdf
Any personal data we collect in this study will be used only for the purposes of carrying out our research and will be handled according to the University's policies in line with data protection law. If any personal data is used from which you can be identified directly, it will not be disclosed to anyone else without your consent unless the University of Southampton is required by law to disclose it.

Data protection law requires us to have a valid legal reason ('lawful basis') to process and use your Personal data. The lawful basis for processing personal information in this research study is for the performance of a task carried out in the public interest. Personal data collected for research will not be used for any other purpose.

For the purposes of data protection law, the University of Southampton is the 'Data Controller' for this study, which means that we are responsible for looking after your information and using it properly. The University of Southampton will keep non- identifiable information about you for 25 years after the study has finished after which time any link between you and your information will be removed.

To safeguard your rights, we will use the minimum personal data necessary to achieve our research study objectives. Your data protection rights – such as to access, change, or transfer such information - may be limited, however, in order for the research output to be reliable and accurate. The University will not do anything with your personal data that you would not reasonably expect.
If you have any questions about how your personal data is used, or wish to exercise any of your rights, please consult the University's data protection webpage:
(https://www.southampton.ac.uk/legalservices/what-we-do/data-protection-and-foi.page) where you can make a request using our online form. If you need further assistance, please contact the University's Data Protection Officer (data.protection@soton.ac.uk).

MANCAN2 PROCESS EVALUATION: PARTICIPANT INFORMATION SHEET

PROSTATE CANCER NURSE SPECIALIST TEAM MEMBERS

MANCAN2

MANCAN2 (MANaging symptoms during prostate CANcer treatment):
A multicentre randomised controlled trial (RCT) of a virtual self-help cognitive behavioural therapy (CBT) intervention to reduce the impact of hot flush and night sweat (HFNS) symptoms in men with prostate cancer undergoing androgen deprivation therapy (ADT)

YOUR EXPERIENCE OF THE MANCAN2 STUDY

You are invited to take part in the MANCAN2 Process Evaluation. If you wish to participate, we will ask for you to complete two questionnaires and to take part in two interviews with a member of the Southampton Clinical Trials Unit (SCTU) research team, to share your experiences of introducing and running the MANCAN2 intervention.

What is the purpose of the MANCAN2 Study?

Half of all men that are diagnosed with prostate cancer receive treatment to reduce or block their levels of the male hormone, testosterone (called androgen deprivation therapy or ADT).

Although ADT is an effective treatment, it is associated with troublesome side effects that commonly include Hot Flushes and Night Sweats (HFNS). In fact, up to 80% of men undergoing ADT suffer from these.

HFNS can be both frequent and severe and can lead to a significant decrease in quality of life. They may also cause anxiety, low mood and sleep disturbances. Sometimes, they can be so severe and debilitating, that some men decide to stop treatment altogether.

Previous research has found that guided self-help Cognitive Behavioural Therapy (CBT) can be effective in reducing HFNS in men, when delivered by a clinical psychologist. We now want to find out if we can train NHS Cancer Nurse Specialists to deliver virtual guided self-help CBT and whether it is effective in reducing the impact of HFNS in men undergoing ADT.

MANCAN2 will test whether a 4-week guided self-help CBT intervention with pre and post group workshops (delivered virtually by the Cancer Nurse Specialist team), alongside the care that prostate patients would normally receive from their doctor, reduces the impact of HFNS more than normal care alone.

For further details about the MANCAN2 trial, please refer to the enclosed patient participant information sheet.

What is the purpose of the MANCAN2 Process Evaluation Questionnaires and Interviews?

If MANCAN2 is found to be effective, there is potential to incorporate this intervention into currently existing staffing structures designed to support men living with and beyond cancer. Specifically, the guided self-help CBT could be delivered to patients (by their CNS team) as part of their usual treatment programme.

It is therefore important for us to understand site team attitudes, dynamics, perception of the intervention and ability to integrate the new programme into current work.

Why have I been invited to take part?

You have been invited to take part because you will be delivering the MANCAN2 pre and post intervention group workshops.

Do I have to take part?

No, you are free to decide whether or not to take part. If you decide to take part, you are still free to stop at any time without giving a reason. No questions will be asked if you stop. Furthermore, during the interview if there is a question you prefer not to answer, that question can be left out.

If you decide not to take part, you can still participate in the delivery of the study.

What will happen if I take part?

If you decide that you would like to take part, the first step is for you to complete the enclosed Informed Consent Form and Contact Details Form. Once you have completed, please send your completed forms to the research team at Southampton Clinical Trials Unit (SCTU) via email, using the secure University of Southampton safesend (instructions on how to send using safesend can be found on the Informed Consent Form).

Once you have returned your informed consent form and contacts form to us, a member of the SCTU research team will contact you. You will be invited to participate in two interviews and to complete some questionnaires:

1. **Pre- Intervention Interview + Questionnaires**
   The pre-intervention interview and questionnaire completion will be scheduled to take place at a suitable time before you have received your virtual group workshop delivery training from the study clinical psychologist.

   The pre-intervention interview will help us to understand the organisation, site and working group dynamics, current role and practice for treating HFNS in men, how patients will gain access and be recruited to MANCAN2 and will explore your initial perceptions of the trial.

2. Post- Intervention Interview + Questionnaires

The post-intervention interview and questionnaire completion will be scheduled to take place once (at least) one group of participants have completed the post-intervention workshop at site.

The post-intervention interview will help us to understand the experience and work taken to implement the intervention. This will include perceptions of the self-help CBT training, new understandings of HFNS treatment, experiences delivering the intervention, any impact on current work and the team-working process.

The SCTU research team will arrange a convenient time with you to conduct a telephone (or video conference) interview lasting between 45 minutes and 1 hour. Interviews will be recorded. The research team will also send to you a questionnaire to for you to complete electronically and you will be asked to return your completed questionnaire using a secure email (nhs.net or University of Southampton safesend) account. The questionnaires will take approximately 10 minutes to complete.

What will happen after the interview?

The interview recording will be labelled with a code number and will be transferred securely to university approved transcriber who will type out everything you said in the interview. The transcriber signs an agreement to keep everything you say in the interview confidential. We will remove any names, which would identify you or any other individuals.

The digital recording and the typed-up record (transcript), identified only by the code number, will be kept in a secure place with the University of Southampton.

What will happen to the results of the study?

At the end of the study we will submit our results to be published in medical journals. We may use some anonymised quotes from the information you gave us, but we will never include anything that would allow someone to identify you.

A summary of our research findings will be sent to everyone who requests to receive these. There is a section on the informed consent form where you can opt to receive the results.

Can I withdraw?

Yes. You have the right to change your mind and withdraw at any time without giving a reason and without your participant rights being affected. You can request for any data collected to be deleted.

However, it may not be possible for you to withdraw your data once the analysis has started because the data collected will already be pseudonymised and have been used but you can inform the research team if you do not want your anonymised interview content to be used in publications.

Are there any risks involved?

No, but you will be giving up some of your time to give the interview. You have the right not to answer any question and to ask for a break if you wish.

Will my participation and the information that I provide be kept confidential?

Yes. The interview recordings will be typed up and your name or any other details that can identify you will be removed, to ensure your privacy.

The recordings will be kept securely on the computer system at the University of Southampton, which only the research team can access. An audio recording of the interview will be sent to a University approved transcription company who will securely manage the audio recording and return a transcribed word document via email, where a copy will be stored on the secure network for analysis.

These anonymised transcripts (which don't include information which identifies who you are) will be stored on a password protected file on secure University of Southampton computers for up to 3 years. After this time, they will be destroyed. Interview recordings will be destroyed at the end of the study.

The findings from this study will be used in research report publications but no names will be included so any quotes used from the interview will be anonymous. We will remove any names, which would identify you or any other individuals.

Your contact details will be held separately from your interview information in a secure password-protected file on a University computer, we will use these details only to arrange the interview. These details will be deleted after your interview.

Data Protection Privacy Notice

See *Addendum 1* for information regarding the University of Southampton's Data Protection Privacy Notice.

What if there is a problem?

If you wish to complain, or have any concerns about any aspect of this study please contact the study Trial Manager by email (mancan2@soton.ac.uk) or telephone (023 8120 5589).

Who is organising and funding the research?

The study is being funded by the National Institute for Health Research (NIHR) Research for Patient Benefit (RfPB) programme. The study Sponsor is the University Hospital Southampton NHS Foundation Trust. The University of Southampton Clinical Trials Unit (SCTU) is responsible for oversight and management of the study.

Who has reviewed the study?

This study has been reviewed and approved by the <ethics committee> on <dd/mmm/yyyy> and the REC reference number is <insert>.

How can I find out more?

If you have any questions at this stage and would like to discuss them with the SCTU research team, please call:

MANCAN2 Trial Manager: mancan2@soton.ac.uk. Tel: 023 8120 5589

Addendum 1: Data Protection Privacy Notice

The University Hospital Southampton NHS Foundation Trust are the Sponsor for this study, and retain legal responsibility for the study. The Sponsor have delegated the responsibility of overall study coordination and data controller to the Southampton Clinical Trials Unit (SCTU).

SCTU are governed by the University of Southampton. The University of Southampton conducts research to the highest standards of research integrity. As a publicly-funded organisation, the University has to ensure that it is in the public interest when we use personally-identifiable information about people who have agreed to take part in research. This means that when you agree to take part in a research study, we will use information about you in the ways needed, and for the purposes specified, to conduct and complete the research project. Under data protection law, 'Personal data' means any information that relates to and is capable of identifying a living individual. The University's data protection policy governing the use of personal data by the University can be found on its website (https://www.southampton.ac.uk/legalservices/what-we-do/data-protection-and-foi.page).

This Participant Information Sheet tells you what data will be collected for this study and whether this includes any personal data. Please ask the research team if you have any questions or are unclear what data is being collected about you.

Our privacy notice for research participants provides more information on how the University of Southampton collects and uses your personal data when you take part in one of our research projects and can be found at:
http://www.southampton.ac.uk/assets/sharepoint/intranet/ls/Public/Research%20and%20Integrity%20Privacy%20Notice/Privacy%20Notice%20for%20Research%20Participants.pdf
Any personal data we collect in this study will be used only for the purposes of carrying out our research and will be handled according to the University's policies in line with data protection law. If any personal data is used from which you can be identified directly, it will not be disclosed to anyone else without your consent unless the University of Southampton is required by law to disclose it.

Data protection law requires us to have a valid legal reason ('lawful basis') to process and use your Personal data. The lawful basis for processing personal information in this research study is for the performance of a task carried out in the public interest. Personal data collected for research will not be used for any other purpose.

For the purposes of data protection law, the University of Southampton is the 'Data Controller' for this study, which means that we are responsible for looking after your information and using it properly. The University of Southampton will keep non-identifiable information about you for 25 years after the study has finished after which time any link between you and your information will be removed.

To safeguard your rights, we will use the minimum personal data necessary to achieve our research study objectives. Your data protection rights – such as to access, change, or transfer such information - may be limited, however, in order for the research output to be reliable and accurate. The University will not do anything with your personal data that you would not reasonably expect.

If you have any questions about how your personal data is used, or wish to exercise any of your rights, please consult the University's data protection webpage: (https://www.southampton.ac.uk/legalservices/what-we-do/data-protection-and-foi.page) where you can make a request using our online form. If you need further assistance, please contact the University's Data Protection Officer (data.protection@soton.ac.uk).

MANCAN2 PROCESS EVALUATION: PARTICIPANT INFORMATION SHEET

PROSTATE CANCER MEDICAL STAFF

MANCAN2 (MANaging symptoms during prostate CANcer treatment):
A multicentre randomised controlled trial (RCT) of a virtual self-help cognitive behavioural therapy (CBT) intervention to reduce the impact of hot flush and night sweat (HFNS) symptoms in men with prostate cancer undergoing androgen deprivation therapy (ADT)

YOUR PERCEPTIONS OF THE MANCAN2 STUDY

You are invited to take part in the MANCAN2 Process Evaluation. If you wish to participate, we will ask for you to take part in an interview with a member of the Southampton Clinical Trials Unit (SCTU) research team. The purpose of this interview is to understand your perceptions of the study intervention and to establish whether the intervention has potential to become a routine practice service.

What is the purpose of the MANCAN2 Study?

Half of all men that are diagnosed with prostate cancer receive treatment to reduce or block their levels of the male hormone, testosterone (called androgen deprivation therapy or ADT).

Although ADT is an effective treatment, it is associated with troublesome side effects that commonly include Hot Flushes and Night Sweats (HFNS). In fact, up to 80% of men undergoing ADT suffer from these.

HFNS can be both frequent and severe and can lead to a significant decrease in quality of life. They may also cause anxiety, low mood and sleep disturbances. Sometimes, they can be so severe and debilitating, that some men decide to stop treatment altogether.

Previous research has found that guided self-help Cognitive Behavioural Therapy (CBT) can be effective in reducing HFNS in men, when delivered by a clinical psychologist. We now want to find out if we can train NHS Cancer Nurse Specialists to deliver virtual guided self-help CBT and whether it is effective in reducing the impact of HFNS in men undergoing ADT.

MANCAN2 will test whether a 4-week guided self-help CBT intervention with pre and post group workshops (delivered virtually by the Cancer Nurse Specialist team), alongside the care that prostate patients would normally receive from their doctor, reduces the impact of HFNS more than normal care alone
For further details about the MANCAN2 trial, please refer to the enclosed patient participant information sheet.

What is the purpose of the questionnaires and interviews?

If MANCAN2 is found to be effective, there is potential to incorporate this intervention into currently existing staffing structures designed to support men living with and beyond cancer. Specifically, the guided self-help CBT could be delivered to patients (by their CNS team) as part of their usual treatment programme.
It is therefore important for us to understand site team attitudes, dynamics, perception of the intervention and ability to integrate it into current work.

Why have I been invited to take part?

You have been invited to take part because the <site name> MANCAN2 Principal Investigator <name> has identified you as a suitable person to approach for the interview.

Please note: *In order to be suitable, you must be a member of the medical team responsible for prostate care services, who is likely to impact on the adoption of interventions for routine care services.*

Do I have to take part?

No, you are free to decide whether or not to take part. If you decide to take part, you are still free to stop at any time without giving a reason. No questions will be asked if you stop. Furthermore, during the interview if there is a question you prefer not to answer, that question can be left out.

What will happen if I take part?

If you decide that you would like to take part, the first step is for you to complete the enclosed Informed Consent Form and Contact Details Form. Once you have completed, please send your completed forms to the research team at Southampton Clinical Trials Unit (SCTU) via email, using the secure University of Southampton safesend (instructions on how to send using safesend can be found on the Informed Consent Form).

Once you have returned your informed consent form and contacts form to us, a member of the SCTU research team will contact you. You will be invited to participate in an interview. The interview will take place within a month of the study finishing recruitment at your site and you will be asked questions regarding the processes involved in running the intervention, including:

1. Engagement with staff and patients
2. Post- intervention identification and treatment of men with HFNS and the potential for running the intervention in routine practice

The SCTU research team will arrange a convenient time with you to conduct a telephone (or video conference) interview lasting between 45 minutes and 1 hour. Interviews will be recorded.

What will happen after the interview?

The interview recording will be labelled with a code number and will be transferred securely to university approved transcriber who will type out everything you said in the interview. The transcriber

signs an agreement to keep everything you say in the interview confidential. We will remove any names, which would identify you or any other individuals.

The digital recording and the typed- up record (transcript), identified only by the code number, will be kept in a secure place with the University of Southampton.

What will happen to the results of the study?

At the end of the study we will submit our results to be published in medical journals. We may use some anonymised quotes from the information you gave us, but we will never include anything that would allow someone to identify you.

A summary of our research findings will be sent to everyone who requests to receive these. There is a section on the informed consent form where you can opt to receive the results.

Can I withdraw?

Yes. You have the right to change your mind and withdraw at any time without giving a reason and without your participant rights being affected. You can request for any data collected to be deleted.

However, it may not be possible for you to withdraw your data once the analysis has started because the data collected will already be pseudonymised and have been used but you can inform the research team if you do not want your anonymised interview content to be used in publications.

Are there any risks involved?

No, but you will be giving up some of your time to give the interview. You have the right not to answer any question and to ask for a break if you wish.

Will my participation and the information that I provide be kept confidential?

Yes. The interview recordings will be typed up and your name or any other details that can identify you will be removed, to ensure your privacy.

The recordings will be kept securely on the computer system at the University of Southampton, which only the research team can access. An audio recording of the interview will be sent to a University approved transcription company who will securely manage the audio recording and return a transcribed word document via email, where a copy will be stored on the secure network for analysis.

These anonymised transcripts (which don't include information which identifies who you are) will be stored on a password protected file on secure University of Southampton computers for up to 3 years. After this time, they will be destroyed. Interview recordings will be destroyed at the end of the study.

The findings from this study will be used in research report publications but no names will be included so any quotes used from the interview will be anonymous. We will remove any names, which would identify you or any other individuals.

Your contact details will be held separately from your interview information in a secure password-protected file on a University computer, we will use these details only to arrange the interview. These details will be deleted after your interview.

Data Protection Privacy Notice
See *Addendum 1* for information regarding the University of Southampton's Data Protection Privacy Notice.

What if there is a problem?

If you wish to complain, or have any concerns about any aspect of this study please contact the study Trial Manager by email (mancan2@soton.ac.uk) or telephone (023 8120 5589).

Who is organising and funding the research?

The study is being funded by the National Institute for Health Research (NIHR) Research for Patient Benefit (RfPB) programme. The study Sponsor is the University Hospital Southampton NHS Foundation Trust. The University of Southampton Clinical Trials Unit (SCTU) is responsible for oversight and management of the study.

Who has reviewed the study?

This study has been reviewed and approved by the <ethics committee> on <dd/mmm/yyyy> and the REC reference number is <insert>.

How can I find out more?

If you have any questions at this stage and would like to discuss them with the SCTU research team please call:

MANCAN2 Trial Manager: mancan2@soton.ac.uk. Tel: 023 8120 5589

Addendum 1: Data Protection Privacy Notice

The University Hospital Southampton NHS Foundation Trust are the Sponsor for this study and retain legal responsibility for the study. The Sponsor have delegated the responsibility of overall study coordination and data controller to the Southampton Clinical Trials Unit (SCTU).

SCTU are governed by the University of Southampton. The University of Southampton conducts research to the highest standards of research integrity. As a publicly-funded organisation, the University has to ensure that it is in the public interest when we use personally-identifiable information about people who have agreed to take part in research. This means that when you agree to take part in a research study, we will use information about you in the ways needed, and for the purposes specified, to conduct and complete the research project. Under data protection law, 'Personal data' means any information that relates to and is capable of identifying a living individual. The University's data protection policy governing the use of personal data by the University can be found on its website (https://www.southampton.ac.uk/legalservices/what-we-do/data-protection-and-foi.page).

This Participant Information Sheet tells you what data will be collected for this study and whether this includes any personal data. Please ask the research team if you have any questions or are unclear what data is being collected about you.

Our privacy notice for research participants provides more information on how the University of Southampton collects and uses your personal data when you take part in one of our research projects and can be found at:
http://www.southampton.ac.uk/assets/sharepoint/intranet/ls/Public/Research%20and%20Integrity%20Privacy%20Notice/Privacy%20Notice%20for%20Research%20Participants.pdf
Any personal data we collect in this study will be used only for the purposes of carrying out our research and will be handled according to the University's policies in line with data protection law. If any personal data is used from which you can be identified directly, it will not be disclosed to anyone else without your consent unless the University of Southampton is required by law to disclose it.

Data protection law requires us to have a valid legal reason ('lawful basis') to process and use your Personal data. The lawful basis for processing personal information in this research study is for the performance of a task carried out in the public interest. Personal data collected for research will not be used for any other purpose.

For the purposes of data protection law, the University of Southampton is the 'Data Controller' for this study, which means that we are responsible for looking after your information and using it properly. The University of Southampton will keep non- identifiable information about you for 25 years after the study has finished after which time any link between you and your information will be removed.

To safeguard your rights, we will use the minimum personal data necessary to achieve our research study objectives. Your data protection rights – such as to access, change, or transfer such information - may be limited, however, in order for the research output to be reliable and accurate. The University will not do anything with your personal data that you would not reasonably expect.
If you have any questions about how your personal data is used, or wish to exercise any of your rights, please consult the University's data protection webpage:
(https://www.southampton.ac.uk/legalservices/what-we-do/data-protection-and-foi.page) where you can make a request using our online form. If you need further assistance, please contact the University's Data Protection Officer (data.protection@soton.ac.uk).

Informed Consent Form

MANCAN2 PROCESS EVALUATION- INTERVIEW

<CNS / MEDIC/ MANAGER>

MANaging symptoms during prostate CANcer treatment (MANCAN2)
A multicentre randomised controlled trial (RCT) of a virtual self-help cognitive behavioural therapy (CBT) intervention to reduce the impact of hot flush and night sweat (HFNS) symptoms in men with prostate cancer undergoing androgen deprivation therapy (ADT).

Participant Identification Number ☐☐ - ☐☐

Please **add your initials** in each box to confirm that you have read and agree to the following statements:

1.	I confirm that I have read the **MANCAN2 Process Evaluation Participant Information Sheet** dated <dd/mmm/yyyy> Version <version number> for the above study.	Initials
2.	I confirm that I have had the opportunity to consider the information, ask questions and have had these answered satisfactorily.	Initials
3.	I understand that my participation in the study is voluntary and that I am free to withdraw at any time without giving any reason, and without my legal rights being affected.	Initials
4.	I understand that it may not be possible to withdraw interview data once analysis has started, and that I can inform the team that I do not want my anonymised interview content to be used in publications.	Initials
5.	I give permission for the interview to be recorded and for anonymised interview content to be used in publications. I understand that it will not be possible to identify me in any way.	Initials
6.	I understand that the recordings will be kept securely on the computer system at the University of Southampton, which only the research team can access. The recordings will be destroyed when the study is complete.	Initials

7.	I understand that the anonymised transcripts will be stored on a password protected file on secure University of Southampton computers for up to 3 years. After this time, they will be destroyed.	Initials
8.	I understand that the information collected about me may be used to support other research in the future, and may be shared anonymously with other researchers.	Initials
9.	**I agree to take part in the study and agree for my interview comments <(and study data (if CNS and will be completing the NoMAD questionnaire)> to be used for the purpose of this study.**	Initials
10.	**OPTIONAL:** **I wish to receive a summary of study's findings.**	YES Initials<br>NO Initials

Name of Participant	Signature	Date (DD-MMM-YYYY)
Name of SCTU researcher obtaining consent	Signature	Date (DD-MMM-YYYY)

Thank you. Please return this form to us using University of Southampton safesend.

To complete the form:

Please initial all the boxes, sign and date the form and send a scanned copy to the MANCAN2 trial manager using the University of Southampton's Safesend system (safesend.soton.ac.uk). Instructions on how to use Safesend are below.

Safesend is the safest and most secure way for you to send this form to the MANCAN2 trial manger as it is hosted by the University of Southampton and encrypts data both while it is being sent and once it has been received.

To use Safesend

1. Go to safesend.soton.ac.uk
2. Click the 'Drop-off button' and complete the required fields (your name, organisation and your work email address). SafeSend will send an email to your email with a link for you to follow. Follow the link.
3. Add the name: mancan2 and email mancan2@soton.ac.uk as your recipient. Click 'Add'. Then drag or click to add your contacts form, add a message if you like, then click 'drop-off files'. This will send your contacts form securely to the research team at SCTU.
