# Supplementary material for: A multicentre randomised controlled trial of a guided self-help cognitive behavioural therapy to MANage the impact of hot flushes and night sweats in patients with prostate CANcer undergoing androgen deprivation therapy (MANCAN2)
Source: Trials. 2023 Jul 10;24:450. doi: 10.1186/s13063-023-07325-w (PMC10332063; doi:10.1186/s13063-023-07325-w)
Supplement: Supplementary file 4 — Additional file 4. Patient Invitation Pack (including Patient Information Sheet and Informed Consent Form. [file 13063_2023_7325_MOESM4_ESM.pdf]

<Practice headed paper and Trust logo>

Dear _______________

MANaging symptoms during prostate CANcer treatment (MANCAN2 study).

We are writing to invite you to participate in a study. Enclosed is a Participant Information Sheet outlining the study and telling you more about why we have contacted you.

If you are interested in taking part, once you have read the patient information sheet, please complete and return the following documents directly to the research team at <insert site name>, using the FREEPOST envelop enclosed:

1. Informed Consent Form
2. Screening Questions
3. Contact Details Form
4. Baseline Questionnaires

The research team at <insert site name> will call you to discuss the study with you and answer any questions you may have. You can also get in touch with the research team if you have any questions at any time using the contact details provided in the enclosed participant information sheet.

If you do not want to take part, the research team would be grateful if you could let them know why by completing and returning the Study Decline form enclosed using the FREEPOST envelope provided. This is optional and anything that you say will not affect your medical care in any way.

Thank you for taking the time to read this letter and the enclosed participant information sheet.

Yours sincerely

[*Insert name*]
Principal Investigator MANCAN2 Study [*Insert Trust Name*

MANCAN2- Patient Contact Details Form

Thank you for reading the enclosed participant information sheet. If you are interested in taking part in MANCAN2 study, please complete this form and return to the research team using the FREEPOST envelope provided. By providing this information, you consent to the use of this data for the purpose and duration of the trial.

TO BE COMPLETED BY THE RESEARCH TEAM
Participant Identification Number ☐☐ - ☐☐

I am interested in taking part in the above study and am happy to be contacted.

Please note: as detailed in the participant information sheet and informed consent form, your hospital team will send your contacts form (via a safe and secure email) to the research team at Southampton Clinical Trials Unit (SCTU) so that a member of the research team can contact you about your study questionnaires and the optional study interview.

My contact details:

Name: ……………………………………………………………………………………………………

Address: ……………………………………………………………………………………………………

……………………………………………………………………………………………………

Post Code: …………………………..

Email Address* ……………………………………………………………………………………………………

Mobile phone number* ……………………………………………………………………………………

Home/work phone number: ………………………………………………………………………………

If there is a particular day of the week or time that is best for our research team to contact you, please write this below:

……

*****_Please note that these are required fields. If you do not have a mobile number, please provide your Home or Work telephone number. Thank you._

Please let us know your preferred method and time of contact:

Telephone Call ☐ Text☐ Email ☐

Preferred day/ time: ____

Thank you. Please return this form to us using the FREEPOST envelope enclosed.

Screening Questions

Thank you for reading through the Participant Information Sheet. The questions below will help us to see if this study is suitable for you. If you are interested in taking part in MANCAN2 study, please complete this form and return to the research team using the FREEPOST envelope provided. By providing this information, you consent to the use of this data for the purpose and duration of the trial.

TO BE COMPLETED BY THE RESEARCH TEAM
Participant Identification Number ☐☐ - ☐☐

Please read each question carefully and circle the answer that applies to you

Mancan2 Screening Questions:			
**1.**	**Are you able to read and understand English without assistance?**	**Yes**	**No**
**2.**	**Do you have access to the internet?**	**Yes**	**No**
**3.**	**If you are randomised to the guided self-help CBT intervention group, are you willing to attend virtual group workshops using video conferencing software (e.g. Zoom)?** *Don't worry if you do not already have the video conferencing software installed on your computer/ laptop/ mobile device as we will help you to download and access this.*	**Yes**	**No**

4. We would like to know a little more about your Hot flushes and night sweats.

During the past week, to what extent do you regard your flushes/sweats as a problem? Please circle a notch on the scale below.

No problem at all **Very much a problem**

1 2 3 4 5 6 7 8 9 10

Thank you. Please return this form to us using the FREEPOST envelope enclosed.

MANCAN2 Patient- Study Decline Form

Thank you for taking the time to read through the participant information sheet.

It's really useful for us to know the reasons why some men decide not to participate in the study. <u>If you do not want to participate in this study</u>, we'd be grateful if you could let us know the reason that you do not want to take part.

Please note: completion and return of this form is optional. By providing this information, you consent to the use of this data for the purpose and duration of the trial. The information that you provide will <u>not</u> affect your medical care in any way.

Name: ………………………………………………… Surname: …………………………………………………………

I do not want to take part in this study because:

□ I'm not interested

□ I don't have the internet

□ I don't have time

□ My Hot Flushes and Night Sweats are not problematic for me

□ I do not have Hot Flushes and Night Sweats

□ Other. *Please detail below:*

Thank you. Please return this form to us using the FREEPOST envelope enclosed.

<TO BE PRINTED ON LOCAL HOSPITAL HEADED PAPER>

MANCAN2- Participant Information Sheet

MANCAN2 (MANaging symptoms during prostate CANcer treatment):
A multicentre randomised controlled trial (RCT) of a virtual self-help cognitive behavioural therapy (CBT) intervention to reduce the impact of hot flush and night sweat (HFNS) symptoms in men with prostate cancer undergoing androgen deprivation therapy (ADT)

You are being invited to take part in a research study that is being run by the University Hospital Southampton NHS Foundation Trust, University of Southampton Clinical Trials Unit (SCTU) and <site name>. Before you make a decision, it may help to understand why the research is being done and what it would involve for you.

1. Why are we doing the MANCAN2 Trial?

Half of all men that are diagnosed with prostate cancer receive treatment to reduce or block their levels of the male hormone, testosterone (called androgen deprivation therapy or ADT).

Although ADT is an effective treatment, it is associated with troublesome side effects that commonly include Hot Flushes and Night Sweats (HFNS). In fact, up to 80% of men undergoing ADT suffer from these.

HFNS can be both frequent and severe and can lead to a significant decrease in quality of life. They may also cause anxiety, low mood and sleep disturbances. Sometimes, they can be so severe and debilitating, that some men decide to stop treatment altogether.

Previous research has found that guided self-help Cognitive Behavioural Therapy (CBT) can be effective in reducing HFNS in men, when delivered by a clinical psychologist. We now want to find out if we can train NHS Cancer Nurse Specialists to deliver guided self-help CBT and whether it is effective in reducing the impact of HFNS in men undergoing ADT.

This study will test whether a 4-week guided self-help CBT intervention with pre and post group workshops (delivered **either virtually or face-to-face)** by your hospital Cancer Nurse Specialist team), alongside the care that you would normally receive from your doctor, reduces the impact of HFNS more than normal care alone.

What we already know works…

CBT delivered by a
Clinical Psychologist

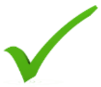

What MANCAN2 study will test…

Guided self-help CBT delivered by a member of your **Cancer Nurse Specialist Team**

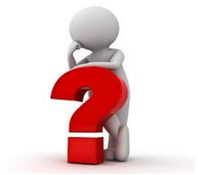

Participants

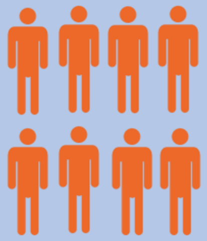

150 Men

6-8 Men per Group Workshop

Study Length

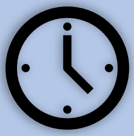

6 Months

Study Intervention

✓ **4-week self-help CBT (CBT Booklet + Audio)**

Two Group Workshops delivered by Cancer Nurse Specialist team. The workshops will either be virtual or Face to Face (or both). The research team will inform you of the planned method of workshop delivery before you agree to take part.

Study Questionnaires

All participants will be asked to complete study questionnaires at Baseline, 6-weeks and 6 months.

2. Why have I been invited?

You have been invited to take part because this study is for men with a diagnosis of prostate cancer who are currently receiving ADT.

To be eligible to take part, you must also be experiencing problematic HFNS. We do not know if you are experiencing HFNS and (if you are) we don't know how problematic they may be for you. That's why, if you are interested in taking part, we ask for you to complete and return the screening questions that are enclosed in this study invite pack.

Some of the screening questions will ask how problematic your HFNS are. We ask for this information to make sure that you are suitable to take part in the study.

Around 150 men in total will take part.

3. Who can take part?

The study is open to men who:

- Have a diagnosis of prostate cancer that is either Localised or at the Advanced disease stage. You may have had potentially curative treatments including, but not limited to, radiotherapy, brachytherapy or
- surgery. Are currently receiving ADT, and anticipated to require a minimum of 6 months further continuous treatment
- Are experiencing problematic HFNS symptoms (defined as a HFNS Rating Scale score of two or more)
- Are able to read and understand English without assistance
- Are 16 years or older
- Are able to attend **virtual or face-to-face** group workshops (virtual workshops will be delivered through video conferencing software).

Help will be provided with supporting you with accessing the conferencing software.

4. What is Cognitive Behavioural Therapy (CBT)?

Cognitive Behavioural Therapy (CBT) is widely used by psychologists and other healthcare professionals to help people manage many common difficulties such as anxiety, stress, pain or sleep problems. CBT can help you to explore and make changes to the ways that you think and behave in order to feel better and cope with your symptoms more effectively.

5. Do I have to take part?

No. It is up to you whether to take part in the study.

If you do not want to take part, we would be very grateful if you could let us know why by completing and returning the enclosed Study Decline Form using the FREEPOST envelope provided. This is optional and anything that you say will not affect your medical care in any way.

<u>Whether or not you choose to take part will not affect your medical care in anyway.</u>

6. What will happen to me if I take part and what do I have to do?

- If you decide that you are interested and would like to take part in the study, the first step is for you to sign the enclosed **Informed Consent Form** to indicate that you understand what the research involves and that you agree to take part.

- Once you have read and signed your consent form, we also ask for you to complete the enclosed **Contact Details Form, Screening Questions and your Baseline Questionnaires**. You should then return all four documents to the research team using the FREEPOST envelope enclosed. The research team will contact you to discuss the study with you and answer any questions you may have.

- A member of the hospital research team will check your responses to the screening questions to see if you are still eligible to take part in the study. The research team will contact you to collect some additional screening questions from you over the telephone.

- When enough men (12-16 men in total) have agreed to take part in the study (this may be up to 8 weeks after you initially expressed an interest) a member of the hospital research team will contact you by telephone to discuss the study and to confirm that you are still interested.

- You will then be allocated to one of two groups. The group that you go into will be decided by chance. Each person will have the same chance of being allocated to each of the groups, like pulling names out of a hat. The research team will inform you of the group that you have been allocated to.

  Group 1: Will receive treatment as usual **plus** a 4-week guided self-help CBT intervention with pre and post intervention group workshops.

  You will be given a self-help CBT Booklet to work through over the course of 4 weeks. The Booklet has four sections, and you will be asked to work through one section each week. You will be advised to set aside 30 minutes each day plus an additional 2 hours each week so that you can practice the new skills. You will also have access to a downloadable audio file/or CD demonstrating breathing and relaxation exercises.

  The group workshops will take place at week 1 and at week 4. The workshops will be delivered by a member of <insert site name> CNS Team. Workshops will be held on a

video conference platform and the groups will consist of 6-8 men. Each workshop will run for a duration of 1 hour 50 minutes.

To check everything was delivered to plan and as we expected by your hospital research team, we will take an audio recording of both group workshops. We will ask for you to consent to this before you enter the study. Anything that you say will not be judged, assessed nor will any information that you give during the workshops be used in the analysis or study publications. The workshops will be a place where you can safely share your thoughts without judgement.

Group 2: Will receive treatment as usual.

7. How long does the study take?

The study will last **6 months** from the time you are allocated to one of the groups. If you are randomised to group 1, then you will receive the study intervention for 4 weeks at the beginning of the study.

Both Groups will be asked to complete questionnaires at three time points; Baseline, 6 weeks and 6 months. The questionnaires will take approximately 20 minutes to complete at each occasion. If you have been allocated to group 1, you will be asked to complete an extra questionnaire at the end of your second group workshop.

The study research team at SCTU will post your questionnaires out to you and they may send text reminders and call you to complete your questionnaires with you over the telephone if they have not received these from you in the post.

8. Interviews (optional)

If you have been allocated to **group 1,** you will be invited to an optional interview to help us understand what it was like to take part in the guided self-help CBT. It doesn't matter if you have liked the study or not, all type of feedback is helpful.

If you are happy to be interviewed, we may contact you to arrange a time that suits you to be interviewed by telephone once you have finished the intervention. We will record the interview and it is expected to last between 45 minutes and 1 hour. These recordings will be typed up and your name or any other details that can identify you will be removed, to ensure your privacy.

You can still take part in the main study but decline the interview.

9. If I decide to take part, can I later change my mind?

Yes. You are free to withdraw from the study (including the optional interview) at any time. We will ask you why you have changed your mind in case there is anything we could do differently, but you do not have to tell us if you prefer not to. You will then receive usual NHS care. Your decision will not affect future treatment or prevent you from taking part in future research studies.

If you decide to withdraw from the trial, we will keep and use any data we have collected from you up to that point, but you will not be asked to complete any more assessments.

If you decide to withdraw from the optional interview, you can request for any data collected to be deleted. However, it may not be possible for you to withdraw your data once the analysis has started

because the data collected will already be pseudonymised and have been used but you can inform the research team if you do not want your anonymised interview content to be used in publications.

10. What are the benefits to taking part in this study?

Previous research has shown that guided self-help CBT (delivered by a clinical psychologist) to men undergoing prostate cancer treatments, is an effective way of reducing HFNS symptoms. If you are randomised to the group that receives the guided self-help CBT (delivered by your cancer nurse specialist team), it is possible that you will experience a similar benefit. If you are in the treatment at usual group, you may not benefit during the trial, but we hope that you will find it a positive experience. You may also benefit from the self-help CBT booklet and CD that will be offered to you at the end of the trial.

Whichever group you are randomised into, you will be helping to contribute to our understanding of how best to help men, with a diagnosis of prostate cancer undergoing ADT, who suffer with problematic HFNS.

11. Will I receive any payment?

You will not receive any monetary payment for taking part in this trial. However, if you participate in the interviews (this is optional) you will receive a £10 voucher.

12. What will happen at the end of the study?

At the end of the study, you will continue to receive usual support from your cancer nurse specialist and GP.

13. What will happen to the results of the study?

At the end of the study, we will submit our results to be published in medical journals. We may use some anonymised quotes from the information you provide (for example, if you decide to participate in the interviews too) but we will never include anything that would allow someone to identify you.

A summary of our research findings will be sent to everyone who requests to receive these. There is a section on the informed consent form where you can opt to receive the results.

14. Who is sponsoring and running the research?

The study is being funded by the National Institute for Health Research (NIHR) Research for Patient Benefit (RfPB) programme. The study Sponsor is the University Hospital Southampton NHS Foundation Trust. The University of Southampton Clinical Trials Unit (SCTU) act on behalf of the Sponsor and are responsible for oversight and management of the study.

15. Who has reviewed the study?

All research in the NHS is reviewed by an independent group of people, called a Research Ethics Committee, to protect your interests. This study has been reviewed by West Midlands - South Birmingham Research Ethics Committee who are happy for the study to proceed. The approval reference is 21/WM/0259.

16. Will my taking part in this study be kept confidential?

Yes. Your participation and the information we collect about you will be kept strictly confidential.

Notifying your GP

We will ask for your consent for the research team at <site name> to notify your GP of your participation in this study and to make a record of it in your medical notes.

Research Data

University Hospital Southampton NHS Foundation Trust is the sponsor for this study. The sponsor and the Southampton Clinical Trials Unit (SCTU), who act on behalf of the sponsor, will be using information from you and your medical records to conduct this study. They will act as the data controller for this study. This means that the sponsor and SCTU are responsible for looking after your information and using it properly. University Hospital Southampton NHS Foundation Trust will keep your non-identifiable research data for 15 years after the study has finished.

The sponsor and the Southampton Clinical Trials Unit (SCTU) may also use information provided by your GP. That is, in the unlikely event that you are hospitalised during the trial, the research team at <insert site name> may contact your GP to ascertain the cause and duration of your admission.

The research team at <insert site name> will collect information from you and your medical records for this research study in accordance with our instructions.

Non-identifiable research data, managed by the Southampton Clinical Trials Unit (SCTU), will be held on servers located in the EU and USA and access will be strictly controlled. All applicable Data Protection legislation will be abided by. Your study data is pseudonymised by assigning you a participant ID number, which is used to identify you during the study (on questionnaires for example) and for any participant-specific clarification between SCTU and your hospital. Only your initials will be stored in the study database.

In order to properly manage your data and ensure the research is reliable and accurate, your rights to view, change or move the research information we collect about you are limited. You can find out more about how we use your information on the Southampton Clinical Trials Unit website at https://www.southampton.ac.uk/ctu/about/index.page or you can contact the Southampton Clinical Trials Unit on 023 8120 5589 and ask to speak to the MANCAN2 team.

Individuals from University Hospital Southampton NHS Foundation Trust, the SCTU and regulatory organisations may look at your medical and research records to check the accuracy of the research study. <insert site name> will pass these details to University Hospital Southampton NHS Foundation Trust along with the information collected from you and your medical records. The only people in the University Hospital Southampton NHS Foundation Trust and the SCTU who will have access to information that identifies you will be those who need to audit the data collection process.

<insert site name> will keep your non-identifiable research data for 15 years after the study has finished. They will also keep your informed consent form for 15 years after the study has finished.

Contact Details Form and Informed Consent Form

So that a member of the research team at SCTU can contact you about your study questionnaires, we will ask for your consent for a copy of your contacts form and informed consent form to be sent securely from the research team at <insert site name> to the research team at University of Southampton Clinical Trials Unit (SCTU) where they will be stored and managed securely. The SCTU and <insert site name> will destroy your contacts forms (the original and the copy) at the end of the study.

Group Workshop Recordings

We will ask for your consent for your CNS team to record the group workshop. The audio recordings will be sent securely to Southampton Clinical Trials Unit (where they will be stored and managed securely). The information that you share during your group sessions will not be used in the study analysis nor will it be published in any medical journal.

Interview Recordings

If you consent to participate in the optional interview, we will ask for your consent to record the interview. The interview recordings will be typed up and your name or any other details that can identify you will be removed, to ensure your privacy. They will be stored securely on the computer system at Southampton Clinical Trials Unit , which only the study research team can access. Prior to analysis, a copy of the audio recording will be sent securely to a University approved transcription company who will securely manage the audio recording and securely return a transcribed word document.

The anonymised transcripts (which don't include information which identifies who you are) will be stored securely on University of Southampton computers for up to 3 years. After this time, they will be destroyed. The audio recordings will be destroyed when the study is complete.

The findings from this study will be submitted to be published in medical journals, but no names will be included so any quotes from the interview will be anonymous. We will remove any names, which would identify you or any other individuals.

17. What if there is a problem?

If you have a concern about any aspect of this study, if there is an emergency or you are in need of some help and advice please contact either the study research nurse or prostate cancer CNS who will do their best to help you [<insert email> and <contact number> for site research team]. If you would like to discuss your concerns or wish to seek advice from someone outside of the study team then we advise for you to please contact your doctor or GP.

You can seek support from charities such as Prostate Cancer UK (PCUK). Please visit their website for further information about the types of support they can offer: https://prostate canceruk.org.

If you remain unhappy and wish to complain formally, you can do this by contacting your local Patients Advice and Liaison Service Office. Details can be obtained from www.pals.nhs.uk.

In the event that something does go wrong and you are harmed during the research and this is due to someone's negligence then you may have grounds for a legal action for compensation against University Hospital Southampton NHS Foundation Trust but you may have to pay your legal costs. The normal National Health Service complaints mechanisms will still be available to you (if appropriate).

How can I find out more?

If you have any questions at this stage (or during the study) and would like to discuss them with a study research nurse or cancer nurse specialist, please contact:

Study Cancer Nurse Specialist: [local PI name]. Tel: [local PI phone number]

Study Research Nurse: [research nurse name]. Tel: [research nurse phone number]

You may also wish to take some time to speak to your family and friends or your GP before you make a decision.

TODAY

Read Information Leaflet & Ask Questions

The research team will call you to discuss the study with you and answer any questions you may have. You can get in touch with the research team if you have any questions at any time using the contact details provided in this participant information sheet.

A member of the research team will check your responses to the screening questions to check that you are still eligible to take part in the study. The research team will contact you to collect some additional screening questions from you over the telephone.

Express interest in taking part by completing and returning your **Informed Consent Form; Contacts Form; Screening Questions and Baseline Questionnaires** to the research team at <site name> using the FREEPOST envelope provided. 111

Decide not to take part. We would be grateful if you could complete and return the enclosed **study decline form** (this is optional) using the FREEPOST envelope provided. You will continue with your treatment as usual.

1-8 weeks from Today — **Trial Starts**

You will be Randomly assigned to one of two Groups:

Group 1: guided self-help CBT

A total of 6-8 men will be in this Group.

You will be invited to participate in 2 group workshops with other men in your group and a Cancer Nurse Specialist.

You will still continue with your treatment as usual from your CNS/ GP.

Group 2: Treatment as Usual

A total of 6-8 men will be in this Group

If you are Randomised to this group you will continue with your treatment as usual from your CNS/ GP.

	Group 1	Group 2
**Week 1**	**Treatment as Usual plus Group Workshop 1** and completion of 25% of your CBT self-help booklet.	Treatment as Usual.
**Week 2**	**Treatment as Usual plus** Completion of 25% of your CBT self-help booklet.	Treatment as Usual.
**Week 3**	**Treatment as Usual plus** Completion of 25% of your CBT self-help booklet.	Treatment as Usual.
**Week 4**	**Treatment as Usual plus self-help CBT session 2** and completion of 25% of your CBT self-help booklet. You will also complete an evaluation questionnaire at the end of your group session.	Treatment as Usual.
**Week 6**	Complete your 6-week questionnaires by post or over the telephone.	Complete your 6-week questionnaires by post or over the telephone.
**6 Months**	Complete your 6-month questionnaires by post or over the telephone.	Complete your 6-month questionnaires by post or over the telephone.

<TO BE PRINTED ON LOCAL HOSPITAL HEADED PAPER>

Informed Consent Form: MANCAN2

MANaging symptoms during prostate CANcer treatment (MANCAN2)
A multicentre randomised controlled trial (RCT) of a virtual self-help cognitive behavioural therapy (CBT) intervention to reduce the impact of hot flush and night sweat (HFNS) symptoms in men with prostate cancer undergoing androgen deprivation therapy (ADT).

Participant Identification Number [ ][ ] - [ ][ ]

Please **add your initials** in each box to confirm that you have read and agree to the following statements:

1.	I confirm that I have read the Participant Information Sheet dated <dd/mmm/yyyy>Version <version number> for the above study. I have had the opportunity to consider the information, ask questions over the phone or face to face and have had these answered satisfactorily.	Initials
2.	I understand that my participation is voluntary and that I am free to withdraw at any time without giving any reason, without my medical care or legal rights being affected.	Initials
3.	I understand that should I withdraw from the study then the information collected about me up to this point may still be used for the purposes of achieving the objectives of the study.	Initials
4.	I consent to be contacted by the Research Team (based at my hospital and Southampton Clinical Trials Unit) as is required for the study, and I understand that my name, postal and email addresses and phone number will be stored securely and will not be revealed to anyone outside of the Research Team.	Initials
5.	I give permission for a copy of my consent form to be shared with and sent securely from the hospital research team to the Southampton Clinical Trials Unit (where it will be stored securely), to allow confirmation of my consent.	Initials
6.	I give permission for my contact details to be shared with and sent securely from the hospital research team to the Southampton Clinical Trials Unit (where stored securely) to allow the Southampton Clinical Trials Unit research team to contact me.	Initials

Questionnaires

Baseline

**Date**	D	D	M	M	M	Y	Y	Y	Y

**Participant ID**					-			

**Participant Initials**			

Thank you for agreeing to take part in MANCAN2. Once you have completed this questionnaire pack please return it using the pre-paid envelope provided. If you have any questions or require any assistance, please contact the Research Team on:

<insert site postal address>

Email: <insert site email>
Tel: <insert site Tel>

Blank page

Thank you for taking part in MANCAN2. Your responses in this questionnaire will really help us to understand how best to help men, with prostate cancer who suffer with Hot Flushes and Night Sweats.

In this questionnaire, you are asked to complete questions about yourself and your hot flushes and night sweats and how they affect you, as well as some more general questions about your life.

The questionnaire is divided into **9 sections**. It may seem that there are many questions and that there is repetition; however, it is important that you try to answer all questions in all sections. If you find you cannot answer one, or would prefer not to, please leave it out and go on to the next question.

The questionnaire should take about 20 minutes to complete. Some questions ask you to respond in different ways, so please do read the instructions for each section.

Please try to return your completed questionnaires within one week. All of the questionnaires are really important. If for any reason, you cannot find the time to complete the rest of the sections, please return everything you have done in the FREEPOST envelope. A researcher will then contact you to complete the rest of the questions over the phone, if you are happy to do so.

There are no right or wrong answers.

Your answers to the questions will, of course, be treated as strictly confidential. If you have any questions or problems completing the questionnaire, please contact the research team: <insert site contact>

It is possible that you may find some of these questions raise some issues for you which may be upsetting. Please be aware that what you have written is not always read and analysed until sometime after we receive your questionnaire, and these are not read by your health care team. If you have any concerns following completion of the questionnaire, we recommend that you seek support from your health care providers, such as your GP or specialist nurse.

Best Wishes

The MANCAN2 Research Team

Please note there are questions on each side of the pages.

Please turn this page to begin the questionnaire. Thank you.

Date: d d m m m y y y y   ID: _ _ _ _ - _ _ _   Initials: _ _ _

Section 1 – About You

1. What is your Date of Birth M M M Y Y Y Y

2. Weight ☐☐ **Stone** ☐☐ **Lbs**

3. Height ☐ **Feet** ☐☐ **Inches**

4. How would you describe your <u>ethnic group</u>?

Please tick the item that best describes your ethnic group

White:

English/ Welsh/ Scottish/ Northern Irish/ British ☐

Irish ☐

Romani or Irish Traveller ☐

Any other White background ☐

Mixed/ Multiple ethnic groups:

White and Black Caribbean ☐

White and Black African ☐

White and Asian ☐

Any other Mixed/ Multiple ethnic background ☐

Asian/ Asian British:

Indian ☐

Pakistani ☐

Bangladeshi ☐

Chinese ☐

Any other Asian background ☐

Black/ African/ Caribbean/ Black British:

African ☐

Caribbean ☐

Any other Black/ African/ Caribbean background ☐

Arab ☐

Any other ethnic group ☐

5. Which of the following best describes your current employment status?

Please tick one box or state "Other"

Working full time (30 hours or more a week) ☐

Working part time (less than 30 hours a week) ☐

Unemployed ☐

Retired ☐

Other: ______________________

6. What is the highest level of education that you have completed?

Please tick one box

Post- graduate degree (Master's or PhD), or equivalent in professional training ☐

Undergraduate degree (Bachelor's degree), or equivalent in professional training ☐

A-levels, BTEC, vocational level 3 or equivalents ☐

GCSEs/ O Level, vocational level 2 or equivalents ☐

Qualifications at level 1 and below ☐

No Qualifications ☐

7. What is your relationship status?

Please tick one box

Single ☐

Have a partner but don't live with them ☐

Married/ living with partner ☐

Divorced/ Separated ☐

Widower ☐

8. What is your smoking habit status?

Please tick one box

Current Smoker ☐

Ex-Smoker ☐

Never Smoked ☐

9. How many units of alcohol do you drink on average each week?

One unit = small glass of wine, half pint of beer or one small measure of spirits

Please tick one box

None ☐

1-6 ☐

7-13 ☐

14+ ☐

10. How often do you do any exercise?

Please tick one box

Rarely/ never ☐

Less than once a week ☐

Once a week ☐

2-3 times a week ☐

4-6 times a week ☐

Every day ☐

Section 2- Hot Flushes and Night Sweats (HFNS)

1a). How often have you had hot flushes in the past week?

Please estimate: ☐☐ times each day **OR** ☐☐ times each week

1b). On average how severe have your hot flushes been in the last week?

Please tick: Mild ☐ Moderate ☐ Severe ☐

Not Experienced Any ☐

2a). If you have night sweats, how often have they woken you up in the past week?

Please estimate: ☐☐ times each night **OR** ☐☐ times each week

2b). On average how severe have your night sweats been in the last week?

Please tick: Mild ☐ Moderate ☐ Severe ☐

Not Experienced Any ☐

Date: | d | d | m | m | m | y | y | y | y | ID: | | | | | - | | | | Initials: | | | |

Please circle a number on each scale to indicate how your flushes/sweats have been during the past week:

3. To what extent do you regard your flushes/sweats as a problem?

No problem at all — Very much a problem

1 2 3 4 5 6 7 8 9 10

4. How distressed do you feel about your hot flushes?

Not distressed at all — Very distressed indeed

1 2 3 4 5 6 7 8 9 10

5. How much do your hot flushes interfere with your daily routine?

Not at all — Very much indeed

1 2 3 4 5 6 7 8 9 10

6. How much has your sleep been disrupted by night sweats?

Not at all — Very much indeed

1 2 3 4 5 6 7 8 9 10

Date: | d | d | m | m | m | y | y | y | y | ID: | | | | | - | | | | Initials: | | | |

This questionnaire lists beliefs about hot flushes and night sweats. Please **circle** the response that best describes how much you agree or disagree with each statement based on your beliefs and reactions to your flushes and sweats **in the past 2 weeks**. There are no right or wrong answers.

		Strongly Disagree	Moderately disagree	Mildly disagree	Mildly agree	Moderately agree	Strongly agree
1	When I have night sweats it is harder to cope the next day.	0	1	2	3	4	5
2	I should not have to put up with hot flushes I shouldn't have them.	0	1	2	3	4	5
3	When I have hot flushes I worry about what other people will think of me	0	1	2	3	4	5
4	At least hot flushes mean that my cancer is being treated	0	1	2	3	4	5
5	Having a hot flush makes me feel less masculine	0	1	2	3	4	5
6	I find my night sweats troublesome to manage	0	1	2	3	4	5
7	When I have a hot flush, I am embarrassed	0	1	2	3	4	5
8	When I have a hot flush, other people will be able to tell that I am unwell	0	1	2	3	4	5
9	When I have night sweats I won't be able to get back to sleep	0	1	2	3	4	5
10	When I have hot flushes or night sweats I try to accept them	0	1	2	3	4	5
11	Night sweats and disrupted sleep affect my general health	0	1	2	3	4	5
12	When I have a hot flush, I try to be calm and relaxed	0	1	2	3	4	5
13	I use humour to deal with hot flushes	0	1	2	3	4	5
14	I take action to cooldown (cold drinks, take off layers) when I have a flush	0	1	2	3	4	5
15	When I have hot flushes, I carry on and ignore them	0	1	2	3	4	5

16	I have to leave or avoid some social situations because of hot flushes	0	1	2	3	4	5
17	I deal with my hot flushes by being open and talking about them with other people	0	1	2	3	4	5

Section 3- GAD-7

Over the last 2 weeks, how often have you been bothered by any of the following problems?

		Not at all	Several days	More than half the days	Nearly every day
1.	Feeling nervous, anxious or on edge	☐	☐	☐	☐
2.	Not being able to stop or control worrying	☐	☐	☐	☐
3.	Worrying too much about different things	☐	☐	☐	☐
4.	Trouble relaxing	☐	☐	☐	☐
5.	Being so restless that it is hard to sit still	☐	☐	☐	☐
6.	Becoming easily annoyed or irritable	☐	☐	☐	☐
7.	Feeling afraid as if something awful might happen	☐	☐	☐	☐

Section 4- PHQ-9

Over the last 2 weeks, how often have you been bothered by any of the following problems?

		Not at all	Several days	More than half the days	Nearly every day
1.	Little interest or pleasure in doing things	☐	☐	☐	☐
2.	Feeling down, depressed, or hopeless	☐	☐	☐	☐
3.	Trouble falling or staying asleep, or sleeping too much	☐	☐	☐	☐
4.	Feeling tired or having little energy	☐	☐	☐	☐
5.	Poor appetite or overeating	☐	☐	☐	☐
6.	Feeling bad about yourself — or that you are a failure or have let yourself or your family down	☐	☐	☐	☐
7.	Trouble concentrating on things, such as reading the newspaper or watching television	☐	☐	☐	☐
8.	Moving or speaking so slowly that other people could have noticed? Or the opposite — being so fidgety or restless that you have been moving around a lot more than usual	☐	☐	☐	☐
9.	Thoughts that you would be better off dead or of hurting yourself in some way	☐	☐	☐	☐

If you checked off any problems, how difficult have these problems made it for you to do your work, take care of things at home, or get along with other people?

Not difficult at all	Somewhat difficult	Very difficult	Extremely difficult
☐	☐	☐	☐

Section 5- PSQI

During the past month, how would you rate your sleep quality overall?

Very good ☐

Fairly good ☐

Fairly bad ☐

Very Bad ☐

Section 6- WSAS

People's problems sometimes affect their ability to do certain day-to-day tasks in their lives. To rate your problems, look at each section and determine on the scale provided how much your problem impairs your ability to carry out the activity.

1. Because of my hot flushes and night sweats my **ability to work** is impaired. '0' means 'not at all impaired' and '8' means very severely impaired to the point I can't work. (if you are retired or choose not to have a job for reasons unrelated to your problem, please tick N/A (not applicable))

0 1 2 3 4 5 6 7 8 N/A ☐

Not at all   Slightly   Definitely   Markedly   Very Severely

2. Because of my hot flushes and night sweats my **home management** (cleaning, tidying, shopping, cooking, looking after home/ children, paying bills etc) is impaired.

0 1 2 3 4 5 6 7 8

Not at all   Slightly   Definitely   Markedly   Very Severely

3. Because of my hot flushes and night sweats my **social leisure activities** (with other people, e.g. parties, pubs, outings, entertaining etc) are impaired.

0	1	2	3	4	5	6	7	8

Not at all **Slightly** **Definitely** **Markedly** **Very Severely**

4. Because of my hot flushes and night sweats my **private leisure activities** (done alone, e.g. reading, gardening, sewing, hobbies, walking alone etc) are impaired.

0	1	2	3	4	5	6	7	8

Not at all **Slightly** **Definitely** **Markedly** **Very Severely**

5. Because of my hot flushes and night sweats my ability to form and maintain **close relationships** with others, including those I live with, is impaired.

0	1	2	3	4	5	6	7	8

Not at all **Slightly** **Definitely** **Markedly** **Very Severely**

Date: | d | d | m | m | m | y | y | y | y | ID: | | | | | - | | | | Initials: | | | |

Section 7 – EORTC QLQ- C30

We are interested in some things about you and your health. Please answer all of the questions yourself by **circling** the number that best applies to you. There are no "right" or "wrong" answers. The information that you provide will remain strictly confidential.

		Not At all	A Little	Quite A Bit	Very Much
1.	Do you have any trouble doing strenuous activities, like carrying a heavy shopping bag or a suitcase?	1	2	3	4
2.	Do you have any trouble taking a long walk?	1	2	3	4
3.	Do you have any trouble taking a short walk outside of the house?	1	2	3	4
4.	Do you need to stay in bed or a chair during the day?	1	2	3	4
5.	Do you need help with eating, dressing, washing yourself or using the toilet?	1	2	3	4

During the past week:

		Not At all	A Little	Quite A Bit	Very Much
6.	Were you limited in doing either your work or other daily activities?	1	2	3	4
7.	Were you limited in pursuing your hobbies or other leisure time activities?	1	2	3	4
8.	Were you short of breath?	1	2	3	4
9.	Have you had pain?	1	2	3	4
10.	Did you need to rest?	1	2	3	4
11.	Have you had trouble sleeping?	1	2	3	4

Date: | d | d | m | m | m | y | y | y | y | ID: | | | | | - | | | | Initials: | | | |

During the past week:		Not At all	A Little	Quite A Bit	Very Much
12.	Have you felt weak?	1	2	3	4
13.	Have you lacked appetite?	1	2	3	4
14.	Have you felt nauseated?	1	2	3	4
15.	Have you vomited?	1	2	3	4
16.	Have you been constipated?	1	2	3	4
17.	Have you had diarrhea?	1	2	3	4
18.	Were you tired?	1	2	3	4
19.	Did pain interfere with your daily activities?	1	2	3	4
20.	Have you had difficulty in concentrating on things, like reading a newspaper or watching television?	1	2	3	4
21.	Did you feel tense?	1	2	3	4
22.	Did you worry?	1	2	3	4
23.	Did you feel irritable?	1	2	3	4
24.	Did you feel depressed?	1	2	3	4
25.	Have you had difficulty remembering things?	1	2	3	4
26.	Has your physical condition or medical treatment interfered with your <u>family</u> life?	1	2	3	4
27.	Has your physical condition or medical treatment interfered with your <u>social</u> activities?	1	2	3	4
28.	Has your physical condition or medical treatment caused you financial difficulties?	1	2	3	4

Please turn this page to complete the remaining questions (back page) Thank you.

For the following questions please circle the number between 1 and 7 that best applies to you.

29. How would you rate your overall <u>health</u> during the past week?

0 1 2 3 4 5 6 7

Very poor **Excellent**

30. How would you rate your overall <u>quality of life</u> during the past week?

0 1 2 3 4 5 6 7

Very poor **Excellent**

Section 8- Hormonal Treatment for your Prostate Cancer

Have you continued with your hormonal injections, given by your GP, as planned? (delays up to 1-2 weeks are fine)

Yes ☐ No ☐

If No, please tell us why? ……………………………………………………………………………………

Section 9- Overnight Stay in Hospital

Since you have consented to take part in this study, have you been so ill that you stayed overnight as an inpatient in an NHS or private Hospital? *This does not include planned treatments or hospital visits.*

Yes ☐ No ☐

Note: If you answered Yes, we will contact your GP to ask them why you were hospitalised and how long for.

Thank you.

Please return your questionnaire to the Research Team at the Clatterbridge Cancer Centre, using the FREEPOST envelope provided.

7.	I understand that the group workshops will be recorded to help the research team at Southampton Clinical Trials Unit measure whether everything was delivered to plan and as expected, by the hospital research team. I understand that anything that I say will be kept confidential and only the study team will have access to the recordings. I also understand that anything that I say will not be assessed nor will any information that I give during the workshops be used in the analysis or study publications.	Initials
8.	I give consent to be audio recorded (if I am randomised to the study CBT intervention workshops) and for the audio file to be shared with and sent securely from the hospital research team to Southampton Clinical Trials Unit (where they will be stored and managed securely).	Initials
9.	I understand that relevant sections of my medical notes and data collected during the study may be looked at by individuals from the Sponsor, or their delegates; regulatory authorities; or from the NHS Trust, where it is relevant to my taking part in this research. I give permission for these individuals to have access to my records.	Initials
10.	I agree to my General Practitioner being informed of my participation in the study and to receiving information critical to my care. In the unlikely event that I am hospitalised during the trial for unforeseen reasons, I give my consent for the research team to contact my GP to ascertain the cause and duration of my admission.	Initials
11.	I understand that the information collected about me may be used to support other research in the future and may be shared anonymously with other researchers running other research studies in this organisation and in other organisations. These organisations may be universities or NHS organisations in this country or abroad.	Initials

The following statements are optional:

**12**	**OPTIONAL:** I agree to being informed of the results of the MANCAN2 study.	YES Initials<br>NO Initials

**12.**	**OPTIONAL:** I consent to be contacted during the study by the Research Team to be interviewed about my experiences of being part of the MANCAN2 Study.	YES Initials<br>NO Initials
**13.**	**If you consented to be interviewed (Item 14):** I give permission for the interview to be recorded and for anonymised interview content to be used in publications. I understand that it will not be possible to identify me in any way.	Initials
**14.**	**If you consented to be interviewed:** I understand that my participation is voluntary and that I am free to withdraw at any time without giving any reason, without my medical care or legal rights being affected. I can request for any data collected to be deleted. However, I understand that it will not be possible for me to withdraw my data once the analysis has started because the data collected will already be pseudonymised and have been used.	Initials
**15.**	**If you consented to be interviewed:** I understand that the recordings will be kept securely on the computer system at the University of Southampton, which only the research team can access. The recordings will be destroyed when the study is complete.	Initials
**16.**	**If you consented to be interviewed**: I understand that the anonymised transcripts will be stored on a password protected file on secure University of Southampton computers for up to 3 years. After this time, they will be destroyed.	Initials

Please type your initials in the box below to confirm that you agree to take part in the study:

**17.**	**I agree to take part in the MANCAN2 study and agree for my data to be used for the purpose of this study.**	Initials

Name of Participant	Signature	Date (DD-MMM-YYYY)
Name of researcher obtaining consent	Signature	Date (DD-MMM-YYYY)

Thank you. Please return this form to us using the FREEPOST envelope enclosed.

Reminder for Research Team:

- Original signed consent form to be kept in the Investigator Site File
- 1 photocopy given to the participant
- 1 photocopy filed in the participant's medical records
- 1 scanned copy to be emailed to the Southampton CTU via secure safesend, for central monitoring purposes

Data Protection Privacy Notice

The University Hospital Southampton NHS Foundation Trust are the Sponsor for this study and retain legal responsibility for the study. The Sponsor have delegated the responsibility of overall study coordination and data controller to the Southampton Clinical Trials Unit (SCTU).

SCTU are governed by the University of Southampton. The University of Southampton conducts research to the highest standards of research integrity. As a publicly funded organisation, the University has to ensure that it is in the public interest when we use personally-identifiable information about people who have agreed to take part in research. This means that when you agree to take part in a research study, we will use information about you in the ways needed, and for the purposes specified, to conduct and complete the research project. Under data protection law, 'Personal data' means any information that relates to and is capable of identifying a living individual. The University's data protection policy governing the use of personal data by the University can be found on its website (https://www.southampton.ac.uk/legalservices/what-we-do/data-protection-and-foi.page).

This Participant Information Sheet tells you what data will be collected for this study and whether this includes any personal data. Please ask the research team if you have any questions or are unclear what data is being collected about you.

Our privacy notice for research participants provides more information on how the University of Southampton collects and uses your personal data when you take part in one of our research projects
and can be found at:
http://www.southampton.ac.uk/assets/sharepoint/intranet/ls/Public/Research%20and%20Integrity%20Privacy%20Notice/Privacy%20Notice%20for%20Research%20Participants.pdf
Any personal data we collect in this study will be used only for the purposes of carrying out our research and will be handled according to the University's policies in line with data protection law. If any personal data is used from which you can be identified directly, it will not be disclosed to anyone else without your consent unless the University of Southampton is required by law to disclose it.

Data protection law requires us to have a valid legal reason ('lawful basis') to process and use your Personal data. The lawful basis for processing personal information in this research study is for the performance of a task carried out in the public interest. Personal data collected for research will not be used for any other purpose.

For the purposes of data protection law, the University of Southampton is the 'Data Controller' for this study, which means that we are responsible for looking after your information and using it properly. The University of Southampton will keep your research data for 15 years after the study has finished after which time any link between you and your information will be removed. Identifiable information (such as your contact details) will be destroyed at the end of the study.

Your hospital will keep identifiable information about you from this study for 15 years after the study has finished. To safeguard your rights, we will use the minimum personal data necessary to achieve our research study objectives. Your data protection rights – such as to access, change, or transfer such information - may be limited, however, in order for the research output to be reliable and accurate. The University will not do anything with your personal data that you would not reasonably expect. If you have any questions about how your personal data is used, or wish to exercise any of your rights, please consult the University's data protection webpage:

(https://www.southampton.ac.uk/legalservices/what-we-do/data-protection-and-foi.page) where you can make a request using our online form. If you need further assistance, please contact the University's Data Protection Officer (data.protection@soton.ac.uk).
