# Supplementary material for: A multicentre randomised controlled trial of a guided self-help cognitive behavioural therapy to MANage the impact of hot flushes and night sweats in patients with prostate CANcer undergoing androgen deprivation therapy (MANCAN2)
Source: Trials. 2023 Jul 10;24:450. doi: 10.1186/s13063-023-07325-w (PMC10332063; doi:10.1186/s13063-023-07325-w)
Supplement: Supplementary file 6 — Additional file 6. Full Protocol [file 13063_2023_7325_MOESM6_ESM.pdf]

MANCAN2

A multicentre randomised controlled trial of guided self-help cognitive behavioural therapy to MANage the impact of hot flush and night sweat symptoms in patients with prostate CANcer undergoing androgen deprivation therapy (MANCAN2)

Version 4 23 Feb 2023

SPONSOR: University Hospital Southampton NHS Foundation Trust
COORDINATING CENTRE: Southampton Clinical Trials Unit

IRAS number:	304500
ISRCTN reference:	58720120
Ethics reference number:	21/WM/0259
Sponsor reference number:	RHM CAN1636
Funder reference number:	NIHRR201542
ICD10	[Insert]

Protocol authorised by:

**Name:**	Dr Simon Crabb	**Role:**	Chief Investigator
**Signature:**		**Date:**	
**Name:**	Prof Gareth Griffiths	**Role:**	Director of SCTU
**Signature:**		**Date:**	
**Name:**	Sharon Davies-Dear	**Role:**	On behalf of Sponsor
**Signature:**		**Date:**	

MAIN TRIAL CONTACT

Chief Investigator: Dr Simon Crabb

Address: Southampton Clinical Trials Unit
Southampton General Hospital
Tremona Road
Southampton
SO16 6YD

Tel: 023 8120 5170
Email: s.j.crabb@southampton.ac.uk

TRIAL COORDINATION CENTRE

For general trial and clinical queries e.g. participant queries, trial supplies, data collection, please contact in the first instance:

MANCAN2 Trial Manager
Tel: 023 8120 5589
Email: mancan2@soton.ac.uk

Address: Southampton Clinical Trials Unit
Southampton General Hospital
Tremona Road
Southampton
SO16 6YD

Tel: 023 8120 5154
Fax: ctu@soton.ac.uk
Email: www.southampton.ac.uk/ctu
Web:

SPONSOR

University Hospital Southampton NHS Foundation Trust is the research sponsor for this trial. For further information regarding sponsorship conditions, please contact Sharon Davies-Dear:

Address: Research & Development Office
E level, SCBR,
Laboratory & Pathology Block, MP 138
Southampton General Hospital
Tremona Road
Southampton

Tel: 023 8120 5314
Web: www.uhs.nhs.uk

CO-INVESTIGATORS

Co-Investigators can be contacted via the Trial Coordination Centre.

Prof Deborah Fenlon, Swansea University
Ms Jacqui Nuttall, University of Southampton (UoS)
Dr Jonathan Martin, UoS
Prof Myra Hunter, King's College London
Prof Alison Richardson, UoS
Dr Sean Ewings, UoS
Roger Bacon, Prostate Cancer Support Organisation
Prof James Raftery, UoS
Dr Evgenia Stefanopoulou, Turning Point
Louisa Fleure, GSTT

FUNDER

This trial is funded by the National Institute for Health Research (NIHR) Research for Patient Benefit (RfPB) programme.

Protocol Information

This protocol describes the MANCAN2 Trial and provides information about procedures for entering participants. The protocol should not be used as a guide for the treatment of other non- Trial participants; every care was taken in its drafting, but corrections or amendments may be necessary. These will be circulated to investigators in the study, but sites entering participants for the first time are advised to contact Southampton Clinical Trials Unit to confirm they have the most recent version.

Compliance

This Trial will adhere to the principles of Good Clinical Practice (GCP). It will be conducted in compliance with the protocol, the current Data Protection Regulations and all other regulatory requirements, as appropriate.

Table of Contents

LIST OF ABBREVIATIONS 6

KEYWORDS 7

TRIAL SYNOPSIS 8

TRIAL SCHEMA 11

TABLE 1- SCHEDULE OF OBSERVATIONS AND PROCEDURES 12

1 INTRODUCTION 16

1.1 BACKGROUND 16

1.2 RATIONALE AND RISK BENEFITS FOR CURRENT TRIAL 16

2 TRIAL OBJECTIVES 19

3 TRIAL DESIGN 21

3.1 RANDOMISED CONTROLLED TRIAL 21

3.1.1 Design 21

3.1.2 Settings 21

3.1.3 Target population 21

3.1.4 Intervention being assessed 21

3.1.5 Trial outcome measures 21

3.1.6 Health economic evaluation 22

3.2 PROCESS EVALUATION 23

3.3 CENTRE SELECTION 23

3.4 DEFINITION OF END OF TRIAL 23

4 SELECTION AND ENROLMENT OF PARTICIPANTS 24

4.1 PATIENT ENROLLMENT AND CONSENT 24

4.1.1 Participant identification 24

4.1.2 Informed consent procedure 24

4.1.3 Screening Procedures 25

4.1.4 Completion of Baseline Questionnaires 25

4.2 INCLUSION CRITERIA 26

4.3 EXCLUSION CRITERIA 26

4.4 SCREENING FAILURES 26

4.5 REGISTRATION AND RANDOMISATION PROCEDURES 27

5 TRIAL OBSERVATIONS AND PROCEDURES 28

5.1 BASELINE DATA COLLECTION 28

5.2 TRIAL PROCEDURES 28

5.2.1 Training 29

5.2.2 Supervision 30

5.2.3 Adherence 30

5.2.4 Fidelity 30

5.3 DATA COLLECTION/ASSESSMENT 31

5.3.1 Psychosocial assessment 31

5.3.1.1 Hot flushes and night sweats assessment 31
5.3.1.2 Quality of life 31
5.3.1.3 Anxiety and depression 31
5.3.1.4 Sleep 31
5.3.3 Process Evaluation 32
5.3.4 Health Economic Assessment 33
5.3.5 Patient Acceptability Assessment 33

5.4 FOLLOW UP 33

5.5 TREATMENT/ASSESSMENT WINDOW 34

5.6 DEVIATIONS AND SERIOUS BREACHES 34

5.7 TRIAL DISCONTINUATION 34
5.7.1 Reasons for trial discontinuation 34

5.8 WITHDRAWAL 34

6 SAFETY 36

6.1 DEFINITIONS 36
1.1.1 Exceptions: 36

6.2 CAUSALITY 36

6.3 REPORTING PROCEDURES 37
6.3.1 SAE 37
6.3.3 Reporting Details 37

6.4 SCTU RESPONSIBILITIES FOR SAFETY REPORTING TO REC 38

6.5 REPORTING WINDOWS 38

7 STATISTICS AND DATA ANALYSES 38

7.1 METHOD OF RANDOMISATION 38

7.2 SAMPLE SIZE 38

7.3 PRIMARY AND SECONDARY ANALYSES 39

7.4 HEALTH ECONOMIC ANALYSES 39

7.5 QUALITATIVE ANALYSIS OF PROCESS EVALUATION 40

8 ETHICAL CONSIDERATIONS 41

8.1 ETHICAL APPROVAL 41

8.2 INFORMED CONSENT PROCESS 41

8.3 CONFIDENTIALITY 41

9 SPONSOR 43

9.1 INDEMNITY 43

9.2 FUNDING 43
9.2.1 Site payments 43
9.2.2 Participant payments 43

9.3 AUDITS AND INSPECTIONS 43

10 TRIAL OVERSIGHT GROUPS 44

10.1 TRIAL MANAGEMENT GROUP (TMG) 44

10.2 TRIAL STEERING COMMITTEE (TSC) 44

10.3 DATA MONITORING AND ETHICS COMMITTEE (DMEC) 44

11 DATA MANAGEMENT 45

11.1 DATA SHARING REQUESTS FOR RESULTS THAT ARE AVAILABLE IN THE PUBLIC DOMAIN 46

12 MONITORING 47

12.1 CENTRAL MONITORING 47

12.2 CLINICAL SITE MONITORING 47

13 RECORD RETENTION AND ARCHIVING 47

14 PUBLICATION POLICY 47

15 APPENDICES 48

15.1 SWAT PROTOCOL 48

16 REFERENCES 50

LIST OF ABBREVIATIONS

AE	Adverse Event
ADT	Androgen Deprivation Therapy
BAUN	British Association of Urology Nurses
CBT	Cognitive Behavioural Therapy
CEACs	Cost-Effectiveness Acceptability Curves
CI	Chief Investigator
CNS	Clinical Nurse Specialist
CONSORT	Consolidated Standards of Reporting Trials
CRF	Case Report Form
CTCAE	Common Terminology Criteria for Adverse Events
DMEC	Data Monitoring and Ethics Committee
DMP	Data Management Plan
eCRF	Electronic Case Report Form
EORTC QLQ-C30	European Organization for Research and Treatment of Cancer Quality of Life Questionnaire – Core
GAD7	Generalised Anxiety Disorder Questionnaire
GCP	Good Clinical Practice
GP	General Practice
HFNS	Hot Flush and Night Sweats
HRA	Health Research Authority
ICC	Intra-Class Correlation
ICER	Incremental Cost-Effectiveness Ratio
ICF	Informed Consent Form
IDMC	Independent Data Monitoring Committee
ISF	Investigator Site File
LHRH	Luteinising Hormone-Releasing Hormone
MHRA	Medicines and Healthcare Products Regulatory Agency
NCI	National Cancer Institute
PCaSO	Prostate Cancer Support Organisation
PCUK	Prostate Cancer UK
PHQ9	Patient Health Questionnaire-9
PI	Principal Investigator
PIC	Participant Identification Centres
PID	Patient Identifiable Data
PIS	Participant Information Sheet
PSQI	Pittsburgh Sleep Quality Index
QALY	Quality-Adjusted Life Years

QoL	Quality of Life
R&D	Research and Development
RCT	Randomised Controlled Trial
REC	Research Ethics Committee
RN	Research Nurse
SAE	Serious Adverse Event
SAP	Statistical Analysis Plan
SCTU	Southampton Clinical Trials Unit
TAU	Treatment as Usual
TM	Trial Manager
TMF	Trial Master File
TMG	Trial Management Group
TSC	Trial Steering Committee
UHS	University Hospital Southampton NHS Foundation Trust
WSAS	Work and Social Adjustment Scale

KEYWORDS

Cognitive behavioural therapy; hot flush; hot flash; night sweat; prostate cancer; quality of life; androgen deprivation therapy

TRIAL SYNOPSIS

**Short title/Acronym:**	MANCAN2
**Full title:**	A multicentre randomised controlled trial of virtual self-help cognitive behavioural therapy to MANage the impact of hot flush and night sweat symptoms in patients with prostate CANcer undergoing androgen deprivation therapy (MANCAN2)

**Trial Phase:**	III (Complex intervention)
Population:	**Inclusion Criteria:**<br>1. A diagnosis of prostate cancer<br>2. Localised or advanced disease stage. Patients may have had potentially curative treatments including, but not limited to, radiotherapy, brachytherapy or surgery.<br>3. Currently receiving Androgen Deprivation Therapy (ADT), and anticipated to require a minimum of 6 months further continuous treatment at the point of registration into the trial. Treatment may have been planned for either a fixed duration (for example, but not limited to, 2 years after radiotherapy) or permanent. Treatment may be with either adjuvant (following potentially curative treatment) or palliative intent. Luteinising Hormone-Releasing Hormone (LHRH) analogues, LHRH antagonists and surgical castration are all acceptable forms of androgen deprivation. Androgen receptor antagonists, including but not limited to, bicalutamide, enzalutamide, apalutamide or darolutamide, or abiraterone, may be given in combination with androgen deprivation according to local practice.<br>4. Presence of problematic Hot Flushes and Night Sweats (HFNS) symptoms defined as a HFNS Rating Scale score of two or more.<br>5. Ability to read and understand English without assistance<br>6. 16 years or older<br>7. Ability to attend virtual group workshops through video conferencing software. If this is not feasible, participants must be able to participate in one-to-one workshops by telephone<br>**Exclusion Criteria:**<br>1. Currently with uncontrolled biochemical, radiological or clinical disease progression or relapse if this would be anticipated to interfere with trial participation as determined by the local principal investigator or co investigator<br>2. Currently receiving chemotherapy. Prior chemotherapy must have been completed with a minimum of 4 weeks elapsed between the date of the

	final dose and confirmation of eligibility. Concomitant use of bone health agents, including zoledronate and denosumab is allowed<br>3. Currently receiving radical multi-fraction external beam radiotherapy or brachytherapy These must have been completed with a minimum of 4 weeks elapsed between the date of the final fraction/treatment and confirmation of eligibility. Single fraction radiotherapy to sites of painful bony metastatic disease or 'STAMPEDE style' palliative prostate radiotherapy is allowed<br>4. Intention to receive ADT on an intermittent schedule<br>5. Use of experimental drugs within other interventional clinical trials. Co-recruitment to observational studies, or studies of surgery or focal ablation techniques where the interventional component is complete, is acceptable<br>6. Currently receiving androgen deprivation as a neoadjuvant treatment<br>7. Medical or psychiatric conditions or other factors that, in the view of the local PI, are likely to impact on the ability of the patient to participate in the trial procedures and interventions
**Primary Objective:**	To determine whether the addition (to Treatment As Usual (TAU) of virtual self-help Cognitive Behavioural Therapy (CBT), delivered by a patient's existing prostate cancer Clinical Nurse Specialist (CNS) team, reduces the impact of HFNS at 6 months post randomisation in men with prostate cancer undergoing ADT.
**Secondary Objectives:**	To determine:<br>1. The effect of the intervention on the impact of HFNS at 6 weeks post randomisation<br>2. The effect of the intervention on HFNS frequency<br>3. The effect of the intervention on men's HFNS beliefs and behaviours<br>4. The effect of the intervention on quality of life (QoL)<br>5. The effect of the intervention on other symptoms including anxiety, depression, mood and sleep<br>6. The effect of the intervention on men's compliance with ADT<br>7. The level of fidelity of the CBT when delivered by a patient's existing prostate cancer clinical nurse specialist (CNS) team<br>8. Resource use analyses<br>9. Prostate cancer CNS team experiences of introducing this new intervention<br>10. Participant acceptability of the intervention<br>11. Explore barriers and facilitators to implementing the intervention into routine practice<br>12. Health economics of the intervention
**Rationale:**	Androgen deprivation therapy (ADT) is a hormonal treatment for prostate cancer, which about half of patients receive at

	some point. Although ADT is an effective treatment, up to 80% of men suffer from hot flush and night sweats (HFNS) which may impact quality of life and potentially treatment compliance. Unfortunately, there are limited effective treatment options for men with HFNS. Prior supporting data suggest that CBT may be able to reduce HFNS in this setting. MANCAN2 will test a virtual self-help CBT intervention, delivered by a patient's existing prostate cancer clinical nurse specialist (CNS) team.
**Trial Design:**	A multicentre randomised controlled trial of the addition of a virtual self-help CBT intervention to TAU versus TAU alone.
**Sample Size:**	Between 144 and 192 patients
**Treatment/Intervention:**	A 4-week self-help treatment schedule with pre and post intervention virtual group workshops, delivered by the prostate cancer CNS team at week 1 and week 4. The intervention content comprises an instructional booklet including information and exercises addressing stress management, paced breathing and cognitive/behavioural strategies to improve wellbeing and for managing hot flushes, night sweats and sleep, and a CD demonstrating relaxation exercises.

**URL for Database:**	SCTU.MDSOL.COM

TRIAL SCHEMA

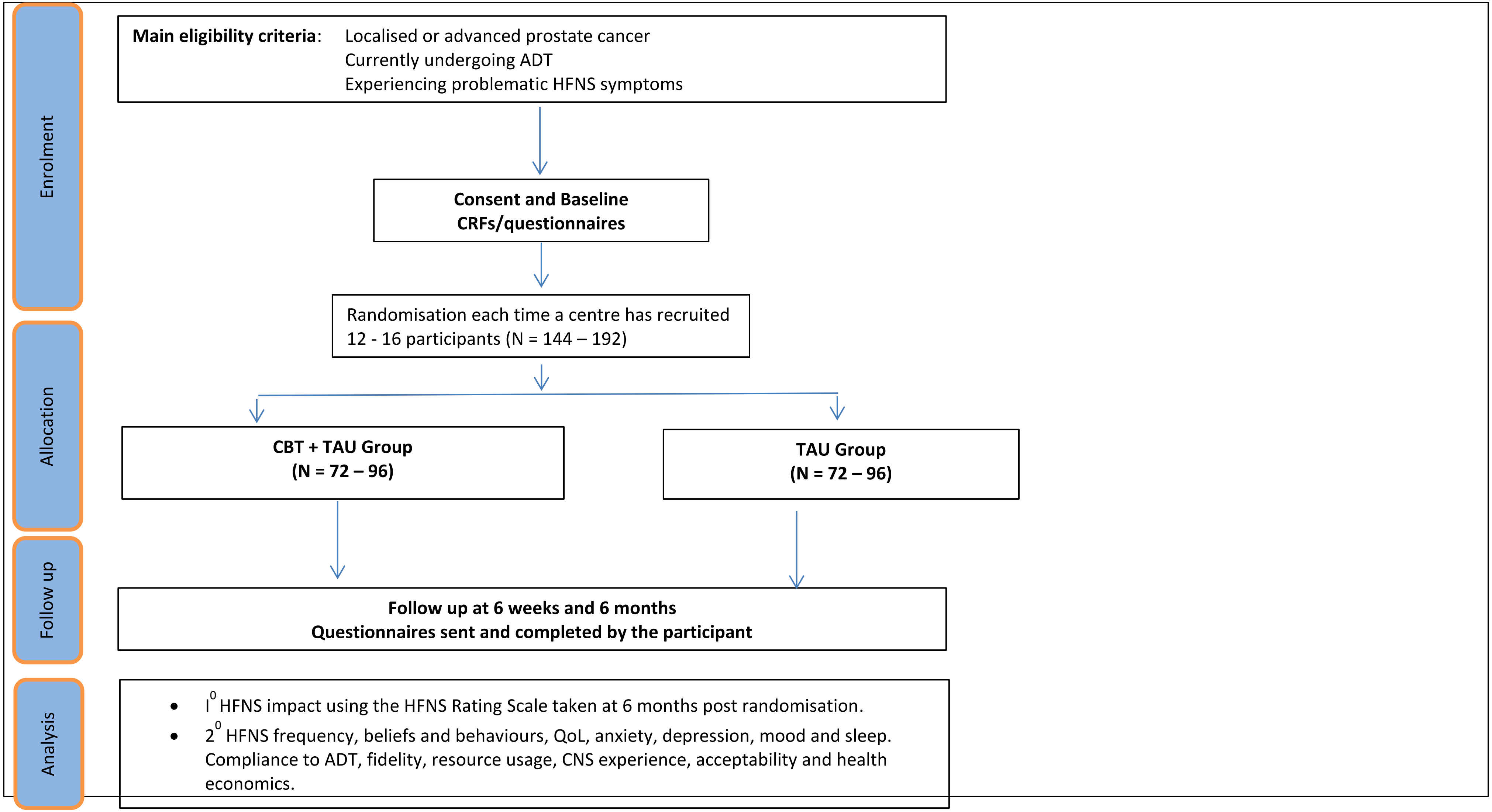

TABLE 1- SCHEDULE OF OBSERVATIONS AND PROCEDURES

Domain / data type	CRF/Assessment questionnaire	Assessor/person completing form	12-16 Participants		Week					
			Screening	Baseline & Randomise	Intervention delivery				Follow up	
					1	2	3	4	6	26
**Window**									+/- 2 weeks	+/- 4 weeks
Reply slip / Contact details	Letter of invitation / Contact information form	Participant	X							
Eligibility screening	Baseline Checklist CRF	Research Nurse (RN)	X							
Route of recruitment	Screening log	RN	X							
Reason not interested	Screening log	RN	X							
Re-Eligibility Screening Check	Baseline Checklist CRF	RN		X						
Demographics[1]	Baseline CRF	Participant		X						
Clinical information[1]	Baseline CRF	RN/ Participant	x							
Consent	Consent form	Participant/RN		X						
Intervention allocation	Randomisation CRF	CTU/Statistician		X						
Hot flushes and night sweats	- HFNS Rating Scale (and HFNS Subscale)<br><br>- HFNS Belief and Behaviour Scale	Participant		X					X	X

Domain / data type	CRF/Assessment questionnaire	Assessor/person completing form	12-16 Participants		Week					
			Screening	Baseline & Randomise	Intervention delivery				Follow up	
					1	2	3	4	6	26
**Window**									+/- 2 weeks	+/- 4 weeks
Health-related QoL	- EORTC QLQ-C30	Participant		X					X	X
Anxiety, depression and mood	- GAD7<br>- PHQ9<br>- WSAS	Participant		X					X	X
Sleep	PSQI (item 6 only)	Participant		X					X	X
Compliance to ADT	ADT Compliance Questionnaire	Participant		X					X	X
CNS training	CNS Training Evaluation Questionnaire	CNS	X							
Attendance	Attendance log	CNS			X	X	X	X		
Reflections and questions post CBT group workshops Number of length of supervision sessions	Supervision notes	CNS			X	X	X	X		
Fidelity: pre and post intervention workshop delivery	Audio recordings of virtual pre/post group workshops	CNS to record all pre/ post group workshops			X			X		
Adherence: Self-Guided CBT Booklet	Patient Evaluation Questionnaire	Participant (intervention arm only)						X		

Domain / data type	CRF/Assessment questionnaire	Assessor/person completing form	12-16 Participants		Week					
			Screening	Baseline & Randomise	Intervention delivery				Follow up	
					1	2	3	4	6	26
**Window**									+/- 2 weeks	+/- 4 weeks
engagement & Lifestyle changes										
Fidelity: pre and post intervention workshop delivery	Fidelity Checklist	Independent person			2 recordings per site randomly allocated					
Resource usage	Participant Resource use Questionnaire	Participant							X	X
Management of HFNS	Participant resource use Questionnaire	Participant							X	X
Clinic costs	Nurse logs	CNS		X	X	X	X	X		
Nurse time	Nurse logs	CNS		X	X	X	X	X		
Training costs	Nurse logs	CNS		X						
QoL	- EORTC QLQ-C30<br>- EORTC QLQ-PR-25	Participant		X					X	X
Evaluation of intervention	Patient Evaluation Questionnaire	Participant (intervention arm only)						X		
Experiences of participating in the CBT group	(Optional) Participant interviews.	Participant (Intervention Arm only) / SCTU Research Team						X		

Domain / data type	CRF/Assessment questionnaire	Assessor/person completing form	12-16 Participants		Week					
			Screening	Baseline & Randomise	Intervention delivery				Follow up	
					1	2	3	4	6	26
**Window**									**+/- 2 weeks**	**+/- 4 weeks**
Implementation and impact on current services	NoMad Questionnaire/ CNS interviews	CNS Team member (x2)	X							X
Implementation of program	Managers interviews	Managers/ SCTU Research Team								X
	Medical Staff interviews	Medical Staff/ SCTU Research Team								X
SAE Collection									X	X

[1] See baseline section 5.1 for the breakdown of Demographic and Clinical data to be collected from participants.

1 INTRODUCTION

1.1 BACKGROUND

Prostate cancer is the commonest cancer in UK men, with a rising incidence now reaching around 48,000 per year.[1] Survival rates are improving with 85% now living at least five years from diagnosis.[2] It is therefore critical to address the impact of cancer treatment on the increasing numbers who will live with, and beyond, a prostate cancer diagnosis.

Prostate cancer biology is critically dependant on cellular signalling through the androgen receptor at all stages of the disease. ADT is a hormonal treatment used to inhibit prostate cancer through the reduction of systemic levels of male androgenic hormones, including testosterone. ADT is used in two main contexts. Firstly, to reduce relapse rates after a course of radical (curative) radiotherapy when it is typically given as an adjuvant treatment for around two years. Secondly as palliative life extending treatment for advanced metastatic disease when it is typically used permanently until end of life and where the median duration of therapy is 4-6 years.[3, 4]

Most ADT in the UK is delivered as monthly, 3 monthly or 6 monthly depot injections of an LHRH analogue. This is usually administered by a General Practice (GP) nurse. A minority receive LHRH antagonists or undergo surgical castration as alternative approaches. (For the purposes of this trial all of these forms of ADT are relevant to our proposed cohort for recruitment.) Overall, approximately 50% of prostate cancer patients will receive ADT at some point during their treatment pathway.[5]

Although ADT is an effective treatment, with virtually all men with advanced disease showing initial clinical response, it is associated with troublesome side effects. Up to 80% of men undergoing ADT suffer from HFNS, in addition to other potential side effects that can include reduced libido, sexual dysfunction, fatigue, gynaecomastia, weight gain and metabolic disturbance, osteoporosis and mood disturbance.[6] Nearly half of patients experience HFNS for up to five years following ADT, even if discontinued. HFNS can be both frequent and severe and lead to a significant decrease in quality of life.[7] They may also cause anxiety, low mood and sleep disturbances.[8] HFNS can occasionally be so severe and debilitating that patients become prepared to stop ADT altogether despite the increased risk of disease relapse (for adjuvant treatment) or reduced survival (for palliative treatment).[9]

Unfortunately, there are few validated safe and effective treatments for men with HFNS.[10] Management of HFNS in prostate cancer is under researched compared to breast cancer.[11] Furthermore, there is evidence suggesting that HFNS tend to be more frequent and severe in men compared to those experienced by women.[12]

In MANCAN2, we propose to build on our prior work (particularly MENOS4 [13] and MANCAN1 [14], described below) which has developed management strategies for HFNS. MANCAN2 will test a 4-week self-help CBT intervention with virtual pre and post intervention group workshops , delivered by the existing prostate cancer CNS team, to men with prostate cancer in a multicentre study.

1.2 RATIONALE AND RISK BENEFITS FOR CURRENT TRIAL

A recent systematic review to determine best practice for managing ADT induced HFNS identified 15 studies. Eight were of pharmacologic interventions and the remainder for complementary and alternative medicine. It concluded that evidence is insufficient to support even the limited current intervention options.[15] Steroidal progestins (e.g., medroxyprogesterone, cyproterone) showed some benefit at reducing HFNS but were associated with side-effects that were not well tolerated including nausea, weight gain, muscle spasms,

depression, insomnia and headaches.[16] Anticonvulsants (gabapentin) and an alpha-agonist antihypertensive (clonidine) did not appear to improve HFNS.[15] Acupuncture demonstrated potential benefit in reducing HFNS and did so without side effects. This was however, based on data from a handful of small studies limiting interpretation (the largest, an RCT in only 31 patients). Acupuncture is not currently routinely available within the NHS for this indication further limiting the practical utility for most patients.

Current UK practice for management of ADT induced HFNS is rather inconsistent, reflecting the lack of positive and methodologically sound data on which to make recommendations. It lags behind breast cancer in this regard. The current NICE guideline NG131 (Prostate cancer: diagnosis and management, May 2019, https://www.nice.org.uk/guidance/NG131) provides recommendations to 'offer' medroxyprogesterone to manage troublesome hot flushes caused by ADT and to 'consider' cyproterone if medroxyprogesterone is not effective or tolerated. The guideline also recommends to ‘Tell people that there is no good-quality evidence for the use of complementary therapies to treat troublesome hot flushes’. There is therefore a pressing unmet clinical need, in a large number of prostate cancer patients, for development of effective, acceptable and ideally side effect free HFNS treatments.

We have developed a theoretical model of HFNS identifying factors that can moderate the intensity and experience of HFNS, such as certain triggers (e.g., hot foods), stress, and cognitive/behavioural responses.[17] The model was based on existing evidence that stressors can increase HFNS reporting [18] and anxiety [19] whereas unhelpful beliefs/cognitions (associated with embarrassment, social anxiety, feeling out of control and an inability to cope) are associated with more problematic HFNS and sleep problems.[20]

We have developed a CBT intervention which has been found to be safe and effective in reducing the impact of HFNS and improving psychosocial functioning for menopausal women [21] and breast cancer survivors.[22, 23] Its effectiveness has been demonstrated when delivered in groups [21, 23], guided self-help formats [21-23], over the telephone [24] and online [25], with minimal or without therapist input.[14, 25]

The CBT intervention was also adapted for men undergoing prostate cancer treatments in the MANCAN1 trial, informed by an earlier qualitative study with prostate cancer survivors who provided feedback on the intervention content as well as the level of guidance required.[14, 26] MANCAN1 was a single centre RCT of 68 patients which compared guided self-help CBT to TAU. Findings showed that guided self-help CBT delivered by a clinical psychologist was a safe and effective way of reducing HFNS symptoms. MANCAN1 showed that self-help CBT significantly reduced the impact of HFNS at 6 weeks post randomisation (primary endpoint, adjusted mean difference: -1.33, 95% CI -2.07 to -0.58; p=0.001).[14] Improvements were maintained at 32 weeks (a secondary endpoint) although at this later time point group differences did not retain statistical significance. There were also significant reductions in negative HFNS beliefs and behaviours, but not in mood or quality of life. We conducted a qualitative study with men who participated in the intervention; men reported that the self-help format was acceptable and that the intervention helped them to undertake positive lifestyle changes.[27] We have also developed and validated the HFNS Beliefs and Behaviour Scale for Men assessing men’s beliefs and behaviours in relation to their HFNS.[28]

Our previous study MENOS4 in women with breast cancer, demonstrated that breast cancer nurse delivered group CBT was both an effective and safe intervention for reducing HFNS symptoms. This multicentre RCT of 130 patients compared group CBT to TAU. The study showed a 46% (6.9-3.7) reduction in the mean HFNS problem rating score at 26 weeks post randomisation in the CBT arm compared to a 15% (6.5-5.5) reduction in the TAU arm (adjusted mean difference -1.96, 95% CI -3.68 to -0.23, p=0.039).[13] There was also an improvement in numerous secondary outcomes. We found a reduction in HFNS frequency of 28% (58 to 42) in the CBT group compared to an 11% (63 to 56) reduction in the usual care group at 26 weeks (adjusted median difference -20.22, 95% CI -34.46 to -4.93, p=0.010) and significant improvements in sleep quality (adjusted mean difference at 26 weeks -0.57, 95% CI -0.81 to -0.33; p<0.0001), anxiety (adjusted median

difference at 26 weeks 2.14, CI -3.61 to -0.66; p<0.005) and depression (adjusted median difference at 26 weeks -2.86, 95% CI -4.73 to 0.98; p<0.003).[13] Breast cancer nurses from 6 cancer centres were trained to deliver the manualised CBT intervention, delivered in weekly group CBT sessions of 90 minutes for six weeks. Fidelity was tested by recording the sessions and rated by an independent psychologist for adherence to the treatment manual. The study showed that a high degree of fidelity was maintained.

We have shown that specialist nursing teams are an effective means to supporting self-management in the context of cancer survivorship, including for men with prostate cancer. As such, there is potential to incorporate this intervention (if found effective) into currently existing staffing structures designed to support men living with and beyond cancer.[29] Stratified pathways of care are being rolled out nationally, with prostate as one of the designated target groups (https://www.england.nhs.uk/improvement-hub/wp-content/uploads/sites/44/2017/11/Stratified-Pathways-of-Care.pdf; https://www.england.nhs.uk/wp-content/uploads/2016/04/stratified-pathways-update.pdf). These national pathways have emphasised the importance of promoting implementation of mechanisms to support patient self-management (including for iatrogenic symptoms like HFNS) through the introduction of patient workshops. An increasing number of services are now developing capacity to deliver such workshops. The intervention proposed in this trial could be introduced through this route.

The global COVID-19 pandemic has also driven an increasing need to develop flexibility in administration of health care interventions and, where feasible, in a remote manner. MANCAN2 will address this need by the delivery of CBT through virtual group workshops using video conferencing software. The prostate cancer CNS team will provide assistance to participants that are unfamiliar with video conferencing software. If participants are not able/willing to use video conferencing software they will be offered one-to-one telephone workshops instead.

MANCAN2 will advance the program of work described above by determining whether ADT induced HFNS in men with prostate cancer can be impacted by a virtual CBT intervention delivered by the existing prostate cancer CNS team within a multicentre study. The emphasis on this existing team, if successful, should facilitate translation through to implementation in routine practice.

2 TRIAL OBJECTIVES

	OBJECTIVE	ENDPOINT USED TO EVALUATE
Primary:	To determine whether the addition (to treatment as usual (TAU)) of a 4-week self-help CBT intervention with virtual pre and post intervention group workshops, delivered by a patient's existing prostate cancer clinical nurse specialist (CNS) team, reduces the impact of HFNS at 6 months post randomisation in men with prostate cancer undergoing ADT.	HFNS Rating Scale at 6 months compared to baseline
Secondary:	1. The effect of the intervention on the impact of HFNS at 6 weeks post randomisation.	HFNS Rating Scale at 6 weeks compared to baseline
	2. The effect of the intervention on HFNS frequency	A subscale of the HFNS Rating Scale, at 6 weeks and 6 months, compared to baseline
	3. The effect of the intervention on men's HFNS beliefs and behaviours	HFNS Beliefs and Behaviour Scale, at 6 weeks and 6 months, compared to baseline
	4. The effect of the intervention on QoL	- EORTC QLQ-C30, at 6 weeks and 6 months, compared to baseline<br>- EORTC QLQ-PR-25, at 6 weeks and 6 months, compared to baseline
	5. The effect of the intervention on other symptoms including anxiety, depression, mood and sleep	Anxiety, depression and mood:<br>- Generalised Anxiety Disorder Questionnaire (GAD7), at 6 weeks and 6 months, compared to baseline<br>- Patient Health Questionnaire-9 (PHQ9), at 6 weeks and 6 months, compared to baseline<br>- Work and Social Adjustment Scale (WSAS), at 6 weeks and 6 months, compared to baseline<br><br>Sleep:<br>- Pittsburgh Sleep Quality Index (PSQI, item 6), at 6 weeks and 6 months, compared to baseline
	6. The effect of the intervention on men's compliance to ADT	Percentage of men compliant with the planned duration of ADT at 6 weeks and 6 months
	7. The level fidelity of CBT when delivered by the prostate cancer CNS team	- Audio recordings of the virtual (pre- and post) intervention group workshops. An independent person will rate a random selection of these for adherence to the treatment manual.

		-Workshop Attendance logs (completed by CNS) will measure patient compliance to the virtual pre and post group intervention workshops .<br>- Post-intervention questionnaire completed by patients will measure how much of the booklet/CD patients engaged with and what lifestyle changes they have made.
	8. Resource usage analyses	-CNS team logs to record staff training cost, time to deliver intervention in a virtual capacity.<br>-Participant resource use questionnaire
	9. Prostate cancer CNS team experiences of delivering this new service	Interviews with prostate cancer CNS team members
	10. Participants' acceptability of the intervention	Interviews with participants
	11. Explore barriers and facilitators to implementing the intervention into routine practice	Interviews with CNS team, medic and manager
	12. Health Economics of the intervention	-Measurement of quality-adjusted life years (QALY)<br>-Collection of data on expected service use<br>-Collection of data pertaining to the cost of the intervention

3 TRIAL DESIGN

This will be a mixed-method study:

(i) A randomised controlled trial

(ii) A process evaluation

3.1 RANDOMISED CONTROLLED TRIAL

3.1.1 Design

A multicentre individually randomised controlled trial of a prostate cancer CNS team delivering a 4-week self-help CBT intervention with virtual pre and post intervention group workshops plus TAU versus TAU alone.

3.1.2 Settings

Approximately 7 UK NHS sites which host a prostate cancer multi-disciplinary team. If required, additional sites (including PIC sites) will be approached and invited to participate. Site selection will give consideration to ensure ethnic, geographic and NHS setting diversity to optimise generalisability of results.

3.1.3 Target population

MANCAN2 will recruit approximately 150 (144-192) men with prostate cancer currently receiving ADT and experiencing problematic HFNS symptoms (defined as a HFNS rating scale score of two or more). We will include patients receiving ADT in both the localised (curative) and advanced (palliative) settings with stratification for disease status. To minimise risk that patients might be symptomatic from their cancer, such that data integrity and outcomes might be influenced, we will recruit advanced disease patients only with hormone sensitive metastatic prostate cancer (excluding later stage castrate resistant disease).

3.1.4 Intervention being assessed

The trial aims to evaluate the effectiveness of the addition of a 4-week self-help CBT intervention with virtual pre and post intervention workshops delivered by the prostate cancer CNS team to TAU alone. The intervention content comprises an instructional booklet (electronic and paper copies available) and a guided relaxation audio (downloadable audio/ CD, see Section 5.2).

3.1.5 Trial outcome measures

The primary outcome measure will be the HFNS Rating Scale taken at 6 months post randomisation.[30] This measure is rated on a 10-point Likert scale where higher scores indicate the patient views their HFNS as more bothersome and interfering with life. The scale is the mean of the following three items:

1. ‘To what extent do you regard your HFNS as a problem?’
2. ‘How distressed do you feel about your HFNS?’
3. ‘How much do your HFNS interfere with your daily routine?’

The HFNS Rating Scale has good internal consistency (Cronbach alpha=0.9) and test-retest reliability (r=0.8). The HFNS Rating Scale will be recorded at baseline, 6 weeks and 6 months. Patients will complete their measures by post. Following a non- response, patients will receive a reminder text and will be telephoned by the research team to collect their measures.

A Patient Evaluation questionnaire will be administered to all participants randomised to the intervention arm at the end of the 4-week intervention by mail/email to collect views on the acceptability of, and satisfaction with, the virtual pre- and post-programme workshops. Following a non- response, patients will receive a reminder text and will be telephoned by the research team to collect their views.

The following secondary outcome measures will also be assessed at baseline, 6 weeks and 6 months post randomisation by mail/email.

1. A subscale of the HFNS Rating Scale will be used to measure weekly HFNS frequency. This measure correlated ($r=0.9$, $p<0.0001$) with daily diary recordings of HFNS in a previous study. [31]
2. HFNS Belief and Behaviour Scale is a 22-item scale measuring types of beliefs and behaviours of men with HFNS. Originally used for women with menopausal symptoms, it has been modified for men.[28]
3. EORTC-QLQ-C30 is a validated 30-item questionnaire for assessing QoL of patients with cancer.
4. Generalised Anxiety Disorder Questionnaire (GAD7) is a self-administered 7 item patient questionnaire (e.g., feeling nervous, restlessness) as both screening tool and severity measure for generalised anxiety disorder.[33]
5. Patient Health Questionnaire-9 (PHQ9) is a self-administered measure of depression/mood severity and treatment response. Patients are asked how often they were bothered by nine problems (e.g., trouble concentrating, poor appetite) over the previous 2 weeks.[34]
6. Pittsburgh Sleep Quality Index (PSQI) is a 19 item self-rated questionnaire that assesses sleep quality, latency, duration, disturbance and medication use, and daytime dysfunction. These are analysed in seven components and summed to one total score.[35]. For the purpose of this trial, only item 6 of the PSQI will be used.
7. Work and Social Adjustment Scale (WSAS) is a self-reported scale of functional impairment attributable to an identified problem. It has been used to study depression and anxiety. The five-item scale includes ability to work, home management, social leisure activities, private leisure activities and ability to form and maintain close relationships.[36]
8. Patient Evaluation Questionnaires evaluating the virtual pre- and post-programme group workshops will be administered similar to the questionnaires used to evaluate the face-to-face sessions in MENOS1.[23]

3.1.6 Health economic evaluation

The economic analysis will take an NHS perspective to estimate both cost effectiveness (£ per change in Hot Flush Rating Scale) and cost utility (Incremental £/QALY) as well as a budget impact. QALYs will be estimated from EORTC using the SCHARR algorithm (https://www.sheffield.ac.uk/scharr/sections/heds/mvh/cancer).

Costs will include that of the intervention and on any changes in use of NHS services. As the intervention cost might differ between that in the trial and in routine practice, we will take two approaches, one detailing changes in CNS team total time allocations in each arm during the trial, and another collecting data on views of both the CNS team and managers on the extent to which the intervention could be incorporated into standard practice (and under what circumstances such as being included in training and in guidelines). While both these costs will be reported, the latter would be more relevant in estimating budget impact if implemented in routine practice. The cost of changes in service use will be based on a customized resource use patient questionnaire at 6 weeks and 6 months.

Estimates of both cost and QALY increments will be estimated for each patient and incorporated into the planned statistical analyses described above. If a clinically relevant gain in the primary outcome is found to be statistically significant, more detailed economic modelling will be carried out, to explore uncertainty and to extend the time frame beyond that of the trial. This will build on recent cost effectiveness modelling for similar interventions for hot flushes in breast cancer.

3.2 PROCESS EVALUATION

The purpose of the process evaluation is to:

1. Understand CNS team the experiences of introducing and running the intervention.
2. Explore participant acceptability and satisfaction.
3. Establish whether the intervention has potential to become a routine practice service.

To achieve this semi-structured interviews and questionnaires will be conducted with patients, key stakeholders and the team delivering the intervention.

Specifically, staff interviews will help us understand site team attitudes, dynamics, perception of the intervention and ability to integrate it into current work.

Patient interviews will help us understand the impact, barriers and facilitators to taking part in this trial and provide insight of self-help CBT as a sustainable treatment option.

Optional consent will be available for trial participants willing to take part in the process evaluation interview as part of the main informed consent form for MANCAN2. Staff at participating sites will be invited to participate in the process evaluation. Upon approach, staff will be provided with a separate participant information sheet (process evaluation interviews) and an informed consent form.

3.3 CENTRE SELECTION

Each centre will have:

1. Availability of at least two prostate cancer CNS team members who are available to be trained
2. Ability to deliver virtual pre- and post-programme workshops. This requires the centre to have provision for video conferencing software established to run the workshops.

3.4 DEFINITION OF END OF TRIAL

The end of the trial will occur following the collection of data for the 6-month assessment point of the last patient recruited to the trial.

4 SELECTION AND ENROLMENT OF PARTICIPANTS

4.1 PATIENT ENROLLMENT AND CONSENT

Consent to enter this trial will be sought from each participant only after a full explanation has been given, an information leaflet offered and a minimum of 24 hours for consideration. Signed participant consent will be obtained. The right of the participant to refuse to participate without giving reasons will be respected.

After a participant has entered the trial the clinician remains free to provide alternative management options to that specified in the protocol at any stage if they feel it is in the participant's best interest, but the reasons for doing so should be recorded. In these cases the participants will remain within the trial for the purposes of follow-up and data analysis as long as they continue to consent to this. All participants are free to withdraw at any time from the trial without giving reasons and without prejudicing further care.

4.1.1 Participant identification

Potentially eligible patients will be approached by members of the site study team to see if they would be interested in learning about the MANCAN2 trial. This may be done in person during normal clinic visits. Alternatively, sites may contact potentially eligible patients by letter or electronic means where this is an established part of their management (for example if a site has a patient cohort in a remote follow up programme as part of their routine care).

4.1.2 Informed consent procedure

A member of the site trial team will discuss the trial with potential participants, explaining the study and what it means to be involved. This may be conducted face to face, virtually using video conferencing software or over the telephone. Those who wish to proceed will be provided (in person or by post) with a MANCAN2 invite pack containing:

- MANCAN2 Invite Letter
- Screening Questions Form
- Patient Contact Details Form
- Participant Information Sheet (PIS)
- Informed Consent Form (ICF)
- Study Decline Form

Consent may be provided either in person, or remotely during discussion by video conferencing software or telephone with the ICF returned using a freepost envelope provided in their MANCAN2 invite pack. The details of how this has been undertaken should be documented.

Upon completion of the ICF, a copy will be given to the participant, a copy stored in the participant's medical record and the original filed in the site trial file. A copy of the ICF and Patient Contact Details Form will also be sent to the SCTU (via safesend or NHS email account) for central monitoring purposes and data collection follow up purposes.

Where possible, reasons for non-participation will be gathered from those who decline the study. Reasons will be collected by the study team member (if patient approached in clinic). Patients who decline the invite sent via mail will be asked to provide a reason on their study decline form . Reasons for non-participation will be stored in the screening log.

4.1.3 Screening Procedures

Once patients have completed the informed consent process, they will then complete and return their patient contact details form and responses to screening questions (either at the same time as the signed ICF or subsequently) to the study team at site either in person or by using the freepost envelope provided.

Once the above documents have been received by site, the site trial team will confirm eligibility (see section 4.2 and 4.3) by completing the eligibility checklist. The site trial team will continue with recruitment and screening procedures until 16 men have provided written consent and have been confirmed to be eligible for trial participation (or a minimum of 12 men with at least two months of recruitment time elapsed).

Once a group of 12-16 men has been formed, the site trial team will contact all eligible patients and conduct a screening review with each (this can be done either in person, or remotely by video conferencing software or telephone). The purpose of this review is to:

- verbally reconfirm that the patient wishes to proceed
- reconfirm any points relating to eligibility that may conceivably have changed (for example current medication)
- to inform the patient that the randomisation process will occur in the near future

4.1.4 Completion of Baseline Questionnaires

Baseline Questionnaires (containing Demographic questions, HFNS Rating Scale, HFNS Sub Scale, HFNS Belief and Behaviour scale, EORTC-QLQ-C30, , GAD7, PHQ9, WSAS and PSQI item 6) will be provided (in person or via mail) to all eligible men following their screening review. The site research team will complete the clinical Baseline form using patient medical notes and may contact the patient (telephone or face to face) to complete some sections of this form.

Where Baseline Questionnaires have not been returned to the research team within 7-10 days of the site trial team providing or posting out, the site trial team will call the patient and will arrange to collect the measures over the telephone. They may also collect incomplete measures with the patient over the telephone.

The site trial team may use a range of methods to communicate with patients such as video conferencing, emails, text and telephone calls.

Randomisation will be undertaken (see section 4.5) once all (12-16) men have completed their Baseline Questionnaires.

For further information on recruitment, screening and baseline procedures please refer to the MANCAN2 Patient Flow document.

4.2 INCLUSION CRITERIA

1. A diagnosis of prostate cancer
2. Localised or advanced disease stage. Patients may have had potentially curative treatments including, but not limited to, radiotherapy, brachytherapy or surgery.
3. Currently receiving ADT, and anticipated to require a minimum of 6 months further continuous treatment at the point of registration into the trial. Treatment may have been planned for either a fixed duration (for example, but not limited to, 2 years after radiotherapy) or permanent. Treatment may be with either adjuvant (following potentially curative treatment) or palliative intent. LHRH analogues, LHRH antagonists and surgical castration are all acceptable forms of androgen deprivation. Androgen receptor antagonists, including but not limited to, bicalutamide, enzalutamide, apalutamide or darolutamide, or abiraterone, may be given in combination with androgen deprivation according to local practice.
4. Presence of problematic HFNS symptoms defined as a HFNS Rating Scale score of two or more.
5. Ability to read and understand English without assistance
6. 16 years or older
7. Ability to attend virtual (or face-to-face) group workshops through video conferencing software. If this is not feasible, participants must be able to participate in one-to-one workshops by telephone

4.3 EXCLUSION CRITERIA

1. Currently with uncontrolled biochemical, radiological or clinical disease progression or relapse if this would be anticipated to interfere with trial participation as determined by the local principal investigator or co-investigator
2. Currently receiving chemotherapy. Prior chemotherapy must have been completed with a minimum of 4 weeks elapsed between the date of the final dose and confirmation of eligibility. Concomitant use of bone health agents, including zoledronate and denosumab is allowed
3. Currently receiving radical multi-fraction external beam radiotherapy or brachytherapy These must have been completed with a minimum of 4 weeks elapsed between the date of the final fraction/treatment and confirmation of eligibility. Single fraction radiotherapy to sites of painful bony metastatic disease or 'STAMPEDE style' palliative prostate radiotherapy is allowed
4. Intention to receive ADT on an intermittent schedule
5. Use of experimental drugs within other interventional clinical trials. Co-recruitment to observational studies, or studies of surgery or focal ablation techniques where the interventional component is complete, is acceptable
6. Currently receiving androgen deprivation as a neoadjuvant treatment
7. Medical or psychiatric conditions or other factors that, in the view of the local PI, are likely to impact on the ability of the patient to participate in the trial procedures and interventions

Concomitant Medications and HFNS interventions:
Interventions intended to mitigate HFNS, including but not limited to, medications, herbal remedies, vitamin supplements, yoga and acupuncture are permitted. All other concomitant medications are permitted. Use of HFNS and concomitant medications will be recorded.

4.4 SCREENING FAILURES

Reasons for screening failures will be documented on the screening log, which will be sent to SCTU. The SCTU research team may approach sites and request for sites to provide a summary of the reasons for screen failures so that recruitment can be closely monitored at site.

4.5 REGISTRATION AND RANDOMISATION PROCEDURES

Each centre will aim to run four groups (two CBT + TAU Arms and two TAU Arms) but fewer or more randomisation groups will be permitted per site across the trial as a whole. Each group will have between 6 and 8 men.

We intend to recruit 12-16 men (6-8 per arm) for each round of randomisation per site. Recruitment will continue until ideally 16 men (or a minimum of 12 men with at least two months of recruitment time elapsed):

- Have provided written consent
- Have been confirmed to be eligible for trial participation
- Have completed Baseline Questionnaires

Once this is achieved then the site trial team will send the participant IDs to SCTU and randomisation will be undertaken.

For participants randomised to the intervention arm, a pre and post workshop reminder text or email will be sent to each patient to notify of the two scheduled virtual group workshop dates and times.

Participants will be individually randomised in a 1:1 ratio in groups of 6-8 participants to either the CBT + TAU or TAU alone arms. The RN will be sent the allocation results for all the men at one time point, and they will inform each participant of their group allocation (CBT + TAU or TAU alone). The site will then run the first pre intervention group workshop for the participants randomised to self-help CBT + TAU. Although neither participants nor the prostate cancer CNS team can be blinded to allocation, the research team member collecting the 6-week and 6-month outcome data by telephone will remain blinded.

Shorter (1 to 1) telephone sessions will be offered to men who are unable to attend their scheduled group workshop/s. Similarly, men who drop out of group workshops will be offered these sessions too.

Once Randomisation has taken place, the RN will notify the patients GP of randomisation by sending a GP letter.

5 TRIAL OBSERVATIONS AND PROCEDURES

5.1 BASELINE DATA COLLECTION

Baseline data to be recorded will include:

- Age
- Date of prostate cancer diagnosis
- Localised or advanced metastatic disease
- Curative or palliative treatment intent (yes, no)
- HFNS Rating Scale score
- Duration of this score (approximate, in months)
- Current cancer therapies
    - ADT type (LHRH analogue, LHRH antagonist, surgical castration)
    - Date ADT commenced
    - Planned further duration of ADT (in months or permanent)
    - Planned dates for ADT injection administration (for those on LHRH analogues or LHRH antagonists)
    - Oral hormonal agent (abiraterone, enzalutamide, darolutamide, apalutamide, bicalutamide, other)
    - Bisphosphonate (yes, no)
- Prior prostate cancer therapies (each: yes, no)
    - Prostatectomy, prostate external beam radiotherapy, prostate brachytherapy, prostate focal ablation therapy
- Current treatment for HFNS symptoms (each : yes, no)
    - Medroxyprogesterone acetate, megestrol acetate, cyproterone acetate, gabapentin, clonidine, venlafaxine, paroxetine, acupuncture, hypnotherapy, herbal remedies, homeopathy, other
- Highest educational qualification
- Ethnic group
- Current employment status
- Relationship status
- Smoking
- Drinking
- Exercise participation
- Height
- Weight
- English literacy
- Planned format of CBT intervention (video conferencing or telephone)

5.2 TRIAL PROCEDURES

CBT (+ TAU) ARM

Men in the CBT intervention arm will receive a 4-week self-help treatment schedule. The intervention content comprises an instructional self-help booklet (electronic and paper copies available) including information and exercises addressing stress management, paced breathing and cognitive/behavioural strategies to improve wellbeing and for managing hot flushes, night sweats and sleep, and a downloadable audio/CD demonstrating breathing/relaxation exercises. In MANCAN2, participants will engage in two virtual group workshops, the first will offer practical help on how to use the self-help CBT guide (booklet) appropriately

and provide participants with an opportunity to meet other men experiencing similar symptoms through video conference technology. The second virtual group workshop will focus on practical tips and strategies to help men maintain their gains in the future.

The method of workshop delivery is not limited to virtual delivery. That is, sites can deliver the workshops to the men in a face-to-face group setting, in a private room, at the participant's hospital site. Furthermore, sites can adopt a hybrid approach (a mix of virtual and face-to-face meetings for delivery) when delivering the workshops. Sites must record the method of workshop delivery used for each participant. (Mixed meetings should not be attempted. Thus participants should either all be face to face, or all virtual, for any particular meeting)

5.2.1 *The method of workshop delivery (virtual, face-to- face or hybrid) should be determined by the local Principal Investigator, based on their assessment of what would be optimal. Training*

Virtual training of the prostate cancer CNS team to deliver the virtual pre- and post-group intervention workshops will be conducted by a study clinical psychologist. Knowledge will be assessed at the end of the virtual training session by multiple choice questionnaire. Virtual training will be delivered through video conferencing software.

Prostate Cancer CNS team members will undertake two virtual group training sessions delivered by the study clinical psychologist in groups of 10 maximum. The virtual group training sessions will take place over two days, 6 hours for the first session and 3 hours for the second session. The sessions will cover a manualised and evidence-based approach to managing HFNS symptoms during ADT for prostate cancer[37].
Training will occur as close as possible to initiation of delivery of the CBT intervention to patients. All training sessions will be recorded and all CNS team members will be sent copies of their recorded group training session so that they can access this as refresher training, prior (within 3 weeks, where possible) to the delivery of the first group workshop at site. To enhance efficiency of training sessions, team members will be sent the manual in advance with tasks to complete in preparation, including a sleep diary and listening to the CD.

CNS team members will receive a list of virtual group training slots to book onto. The sessions will include CNS team members from other study sites. It is a requirement for all CNS team members at each site to attend the virtual group training sessions however, provisions may be made for CNS team members that are unable to attend. That is, providing at least one member of the CNS team at site has received the group virtual training, additional CNS team members at that site (who are unable to attend any of the virtual group training sessions) will be sent a copy of the recorded group training session and will review this as part of their training. However, this training option will be at SCTU discretion.

The training session will include the manual, the self-help book and specific training about CBT, as well as prostate cancer specific issues and how to manage groups. The training will provide the background theoretical knowledge and practical skills to facilitate self-help CBT by examining how thinking and behaviour can have a significant impact on men's experience of HFNS during prostate cancer treatment and through helping men to develop strategies to manage them. These include understanding negative emotions and HFNS, managing unhelpful thoughts and behaviour, improving sleep and using paced breathing to manage flushes and night sweats. Training sessions cover:

- Why CBT is used for HFNS: theory and evidence
- Techniques of stress management, managing hot flushes, sleep and night sweats, maintaining changes, and paced breathing

5.2.2 *Supervision*

Prostate cancer CNS team members will receive ongoing supervision of their delivery of the virtual self-help CBT. Supervision will be on a CNS driven basis. CNSs will be instructed that they can contact the study clinical psychologist if they have any issues or concerns or need to ask general questions. They will record reflections and any questions or problems after the virtual pre- and post-programme workshops and will email these to the study clinical psychologist who facilitated their training. Feedback will be given by video conferencing software, telephone or email. Data will be collected on number, length and topics covered during supervisory sessions.

Study training completion will be documented on the training log, which will be sent to SCTU.

5.2.3 *Adherence*

Adherence to self-help CBT will be measured by the number of booklet chapters read and the number of times a participant reports practising relaxation and paced breathing weekly.

5.2.4 *Fidelity*

Patient Evaluation Questionnaires (post-intervention) will be used to determine how much of the booklet/CD patients engaged with and what lifestyle changes they have made. Prostate cancer CNS team members involved in delivering the CBT intervention will undertake virtual training by the study clinical psychologist, using a training manual. Team members will have continuous access to the training slides as well as the training manual to help support treatment fidelity during the trial. Similarly, participants will have access to the self-help CBT guide which will include troubleshooting (frequently asked questions and answers) sections to help with treatment adherence and fidelity.

Fidelity will be tested by recording all pre and post intervention group workshops (with consent obtained from participants), and 20% will be randomly selected (with a computer-generated random number sequence), ensuring two sessions per site. An independent person will rate these for adherence to the treatment manual. Some recordings may be used by the trial team to provide feedback to CNS team members to ensure adherence to the treatment model.

The audio files of the recorded workshops will be sent securely to the research team at SCTU via an nhs.net email account or Dropoff- a UoS secure file transfer system. They will be stored securely on the computer system at the University of Southampton, which only the research team can access. Depending on the video conferencing software used at site, video files of the recorded workshop may be obtained too and sent to SCTU using the same secure channel. Consent for workshops to be recorded and the audio/ video files to be transferred securely to SCTU will be obtained from all participants at the beginning of the trial during Informed Consent Form completion.

The workshop recordings will be destroyed at the end of the study, once fidelity checks have been completed.

TREATMENT AS USUAL (TAU)

All patients, regardless of randomised allocation, will receive TAU for HFNS symptoms as determined by their local care team and institutional practice. TAU will be defined as care consistent with NICE guideline NG131, Prostate cancer: diagnosis and management (https://www.nice.org.uk/guidance/ng131). This includes access to a specialist prostate cancer urologist or oncologist and a named prostate cancer CNS team member, as well as locally and nationally available cancer information. It also allows for options for pharmacotherapy for HFNS which may include, but are not restricted to, those recommended in NG131.

At the end of the study, all participants that randomise into the TAU arm will receive a copy of the CBT Booklet and CD/ Audio files (demonstrating the breathing and relaxation exercises).

5.3 DATA COLLECTION/ASSESSMENT

The schedule for timing, frequency and method of collection of all trial data is summarised in Table 1. Assessments will be performed as close as possible to the required time point. Ideally the commencement of self-help CBT will begin 3 weeks after randomisation. The duration of the intervention and contemporaneous TAU is 4-weeks. Post-intervention measures will be taken in both groups in parallel (i.e. 6 weeks and 6 months post-randomisation).

The Case Report Forms (CRF) should be completed in black ballpoint pen, with unique participant ID number and initials recorded on the header of each individual form. Information entered incorrectly should only be amended on the original CRF prior to photocopying. Corrections should be made by deleting with a single line through the entry and writing the correct value alongside the box; all amendments should be initialled and dated. Once complete, the CRFs will be photocopied and the copy should be sent to SCTU. The originals should be filed in the appropriate site file folder at the site.

5.3.1 Psychosocial assessment

The following outcome measures will be assessed at baseline, week 6 (+/- 2 weeks) and month 6 (+/- 4 weeks). These will be completed by both the intervention and TAU participants.

5.3.1.1 Hot flushes and night sweats assessment

The HFNS Rating Scale measures the extent to which HFNS are problematic, distressing and interfere with daily life.[30] Three items are rated on a 10-point Likert scale where higher scores indicate the patient views their HFNS as more bothersome and interfering with life. A change of ≥1.5 points on this scale is considered clinically relevant.[13, 14] The HFNS Rating Scale also includes a subscale measuring HFNS frequency that asks men to estimate how many flushes/sweats they have had in the past week.
The HFNS Belief and Behaviour scale is a 22-item scale measuring types of beliefs and behaviours of men with HFNS. Subscales include: (i) beliefs about HF in social context (e.g. everyone is looking at me), (ii) beliefs about coping/control of hot flushes (e.g. when I have a HF I think they will never end), and (iii) beliefs about night sweats and sleep (e.g. if I have NS I'll never get back to sleep). HFNS Behaviours include (i) positive coping behaviour, e.g. accepting HFNS, using breathing and calming responses; (ii) avoidance behaviour. Originally used for women with menopausal symptoms, it has been modified for men.[31]

5.3.1.2 Quality of life

QoL will be assessed using the EORTC-QLQ-C30. EORTC-QLQ-C30 is a validated 30-item questionnaire for assessing QoL of patients with cancer. Anxiety and depression
The GAD7 is a self-administered 7 item patient questionnaire (e.g., feeling nervous, restlessness) as both screening tool and severity measure for generalised anxiety disorder.[33]

The PHQ9 is a self-administered measure of depression/mood severity and treatment response. Patients are asked how often they were bothered by nine problems (e.g., trouble concentrating, poor appetite) over the previous 2 weeks.[34]

The WSAS is a self-report scale of functional impairment attributable to an identified problem. It has been used to study depression and anxiety. The five-item scale includes ability to work, home management, social leisure activities, private leisure activities and ability to form and maintain close relationships.[36]

5.3.1.3 Sleep

The PSQI is a 19 item self-rated questionnaire that assesses sleep quality, latency, duration, disturbance and medication use, and daytime dysfunction. These are analysed in seven components and summed to one total score.[35] For the purpose of this trial, only item 6 of the PSQI will be used.

5.3.1.4 ADT Compliance Questionnaire

The questionnaire will ask patients to report whether they have received their ADT as planned.

5.3.2 Process Evaluation

CNS team

To ensure a high-quality delivery, 2 key CNS team members per site will be interviewed as individuals or as a group (interviewee preference) before trial training to discuss topics including pre-intervention practice and perception of the intervention. Where possible, post intervention interviews will take place up to 1 month after the post-intervention group workshops and will explore topics including experiences of setting up and delivering the intervention, any impact on day-to-day working and potential for running the intervention in routine practice.

Additionally, CNS team members will be asked to complete The Normalisation MeAsure Development questionnaire (NoMAD) to aid reflection on NPT constructs and to quantify the perspective of those directly involved in the delivery of the trial.

Patients

Participants will have the option to be contacted by the research team to take part in an interview when completing the informed consent form for the MANCAN2 trial.
Semi-structured interviews will be conducted within 1 month of patient intervention completion. The purpose of the interview is to gain an in-depth understanding of their experiences of participating in the self-help CBT intervention, with attention given to the virtual nature of the pre-and post-intervention workshops and motivation to sustain use of the intervention.

Medical staff

A member of the medical team responsible for prostate care services, who is likely to impact on the adoption of interventions for routine care services, will be identified by the CNS team.
The purpose of this interview is to understand a medics perception of the intervention, the process involved in running the intervention, including engagement with staff and patients, post intervention identification and treatment of men with HFNS and the potential for running the intervention in routine practice.

Manager

A member of management who has influence on the implementation of a new service, will be identified by the CNS team delivering the intervention and invited for interview.
The interview will explore; the process of encouraging CBT take-up in patients, how patients access the intervention, the potential for running the intervention in routine practice and if the intervention fits with organisational priorities.

All interviews will take place via phone or video conference at the interviewee's preference and last approximately 45-60 minutes. Interviews will be recorded, transcribed and pseudonymised for data analysis purposes and managed in accordance with the University of Southampton Research Data Management Policy.

For further information please refer to the MANCAN2 Process evaluation Manual.

5.3.3 *Health Economic Assessment*

The economic analysis will estimate the costs of providing the CBT intervention, and the cost consequences of the intervention for NHS services and for costs borne by patients. However, an economic evaluation will be conducted only if the intervention proves to be effective, as defined by a ≥1.5 point improvement in the HFNS. The cost of the intervention will be reported regardless of its effectiveness. If effective, cost effectiveness will be expressed in cost per unit change in HFNS and per QALY, based on the incremental differences between arms.

A health economic plan will be drawn up and incorporated into a combined Statistical and Health Economic Analysis Plan. The identification and collection of costs will be undertaken using the following methods:

1) NHS
Data on the use of medication, primary care visits, and out-patient visits will be collected using the Resource Use Questionnaire. In addition, we will estimate out-of-pocket spending such as herbal remedies, acupuncture or alternative therapies and time off work due to HFNS. The cost of the intervention will be based on nurse logs to record staff training cost, and time to deliver the intervention. This information will be used for sensitivity analysis from a societal perspective. We will use an adapted form of the Client Service Receipt Inventory.[38] Intervention-specific resource use will be collected through nurse logs to record staff training cost, and time to deliver the intervention.

2) Participants
Data will be collected on all participants about other actions that they may be taking to manage their HFNS, including yoga, acupuncture or other behavioural activities (see section 7.4). Collection of such information from each participant will be through questionnaire sent by mail at 6 weeks and 6 months. QoL will be measured by EORTC-QLQ-C30 and will be collected at baseline, 6 weeks and 6 months.

5.3.4 *Patient Acceptability Assessment*

An evaluation questionnaire will be administered at the end of the 4-week intervention to those participants in the intervention arm.

5.4 FOLLOW UP

All participants will be included in the study from randomisation (time point 0) until 6 months post randomisation (+/- 4 weeks for post intervention data collection: HFNS Rating Scale, HFNS Belief and Behaviour scale, EORTC-QLQ-C30, GAD7, PHQ9, WSAS and PSQI item 6).

All participants will complete their Baseline, 6 week and 6 month questionnaires by paper CRF and will return these via mail to the research team at SCTU (Baseline questionnaires will be posted back to the research team at site), using the freepost envelope provided. All participants will be sent their questionnaires via the post and will be asked to complete and return to the research team at SCTU using the freepost envelope provided. Where questionnaires have not been returned within 10 days of being posted out, participants will be contacted by telephone by a member of the SCTU research team and data may be collected over the telephone.
If requested, one additional questionnaire booklet will be sent out for each time point during the study.

Some of the study questionnaires (NoMAD and the Patient Evaluation Questionnaire) will be sent to the participants via email (using secure nhs.net email or safesend). The forms will be pseudonymised (identified by the participant study ID) and will not contain any personal identifiable information. Participants will complete these questionnaires electronically and then email back their questionnaires using the secure email channel. If requested, patients can be posted out copies to complete at home and return via post to SCTU, using a Freepost envelope.

NB: Participants will also have the option of returning a subset of the questionnaires and completing the remaining questionnaires over the telephone with a researcher. Other methods may be implemented in effort to increase the response rate, including sending patients reminder texts or emails, altering the order of presentation of scales; using coloured paper to highlight different sections and scales in the questionnaire booklet.

The opportunity will be taken to implement a study within a trial (SWAT) to explore whether a theory-based cover letter will improve the 6- month paper questionnaire return rate. The full SWAT protocol can be found in appendix 1.

5.5 TREATMENT/ASSESSMENT WINDOW

A window of ± 2 weeks for the 6 week assessments and +/- 4 weeks for the 6 months assessments for questionnaires, health economics and process evaluation data will be observed.

5.6 DEVIATIONS AND SERIOUS BREACHES

Any trial protocol deviations/violations and breaches of Good Clinical Practice (GCP) occurring at sites should be reported to SCTU and the local Research and Development (R&D) office immediately. SCTU will then advise of and/or undertake any corrective and preventative actions as required. All serious protocol deviations/violations and serious breaches of GCP and/or the trial protocol will immediately be reported to the Health Research Authority (HRA) and Research Ethics Committee (REC).

5.7 TRIAL DISCONTINUATION

In consenting to the study, participants have consented to the trial intervention, follow-up and data collection. Participants may be discontinued from the trial procedures at any time.

5.7.1 Reasons for trial discontinuation

Participants may be discontinued from the trial in the event of clinical decision, as judged by the PI, termination of trial by sponsor and participant choice. Full details of the reason for trial discontinuation should be recorded in the CRF and medical record.

5.8 WITHDRAWAL

The participant is free to withdraw consent from participation in the trial at any time without providing a reason.

Investigators should explain to participants the value of remaining in study follow-up and allowing this data to be used for trial purposes. Where possible, patients who have withdrawn from trial intervention should remain in follow-up as per the trial schedule. If a participant initially consents but subsequently withdraws from the trial, a clear distinction should be made as to what aspect of the trial the participant is withdrawing from. These aspects could be:

1. Withdrawal from trial intervention
2. Withdrawal from further study follow-up
3. Withdrawal from entire study and does not want data to be used.

Details of study discontinuation (date, reason if known) should be recorded in the End of Study paper CRF, completed by the RN (or Trial Manager) depending on which delegate received the request from the patient. If completed by the RN, the form should be emailed to the MANCAN2 Trial Manager (TM). If received by the TM, the TM will complete the End of Study form and notify the PI at site. The RN will notify the patients GP of withdrawal. Any queries relating to potential withdrawal of a participant should be forwarded to the TM immediately via telephone or email.

We will make every effort to reduce loss to follow-up using the methods listed below:

1. We will emphasise the importance of getting follow-up data to all participants at baseline and the different follow-up assessment points.
2. Unless they have explicitly requested otherwise, all participants will be invited to complete follow-up questionnaires.

In addition to the trial, each participant (i.e. staff/patient) has the right to withdraw from the process evaluation at any time and request that any data collected be deleted. It will not be possible for the participant to withdraw their data once the analysis has started because the data collected will already be pseudonymised and have been used. but the participant can inform the research team if they do not want their anonymised interview content to be used in publications.

Participants can withdraw from the process evaluation without giving a reason by contacting the research team.

6 SAFETY

6.1 DEFINITIONS

Adverse Event (AE): any untoward medical occurrence in a participant or clinical trial participant which does not necessarily have a causal relationship with trial treatment or participation. An AE can therefore be any unfavourable and unintended sign (including an abnormal laboratory finding), symptom, or disease temporally associated with the trial intervention or participation (regardless of causality assessments).

Serious Adverse Event (SAE): is any untoward medical occurrence or effect that:

- Results in death
- Is life-threatening*
- Requires hospitalisation**, or prolongation of existing hospitalisation
- Results in persistent or significant disability or incapacity
- Is a congenital anomaly or birth defect
- Other important medical events***

*'life-threatening' in the definition of 'serious' refers to an event in which the patient was at risk of death at the time of the event; it does not refer to an event which hypothetically might have caused death if it were more severe.
**Hospitalisation is defined as an inpatient admission, regardless of length of stay, even if the hospitalisation is a precautionary measure for continued observation. Hospitalisations for a pre-existing condition, including elective procedures that have not worsened, do not constitute an SAE.
***Other important medical events that may not result in death, be life threatening, or require hospitalisation may be considered a serious adverse event/experience when, based upon appropriate medical judgment, they jeopardise the participant and may require medical or surgical intervention to prevent one of the outcomes listed in this definition.

It is the responsibility of the PI or delegate to define an event as 'not serious' (AE) or 'serious' (SAE). All adverse events that fulfil the criteria definition of 'serious', must be reported to SCTU using the SAE report form (see section 6.3.3)

6.1.1 Exceptions:

For the purposes of this trial, the following SAEs **do not** require reporting to SCTU using the Serious Adverse Event Report Form:

- Death due to prostate cancer
- Prostate cancer disease progression
- Hospitalisation for elective treatment of a pre-existing condition

6.2 CAUSALITY

A complete assessment of AE causality must always be assessed by the PI or delegate. If any doubt about the causality exists, the local investigator should inform SCTU who will notify the Chief Investigator (CI). Other clinicians may be asked for advice in these cases. In the case of discrepant views on causality between investigator and others, all parties will discuss the case. In the event that no agreement is made, the REC will be informed of both opinions within the required timelines.

Table 2: Assessment of SAE causality

Relationship	Denoted
Related	Some or clear evidence of causal relationship
Unrelated	No evidence of any causal relationship

In terms of event status; **Not related to treatment** would highlight that the SAE is not related to the trial intervention. **Related and unexpected SAE** would be classified as an SAE which is related to the trial treatment/intervention and is unexpected.

6.3 REPORTING PROCEDURES

6.3.1 SAE

At week 6 and month 6, participants will be asked if they have been hospitalised (stayed over night in hospital as an inpatient) during the course of the trial (this does not include planned treatments or hospital visits for an existing medical condition). The SCTU trial management team will notify the relevant PI at site of any indication of hospitalisation that the patient may have reported on their week 6 and month 6 follow-up CRF and will subsequently ask the PI to complete a 'Serious Adverse Event Report Form – Non-CTIMP'.

6.3.2 Expectedness

For the purposes of this trial no SAEs are to be considered expected.

6.3.3 Reporting Details

A 'Serious Adverse Event Report Form – Non-CTIMP' should be completed for all SAEs and emailed to SCTU within 24 hours of the -Principal Investigator becoming aware of the event. The Principal Investigator should complete the SAE form, email a scanned copy of the form with as much detail as possible to the SCTU together with anonymised relevant treatment forms and investigation reports.

Alternatively, SCTU can be contacted by telephone to report the event and then emailed a scanned copy of the SAE report form completed as above as soon as possible.

SAE REPORTING CONTACT DETAILS

Please email a copy of the SAE form to

SCTU within 24 hours of becoming aware of the event

Email: ctu@soton.ac.uk

FAO: Quality and Regulatory Team

For further assistance: Tel: 023 8120 4138 (Mon to Fri 09:00 – 17:00)

The responsible investigator (or delegate) should assign the seriousness, causality and expectedness of the event. The event term should be the most appropriate medical term of concept and grades given in accordance with the National Cancer Institute Common Terminology Criteria for Adverse Events version 5 (NCI CTCAE v5). Additional information should be provided as soon as possible if the event has not resolved at the time of reporting.

6.4 SCTU RESPONSIBILITIES FOR SAFETY REPORTING TO REC

The SCTU will notify the REC of all **Related and Unexpected** SAEs occurring during the study within 15 days of the report receipt. The SCTU submit all safety information to the REC in an Annual Progress Report.

6.5 REPORTING WINDOWS

All SAEs must be reported from the date of consent until end of study. For SAE reporting purposes, end of study is defined as 26 weeks post start date of study intervention/ control (treatment as usual).

See below for SAE reporting windows:

- **Between informed consent and start date of study intervention/ control:** All events that are considered SAEs that are deemed by the investigator to be related to trial procedures
- **From the start date of the study intervention/ control until 26 weeks post start date:** All events are considered to be SAEs

7 STATISTICS AND DATA ANALYSES

7.1 METHOD OF RANDOMISATION

Once 12-16 men from a site are consented and recruited to the study, their details will be passed to SCTU for randomisation. We are aiming to recruit 16 men (8 per group). However, if having recruited for a period of two months, at least 12 men (6 per group) have been recruited, then randomisation will take place at this time. A computer-generated randomisation sequence will be created, allocating participants in a one-to-one ratio, stratified, with fixed block size, by

- Centre
- Disease status (curative versus palliative)

This process will be repeated for each cohort group so that allocation does not affect the allocation sequence of subsequent cohorts.

7.2 SAMPLE SIZE

We regard a ≥1.5 point difference in HFNS problem rating as clinically relevant. Based on similar interventions, this effect size is also considered realistic [4, 5]. To detect a ≥1.5 point difference in mean HFNS Problem Rating between CBT + TAU versus TAU alone at 6 months post-randomisation, with a standard deviation of 2.21 (32), 90% power and 5% type 1 error rate, requires a sample size of 94. The sample must also account for clustering introduced by the intervention (those attending the same CBT workshop may have more similar outcomes than those outside the group). Assuming 8 participants per group in the intervention, and intra-class correlation (ICC) of 0.01, requires 111 participants. An ICC of 0.01 is thought appropriate given the intervention is largely by self-management and smaller ICCs have been observed in similar studies [4]. The sample size increases to 150 (75/arm) allowing for 26% loss to follow-up (as per MENOS4).

A sample size of 150 corresponds to each site running four groups (2 per randomised arm), ensuring that a comprehensive process evaluation can be conducted. If each site recruits 4 patient groups, then each site

will recruit between 24 (6 per group) and 32 (8 per group) patients. Across 6 sites this leads to between 144 and 196 participants. An additional 7th site provides leeway with regards to reaching our recruitment target.

7.3 PRIMARY AND SECONDARY ANALYSES

The primary outcome measure, HFNS Rating Scale compared to baseline, will be compared between the CBT intervention + TAU arms at 6 months post randomisation. Difference in HFNS Rating Scale between the two groups will be analysed using a linear mixed model, adjusting for baseline HFNS Rating Scale and stratification factors. Therapy group will be included in the model as a random effect. Interpretation of the effect of the intervention will be based on the regression coefficient for group and corresponding 95% confidence interval. Secondary outcomes at 6 weeks and 6 months post-randomisation will be analysed similarly, accounting for stratification factors and clustering.

Analyses will be based on a modified intention-to-treat sample (i.e. excluding participants not contributing data). This will be supported by an analysis that deals with missing data should missingness lead to >10% of the sample being excluded. Methods may involve either multiple imputation using chained equations, or full information maximum likelihood.

MANCAN2 will be analysed according to the principles of the International Conference on Harmonisation E9 guidelines and reported using Consolidated Standards of Reporting Trials (CONSORT). A full statistical analysis plan (SAP) will be developed prior to the final analysis.

7.4 HEALTH ECONOMIC ANALYSES

The economic analysis will take an NHS perspective to estimate both cost effectiveness (£ per change in HFRS) and cost utility (Incremental £/QALY) as well as a budget impact. QALYs will be estimated from EORTC using the SCHARR algorithm (https://www.sheffield.ac.uk/scharr/sections/heds/mvh/cancer).

Costs will include that of the intervention and on any changes in use of NHS services. As the intervention cost might differ between that in the trial and in routine practice, we will take two approaches, one detailing changes in CNS team total time allocations in each arm during the trial, and another collecting data on views of both the CNS team and managers on the extent to which the intervention could be incorporated into standard practice (and under what circumstances such as being included in training and in guidelines). While both these costs will be reported, the latter would be more relevant in estimating budget impact if implemented in routine practice. The cost of changes in service use will be based on a customised resource use patient questionnaire at 6 weeks and 6 months.

Estimates of both cost and QALY increments will be estimated for each patient and incorporated into the planned statistical analyses described above. If a clinically relevant gain in the primary outcome is found to be statistically significant, more detailed economic modelling will be carried out, to explore uncertainty and to extend the time frame beyond that of the trial. This will build on the recent cost effectiveness modelling for similar interventions for hot flushes in breast cancer.[39]

All relevant resource items identified will be costed using published national cost data (British National Formulary and Personal Social Services Research Unit, and NHS reference cost). Accumulated costs and QALYs per patient will be estimated by means of area under the curve. Where appropriate we will estimate incremental cost-effectiveness ratios (ICERs). We will estimate mean values and 95% percentiles using non-parametric bootstrapping. We will produce cost-effectiveness acceptability curves (CEACs) to illustrate the uncertainty of such estimates. Major assumptions made in the costing and QALYs will be tested by means of sensitivity analyses.

7.5 QUALITATIVE ANALYSIS OF PROCESS EVALUATION

Interviews by video conferencing software or telephone will be conducted with participants and key stakeholders from each centre at completion of the intervention, to determine acceptability and factors that might influence routine practice adoption. Interview recordings will be transcribed and identifying information will be anonymised.

A two-stage approach to analysis will take place in parallel from completion of the first interview. Initially, transcripts will be coded using inductive thematic analysis using the constant comparative method, and subsequently, map emergent themes onto the Normalisation Process Theory (NPT) framework. This will be done to test the robustness of the NPT constructs against emergent themes and to facilitate a clear and thorough data-driven analysis. Analysis will be an iterative process between coding, emergent themes and NPT mapping and will involve interrogating the data for disconfirming evidence that does not fit inside the NPT framework, increasing the rigour and validity of the analysis process.
Members of the research team will hold coding and analysis meetings to discuss coding strategy, proposed themes and NPT mapping and subsequently tested in the data.

The NVivo 10 software program will be used to facilitate data storage, categorisation and retrieval.

For further information please refer to the MANCAN2 Process evaluation Manual.

8 ETHICAL CONSIDERATIONS

The trial will be conducted in accordance with the recommendations for physicians involved in research on human participants adopted by the 18th World Medical Assembly, Helsinki 1964 as revised and recognised by governing laws and EU Directives. Each participant's consent to participate in the trial should be obtained after a full explanation has been given of treatment options, including the conventional and generally accepted methods of treatment. The right of the participant to refuse to participate in the trial without giving reasons must be respected.

After the participant has entered the study, the responsible investigator may give alternative treatment to that specified in the protocol, at any stage, if they feel it to be in the best interest of the participant. However, reasons for doing so should be recorded and the participant will remain within the trial for the purpose of follow-up and data analysis according to the treatment option to which they have been allocated. Similarly, the participant remains free to withdraw at any time from protocol treatment and trial follow-up without giving reasons and without prejudicing their further treatment.

8.1 ETHICAL APPROVAL

The study protocol has been submitted to the Health Research Authority and received the favourable opinion of a Research Ethics Committee.

8.2 INFORMED CONSENT PROCESS

Informed consent is a process that is initiated prior to an individual agreeing to participate in a trial and continues throughout the individual's participation. In obtaining and documenting informed consent, the investigator should comply with applicable regulatory requirements and should adhere to the principles of GCP.

Discussion of objectives, risks and inconveniences of the trial and the conditions under which it is to be conducted are to be provided to the participant by appropriately delegated staff with knowledge in obtaining informed consent with reference to the participant information sheet (PIS). This information will emphasise that participation in the trial is voluntary and that the participant may withdraw from the trial at any time and for any reason. The participant will be given the opportunity to ask any questions that may arise and provided the opportunity to discuss the trial with family members, friends or an independent healthcare professional outside of the research team and time to consider the information prior to agreeing to participate.

8.3 CONFIDENTIALITY

SCTU will preserve the confidentiality of participants taking part in the study. The investigator must ensure that participant's anonymity will be maintained and that their identities are protected from unauthorised parties. On CRFs participants will not be identified by their names, but by an identification code.

Personal information (such as postal address; telephone number and email address) will be provided by patients on the contacts details form. Patients will consent for these details to be sent securely (via nhs.net email or UoS safesend) from the study research team at site to the research team at Southampton Clinical Trials Unit (SCTU). The contact forms will be stored securely in locked cabinets at both the site and SCTU. The contact forms will be stored separately from pseudonymised data (such as study data collection forms and patient case report forms).
The participant (patient; CNS team member; Medic and Manager) interview recordings will be transcribed and pseudonymised. Any names or details that could identify the participants or any other individuals will be removed. Quotes used from the interviews will by anonymous. Contact details containing personal

identifiable data will be stored in a locked filing cabinet at SCTU, in a separated location from interview information. The contact details may be stored electronically at the SCTU on secure UoS computer systems. The files will be password protected.

9 SPONSOR

University Hospital Southampton NHS Foundation Trust is the research sponsor for this study. SCTU, the CI and other appropriate organisations have been delegated specific duties by the Sponsor and this is documented in the trial task allocation matrix. The duties assigned to the trial sites (NHS Trusts or others taking part in this study) are detailed in the Non-Commercial Agreement.

9.1 INDEMNITY

UHS will be sponsor and will provide indemnity and compensation in the event of a claim for negligent harm, on or on behalf of a participant according to HSG (96) 48 reference no.2. If there is negligent harm during the clinical trial when the NHS body owes a duty of care to the person harmed, NHS Indemnity covers NHS staff, medical academic staff with honorary contracts, and those conducting the study. NHS Indemnity does not offer no-fault compensation and is unable to agree in advance to pay compensation for non-negligent harm. Ex-gratia payments may be considered in the case of a claim.

9.2 FUNDING

The trial is funded by a research grant from the NIHR Research for Patient Benefit (RfPB) programme.

9.2.1 Site payments

The payments assigned to the trial sites (NHS Trusts or others taking part in this study) are detailed in the Non-Commercial Agreement. This trial is part of the NIHR portfolio. This enables Trusts to apply to their Clinical Research Network for service support costs, if required.

9.2.2 Participant payments

Participants participating in the Qualitative Interviews will receive £10 gift vouchers.

9.3 AUDITS AND INSPECTIONS

The trial may be subject to inspection and audit by UHS (under their remit as Sponsor), SCTU (as the Sponsor's delegate) and other regulatory bodies to ensure adherence to the principles of GCP, UK Policy Framework for Health & Social Care Research, applicable contracts/agreements and national regulations.

10 TRIAL OVERSIGHT GROUPS

The day-to-day management of the trial will be co-ordinated through the SCTU and oversight will be maintained by the TMG and TSC.

10.1 TRIAL MANAGEMENT GROUP (TMG)

The TMG is responsible for overseeing progress of the study, including both the clinical and practical aspects. The Chair of the TMG will be the CI of the study.

The MANCAN2 TMG charter defines the membership, terms of reference, roles, responsibilities, authority, decision-making and relationships of the TMG, including the timing of meetings, frequency and format of meetings and relationships with other trial committees.

10.2 TRIAL STEERING COMMITTEE (TSC)

The TSC act as the oversight body on behalf of the Sponsor and Funder. The TSC will meet at least twice a year. The majority of members of the TSC, including the Chair, should be independent of the trial.

The MANCAN2 TSC charter defines the membership, terms of reference, roles, responsibilities, authority, decision-making and relationships of the TSC, including the timing of meetings, frequency and format of meetings and relationships with other trial committees.

10.3 DATA MONITORING AND ETHICS COMMITTEE (DMEC)

No DMEC will be convened for MANCAN2. This role will be assumed by the TSC.

11 DATA MANAGEMENT

Paper case report forms (CRFs) will be used as source documentation. Patient baseline and clinical data will be collected by two methods: the Baseline Questionnaires (including Demographic data and study Questionnaires) will be posted out to the patient and they will complete and return to SCTU using the Freepost envelope provided. Baseline Forms (capturing clinical data) will be completed by the site research team, using data available in the patient's medical notes. The site research team will call the patient to collect some of the data too.

Participant data collected on the Baseline Form will subsequently be entered onto Medidata RAVE EDC by the research team at site. Study CRFs that are returned to SCTU will be entered onto Medidata RAVE EDC by the research team at SCTU.

Study questionnaires that have been completed with the patient over the telephone will be transcribed by the SCTU research team onto paper CRFs and subsequently entered onto Medidata RAVE EDC.

All CRFs will be stored in a secure locked filing cabinet at SCTU. Only the research team will have access to these. Participant contact details forms will be stored in a secure locked filing cabinet at SCTU, separately from the study data questionnaire/ CRFs. The research team at study sites will store patient contact details forms and CRFs that they complete in a secure locked filing cabinet at site. Contact details will be stored separately to the CRFs.

The NoMAD questionnaire and the Patient Evaluation Questionnaire will be completed by the patient electronically. The research team at SCTU will send the NoMAD questionnaires to participants via a secure email (nhs.net or UoS safesend). Participants will complete electronically and will return to the research team at SCTU using a secure email (nhs.net or UoS safesend). The questionnaires will be filed on the secure University of Southampton computer systems and will be password protected. Only the research team will have access and the questionnaires will be pseudonymised. Copies will be printed and stored in a secure, locked cabinet in SCTU. The CRFs will be stored separately from participant identifiable information (such as contact details forms).

The research team at site will email the Patient Evaluation Questionnaire to the participants once they have completed their virtual group post-intervention workshop. Participants will complete electronically and will return to the research team at site using a secure email (nhs.net or UoS safesend). The questionnaires will be filed on the secure computer systems at site and will be password protected. The site research team will send a copy of the password protected pseudonymised questionnaire to the research team at SCTU using a secure email (nhs.net or safesend). Copies will be printed and stored in a secure, locked cabinet in SCTU. The CRFs will be stored separately from participant identifiable information (such as contact details forms). Participants may complete via post/ over the telephone if preferred.

The participant information sheet (PIS) and informed consent form (ICF) will outline the participant data to be collected and how it will be managed or might be shared; including handling of all Patient Identifiable Data (PID) and sensitive PID adhering to relevant data protection law.

The PI is responsible for ensuring the accuracy, completeness, and timeliness of the data entered at site. The participant data is pseudonymised by assigning each participant a participant identifier code which is used to identify the participant during the trial and for any participant specific clarification between SCTU and site. The site retains a participant identification code list which is only available to site staff. Trained personnel with specific roles assigned will be granted access to the electronic case report forms (eCRFs). The eCRF

completion guidelines will be provided to the investigator sites to aid data entry of participant information. Only the Investigator and personnel authorised by them should enter or change data in the eCRFs.

A Data Management Plan (DMP) providing full details of the trial specific data management strategy for the trial will be available and a Trial Schedule with planned and actual milestones, CRF tracking and central monitoring for active trial management created. Data queries will either be automatically generated within the eCRF, or manually raised by the trial team, if required. All alterations made to the eCRF will be visible via an audit trail which provides the identity of the person who made the change, plus the date and time. At the end of the trial after all queries have been resolved and the database frozen, the PI will confirm the data integrity by electronically signing all the eCRFs. The eCRFs and paper CRFs will be archived according to SCTU policy and a PDF copy including all clinical and Meta data returned to the PI for each participant. Data may be requested from the Data Access Committee at SCTU. Any request will be considered on a monthly basis.

SAEs and end of study information will be recorded on paper CRFs and the DMP for data entry onto RAVE will be adhered to.

The Southampton Clinical Trials Unit will keep non-identifiable data for 25 years after the study has finished.

The audio files of the recorded interviews will initially be saved on Microsoft Stream via Microsoft Teams and moved to a password-protected secure network. An audio recording of the interview will be sent to a University approved transcription company who will securely manage the audio recording and return a transcribed word document via email, where a copy will be stored on the secure network for analysis. The interview recordings will be destroyed at the end of the study. The anonymised transcripts will be stored on a password protected file on secure University of Southampton computers for up to 3 years.

The audio files of the recorded workshops will be sent securely to the research team at SCTU via an nhs.net email account or Dropoff- a UoS secure file transfer system. They will be stored securely on the computer system at the University of Southampton, which only the research team can access. Depending on the video conferencing software used at site, video files of the recorded workshop may be obtained too and sent to SCTU using the same secure channel. The workshop recordings will be destroyed at the end of the study.

Appropriate review or regulatory bodies that wish to verify the integrity of the data will be given access to the anonymous transcripts. All laptops will be password protected, and no identifiable data will be stored locally on laptops except where required to move files. Analysis will only be by pre-defined members of the research team. The data will not be exported outside the UK.

11.1 DATA SHARING REQUESTS FOR RESULTS THAT ARE AVAILABLE IN THE PUBLIC DOMAIN

In order to meet our ethical obligation to responsibly share data generated by interventional clinical trials, SCTU operate a transparent data sharing request process. As a minimum, anonymous data will be available for request from three months after publication of an article, to researchers who provide a completed Data Sharing request form that describes a methodologically sound proposal, for the purpose of the approved proposal and if appropriate a signed Data Sharing Agreement. Data will be shared once all parties have signed relevant data sharing documentation.

Researchers interested in our data are asked to complete the Request for Data Sharing form (CTU/FORM/5219) [template located on the SCTU web site, www.southampton.ac.uk/ctu] to provide a brief research proposal on how they wish to use the data. It will include; the objectives, what data are requested, timelines for use, intellectual property and publication rights, data release definition in the contract and participant informed consent etc. If considered necessary, a Data Sharing Agreement from Sponsor may be required.

12 MONITORING

12.1 CENTRAL MONITORING

Data stored at SCTU will be checked for missing or unusual values (range checks) and checked for consistency within participants over time. Any suspect data will be returned to the site in the form of data queries. Data query forms will be produced at SCTU from the trial database and sent either electronically or through the mail to a named individual (as listed on the site delegation log). Sites will respond the queries providing an explanation/resolution to the discrepancies and return the data query forms to SCTU. The forms will then be filed along with the appropriate CRFs and the appropriate corrections made on the database. There are a number of monitoring features in place at SCTU to ensure reliability and validity of the trial data, which are detailed in the trial monitoring plan.

12.2 CLINICAL SITE MONITORING

Given the nature of the trial, clinical site monitoring is not expected.

13 RECORD RETENTION AND ARCHIVING

Trial documents will be retained in a secure location during and after the trial has finished.

The PI or delegate must maintain adequate and accurate records to enable the conduct of the trial to be fully documented and the trial data to be subsequently verified. After trial closure the PI will maintain all source documents and trial related documents. All source documents will be retained for a period of 25 years following the end of the trial.

Sites are responsible for archiving the ISF and participants' medical records.

The Sponsor is responsible for archiving the TMF and other relevant trial documentation.

14 PUBLICATION POLICY

Data from all centres will be analysed together and published as soon as possible. Individual investigators may not publish data concerning their patients that are directly relevant to questions posed by the trial until the TMG has published its report. The TMG will form the basis of the Writing Committee and advise on the nature of publications. All publications shall include a list of investigators, and if there are named authors, these should include the Chief Investigator, Co-Investigators, Trial Manager, and Statistician(s) involved in the trial. Named authors will be agreed by the CI and Director of SCTU. If there are no named authors, then a 'writing committee' will be identified.

15 APPENDICES

15.1 SWAT PROTOCOL

Using a theoretically informed cover letter to improve response rates to 6- month postal questionnaires.

Objective of this SWAT

To improve response rates to 6- month postal questionnaires which provide information on patient reported outcomes in a randomised trial.

Study area: Follow-up, Retention

Sample type: Participants

Estimated funding level needed: Unfunded

Background Retention has been identified as a top methodological priority for trial methodology research within the UK.[40] Patient attrition has been an ongoing and persistent issue across trials with a recent Cochrane review of the literature reporting no high-quality evidence to inform interventions suited to tackle this issue.[41] It is anticipated that the longer the follow-up period, the larger the patient attrition rate, and consequently loss of outcome data.[42]

Paper questionnaires remain a common method used to collect data remotely due to patient preference. To establish evidence-based methods to improve conduct in the area or questionnaire return rate the MANCAN2 trial aims to replicate this SWAT as part of the Trial Forge initiative to improve trial efficacy.[43] An individual SWAT needs replicating several times to contribute to a meta-analysis and at present this SWAT is not powered sufficiently to provide a definitive answer to the question 'does using a theoretically informed cover letter to improve response rates to annual postal questionnaires?'.

Return of a postal questionnaire can be considered a behaviour and therefore a novel behaviour change intervention was developed to target the desired behaviour; participants returning a questionnaire.

Method

The behaviour change intervention was developed using the Theoretical Domains Framework (TDF) as a tool for identifying theoretical targets for behaviour change interventions.[45] Behaviour change techniques targeting the identified domains were operationalised in the cover letter accompanying the postal questionnaires. Participants due the 6-month postal questionnaire will be randomised to receive either the theoretically informed cover letter or standard cover letter with their postal questionnaire. Response rates and time to response will be compared amongst groups.

Intervention 1: Theoretically informed cover letter issued with 6-month questionnaires

Intervention 2 (Control): Standard cover letter issued with 6-month questionnaires

Index Type: Method of Follow-up

Method for allocating to intervention or comparator.

Randomisation for theory informed cover letter vs standard cover letter

Outcome measures

Primary Outcome: Response rate to 6-month questionnaires.

Analysis plans

Primary analysis will be a comparison of the number of questionnaires issued and returned between the intervention groups.

Further analysis will compare the date the questionnaire was sent vs the date the questionnaire was completed between the intervention groups.

16 REFERENCES

[1] Smith-Palmer J, Takizawa C, Valentine W. Literature review of the burden of prostate cancer in Germany, France, the United Kingdom and Canada. BMC Urol. 2019;19:19.
[2] Merriel SWD, May MT, Martin RM. Predicting prostate cancer progression: protocol for a retrospective cohort study to identify prognostic factors for prostate cancer outcomes using routine primary care data. BMJ Open. 2018;8:e019409.
[3] Davis ID, Martin AJ, Stockler MR, Begbie S, Chi KN, Chowdhury S, et al. Enzalutamide with Standard First-Line Therapy in Metastatic Prostate Cancer. N Engl J Med. 2019;381:121-31.
[4] James ND, Spears MR, Clarke NW, Dearnaley DP, De Bono JS, Gale J, et al. Survival with Newly Diagnosed Metastatic Prostate Cancer in the "Docetaxel Era": Data from 917 Patients in the Control Arm of the STAMPEDE Trial (MRC PR08, CRUK/06/019). Eur Urol. 2015;67:1028-38.
[5] Gunner C, Gulamhusein A, Rosario DJ. The modern role of androgen deprivation therapy in the management of localised and locally advanced prostate cancer. J Clin Urol. 2016;9:24-9.
[6] Vitolins MZ, Griffin L, Tomlinson WV, Vuky J, Adams PT, Moose D, et al. Randomized trial to assess the impact of venlafaxine and soy protein on hot flashes and quality of life in men with prostate cancer. J Clin Oncol. 2013;31:4092-8.
[7] Frisk J. Managing hot flushes in men after prostate cancer--a systematic review. Maturitas. 2010;65:15-22.
[8] Siddiqui ZA, Krauss DJ. Adjuvant androgen deprivation therapy for prostate cancer treated with radiation therapy. Transl Androl Urol. 2018;7:378-89.
[9] Crawford ED, Heidenreich A, Lawrentschuk N, Tombal B, Pompeo ACL, Mendoza-Valdes A, et al. Androgen-targeted therapy in men with prostate cancer: evolving practice and future considerations. Prostate Cancer Prostatic Dis. 2019;22:24-38.
[10] Jones JM, Kohli M, Loprinzi CL. Androgen deprivation therapy-associated vasomotor symptoms. Asian J Androl. 2012;14:193-7.
[11] Yousaf O, Stefanopoulou E, Grunfeld EA, Hunter MS. A randomised controlled trial of a cognitive behavioural intervention for men who have hot flushes following prostate cancer treatment (MANCAN): trial protocol. BMC Cancer. 2012;12:230.
[12] Adelson KB, Loprinzi CL, Hershman DL. Treatment of hot flushes in breast and prostate cancer. Expert Opin Pharmacother. 2005;6:1095-106.
[13] Fenlon D, Maishman T, Day L, Nuttall J, May C, Ellis M, et al. Effectiveness of nurse-led group CBT for hot flushes and night sweats in women with breast cancer: Results of the MENOS4 randomised controlled trial. Psychooncology. 2020;29:1514-23.
[14] Stefanopoulou E, Yousaf O, Grunfeld EA, Hunter MS. A randomised controlled trial of a brief cognitive behavioural intervention for men who have hot flushes following prostate cancer treatment (MANCAN). Psychooncology. 2015;24:1159-66.
[15] Qan'ir Y, DeDeaux D, Godley PA, Mayer DK, Song L. Management of Androgen Deprivation Therapy-Associated Hot Flashes in Men With Prostate Cancer. Oncol Nurs Forum. 2019;46:E107-E18.
[16] Irani J, Salomon L, Oba R, Bouchard P, Mottet N. Efficacy of venlafaxine, medroxyprogesterone acetate, and cyproterone acetate for the treatment of vasomotor hot flushes in men taking gonadotropin-releasing hormone analogues for prostate cancer: a double-blind, randomised trial. Lancet Oncol. 2010;11:147-54.
[17] Hunter MS, Mann E. A cognitive model of menopausal hot flushes and night sweats. J Psychosom Res. 2010;69:491-501.
[18] Swartzman LC, Edelberg R, Kemmann E. Impact of stress on objectively recorded menopausal hot flushes and on flush report bias. Health Psychol. 1990;9:529-45.
[19] Freedman RR. Pathophysiology and treatment of menopausal hot flashes. Semin Reprod Med. 2005;23:117-25.
[20] Rendall MJ, Simonds LM, Hunter MS. The Hot Flush Beliefs Scale: a tool for assessing thoughts and beliefs associated with the experience of menopausal hot flushes and night sweats. Maturitas. 2008;60:158-69.

[21] Ayers B, Hunter MS. Health-related quality of life of women with menopausal hot flushes and night sweats. Climacteric. 2013;16:235-9.
[22] Duijts SF, van Beurden M, Oldenburg HS, Hunter MS, Kieffer JM, Stuiver MM, et al. Efficacy of cognitive behavioral therapy and physical exercise in alleviating treatment-induced menopausal symptoms in patients with breast cancer: results of a randomized, controlled, multicenter trial. J Clin Oncol. 2012;30:4124-33.
[23] Mann E, Smith MJ, Hellier J, Balabanovic JA, Hamed H, Grunfeld EA, et al. Cognitive behavioural treatment for women who have menopausal symptoms after breast cancer treatment (MENOS 1): a randomised controlled trial. Lancet Oncol. 2012;13:309-18.
[24] Stefanopoulou E, Hunter MS. Telephone-guided Self-Help Cognitive Behavioural Therapy for menopausal symptoms. Maturitas. 2014;77:73-7.
[25] Atema V, van Leeuwen M, Kieffer JM, Oldenburg HSA, van Beurden M, Hunter MS, et al. Internet-based cognitive behavioral therapy aimed at alleviating treatment-induced menopausal symptoms in breast cancer survivors: Moderators and mediators of treatment effects. Maturitas. 2020;131:8-13.
[26] Eziefula CU, Grunfeld EA, Hunter MS. 'You know I've joined your club... I'm the hot flush boy': a qualitative exploration of hot flushes and night sweats in men undergoing androgen deprivation therapy for prostate cancer. Psychooncology. 2013;22:2823-30.
[27] Grunfeld EA, Hunter MS, Yousaf O. Men's experience of a guided self-help intervention for hot flushes associated with prostate cancer treatment. Psychol Health Med. 2017;22:425-33.
[28] Hunter MS, Ayers B, Smith M. The Hot Flush Behavior Scale: a measure of behavioral reactions to menopausal hot flushes and night sweats. Menopause. 2011;18:1178-83.
[29] Frankland J, Brodie H, Cooke D, Foster C, Foster R, Gage H, et al. Follow-up care after treatment for prostate cancer: evaluation of a supported self-management and remote surveillance programme. BMC Cancer. 2019;19:368.
[30] Rand KL, Otte JL, Flockhart D, Hayes D, Storniolo AM, Stearns V, et al. Modeling hot flushes and quality of life in breast cancer survivors. Climacteric. 2011;14:171-80.
[31] Hunter MS, Liao KL. A psychological analysis of menopausal hot flushes. Br J Clin Psychol. 1995;34:589-99.
[33] Spitzer RL, Kroenke K, Williams JB, Lowe B. A brief measure for assessing generalized anxiety disorder: the GAD-7. Arch Intern Med. 2006;166:1092-7.
[34] Kroenke K, Spitzer RL, Williams JB. The PHQ-9: validity of a brief depression severity measure. J Gen Intern Med. 2001;16:606-13.
[35] Buysse DJ, Reynolds CF, 3rd, Monk TH, Berman SR, Kupfer DJ. The Pittsburgh Sleep Quality Index: a new instrument for psychiatric practice and research. Psychiatry Res. 1989;28:193-213.
[36] Mundt JC, Marks IM, Shear MK, Greist JH. The Work and Social Adjustment Scale: a simple measure of impairment in functioning. Br J Psychiatry. 2002;180:461-4.
[37] Hunter M, Smith M. Managing Hot Flushes with Group Cognitive Behaviour Therapy: An Evidence Based Treatment Manual for Health Professionals: Routledge; 2014.
[38] Beecham J, Knapp M. Costing psychiatric interventions. In: Thornicroft G, Brewin C, Wing J, editors. Measuring mental health needs: Gaskell Royal College of Psychiatrists; 1992.
[39] Verbeek JGE, Atema V, Mewes JC, van Leeuwen M, Oldenburg HSA, van Beurden M, et al. Cost-utility, cost-effectiveness, and budget impact of Internet-based cognitive behavioral therapy for breast cancer survivors with treatment-induced menopausal symptoms. Breast Cancer Res Treat. 2019;178:573-85.
[40] Tudur Smith C, Hickey H, Clarke M, Blazeby J, Williamson P. The trials methodological research agenda: results from a priority setting exercise. Trials. 2014;15:32.
[41] Gillies K, Kearney A, Keenan C, Treweek S, Hudson J, Brueton VC, et al. Strategies to improve retention in randomised trials. Cochrane Database Syst Rev. 2021;3:MR000032.
[42] Walters SJ, Bonacho Dos Anjos Henriques-Cadby I, Bortolami O, Flight L, Hind D, Jacques RM, et al. Recruitment and retention of participants in randomised controlled trials: a review of trials funded and published by the United Kingdom Health Technology Assessment Programme. BMJ Open. 2017;7:e015276.
[43] Treweek S, Altman DG, Bower P, Campbell M, Chalmers I, Cotton S, et al. Making randomised trials more efficient: report of the first meeting to discuss the Trial Forge platform. Trials. 2015;16:261.

[44] Horevoorts NJ, Vissers PA, Mols F, Thong MS, van de Poll-Franse LV. Response rates for patient-reported outcomes using web-based versus paper questionnaires: comparison of two invitational methods in older colorectal cancer patients. J Med Internet Res. 2015;17:e111.
[45] Michie S, Johnston M, Abraham C, Lawton R, Parker D, Walker A, et al. Making psychological theory useful for implementing evidence based practice: a consensus approach. Qual Saf Health Care. 2005;14:26-33.

1. SUMMARY OF SIGNIFICANT CHANGES TO THE PROTOCOL

Protocol date and version	Summary of significant changes
**V1 02 Oct 2021**	**First Protocol**
**V2 25 Feb 2022**	**Section 4.2, Inclusion Criterion 7- addition of face-to-face workshops.**<br><br>**Section 5.2 Trial Procedures- addition of wording to allow for sites to deliver the group workshops face-to-face or hybrid (a mix of face-to-face or virtual).** (Mixed meetings should not be attempted. Thus, participants should either all be face to face, or all virtual, for any particular meeting)<br>**The method of delivery will be determined by the local Principal Investigator, based on their assessment of what would be optimal.**
**V3 27 Jul 2022**	**Trial Synopsis and sections 4.2 and 4.3, Eligibility Criteria (Inclusion/ Exclusion)**<br><br>**A relaxation of the eligibility criteria. Specifically, changes to Inclusion Criteria Items 2 and 3, changes to Exclusion criteria items 1, 3, 4 and removal of exclusion criteria 2.**<br><br>**Table 1, Schedule of Observations and Procedures**<br><br>**Removal of 'up to 2 months' window for the screening procedures.**<br><br>**Change of wording for the CNS Training CRF/ Assessment Questionnaire column. Specifically, changed wording from 'CNS Logs' to a more accurate description 'CNS Training Evaluation Questionnaire'.**<br><br>**University of Southampton logo updated on page 1.**<br><br>**Trial Coordinating Centre Fax number removed from page 2. Faxing is no longer a route of communication at SCTU.**
**V4 23 Feb 2023**	**Removal of reference to the Quality-of-Life Measure EORTC QLQ-PR25 from sections 5.3.1.2; 5.3.3; Table 1 (Schedule of Observations and Procedures) and the reference section.**<br><br>**Removal of 'virtual' in the study title, and addition of 'guided' to reflect the changes submitted in Amendment 2 (the option for sites**

	**to deliver the workshops face to face, virtually or hybrid, rather than just virtually).**<br><br>**The addition of the ISRCTN reference number to the front covering page.**
