# Supplementary material for: A multicentre randomised controlled trial of a guided self-help cognitive behavioural therapy to MANage the impact of hot flushes and night sweats in patients with prostate CANcer undergoing androgen deprivation therapy (MANCAN2)
Source: Trials. 2023 Jul 10;24:450. doi: 10.1186/s13063-023-07325-w (PMC10332063; doi:10.1186/s13063-023-07325-w)
Supplement: Supplementary file 2 — Additional file 2. REC Favourable Opinion. [file 13063_2023_7325_MOESM2_ESM.pdf]

Health Research Authority

West Midlands - South Birmingham Research Ethics Committee
Equinox House
City Link
Nottingham
NG2 4LA

Please note: This is the favourable opinion of the REC only and does not allow you to start your study at NHS sites in England until you receive HRA Approval

16 December 2021

Dr Simon Crabb
Associate Professor in Medical Oncology
University of Southampton
Southampton Clinical Trials Unit
Cancer Sciences Building
Mailpoint 824
SO16 6YD

Dear Dr Crabb

**Study title:**	**A multicentre randomised controlled trial of virtual self-help cognitive behavioural therapy to MANage the impact of hot flush and night sweat symptoms in patients with prostate CANcer undergoing androgen deprivation therapy (MANCAN2).**
**REC reference:**	**21/WM/0259**
**Protocol number:**	**RHM CAN1636**
**IRAS project ID:**	**304500**

Thank you for responding to the Research Ethics Committee's (REC) request for further information on the above research and submitting revised documentation.

Confirmation of ethical opinion

On behalf of the Committee, I am pleased to confirm a favourable ethical opinion for the above research on the basis described in the application form, protocol and supporting documentation as revised, subject to the conditions specified below.

Good practice principles and responsibilities

The UK Policy Framework for Health and Social Care Research sets out principles of good practice in the management and conduct of health and social care research. It also outlines the responsibilities of individuals and organisations, including those related to the four elements of research transparency:

1. registering research studies
2. reporting results
3. informing participants
4. sharing study data and tissue

Conditions of the favourable opinion

The REC favourable opinion is subject to the following conditions being met prior to the start of the study.

Confirmation of Capacity and Capability (in England, Northern Ireland and Wales) or NHS management permission (in Scotland) should be sought from all NHS organisations involved in the study in accordance with NHS research governance arrangements. Each NHS organisation must confirm through the signing of agreements and/or other documents that it has given permission for the research to proceed (except where explicitly specified otherwise).

Guidance on applying for HRA and HCRW Approval (England and Wales)/ NHS permission for research is available in the Integrated Research Application System.

For non-NHS sites, site management permission should be obtained in accordance with the procedures of the relevant host organisation.

Sponsors are not required to notify the Committee of management permissions from host organisations

Registration of Clinical Trials

All research should be registered in a publicly accessible database and we expect all researchers, research sponsors and others to meet this fundamental best practice standard.

It is a condition of the REC favourable opinion that **all clinical trials are registered** on a publicly accessible database within six weeks of recruiting the first research participant. For this purpose, 'clinical trials' are defined as the first four project categories in IRAS project filter question 2. Failure to register a clinical trial is a breach of these approval conditions, unless a deferral has been agreed by or on behalf of the Research Ethics Committee (see here for more information on requesting a deferral:
https://www.hra.nhs.uk/planning-and-improving-research/research-planning/research-registration-research-project-identifiers/

If you have not already included registration details in your IRAS application form, you should notify the REC of the registration details as soon as possible.

Further guidance on registration is available at:
https://www.hra.nhs.uk/planning-and-improving-research/research-planning/transparency-responsibilities/

Publication of Your Research Summary

We will publish your research summary for the above study on the research summaries section of our website, together with your contact details, no earlier than three months from the date of this favourable opinion letter.

Should you wish to provide a substitute contact point, make a request to defer, or require further information, please visit:
https://www.hra.nhs.uk/planning-and-improving-research/application-summaries/research-summaries/

N.B. If your study is related to COVID-19 we will aim to publish your research summary within 3 days rather than three months.

During this public health emergency, it is vital that everyone can promptly identify all relevant research related to COVID-19 that is taking place globally. If you haven't already done so, please register your study on a public registry as soon as possible and provide the REC with the registration detail, which will be posted alongside other information relating to your project. We are also asking sponsors not to request deferral of publication of research summary for any projects relating to COVID-19. In addition, to facilitate finding and extracting studies related to COVID-19 from public databases, please enter the WHO official acronym for the coronavirus disease (COVID-19) in the full title of your study. Approved COVID-19 studies can be found at:
https://www.hra.nhs.uk/covid-19-research/approved-covid-19-research/

It is the responsibility of the sponsor to ensure that all the conditions are complied with before the start of the study or its initiation at a particular site (as applicable).

After ethical review: Reporting requirements

The attached document "After ethical review – guidance for researchers" gives detailed guidance on reporting requirements for studies with a favourable opinion, including:

- Notifying substantial amendments
- Adding new sites and investigators
- Notification of serious breaches of the protocol
- Progress and safety reports
- Notifying the end of the study, including early termination of the study
- Final report
- Reporting results

The latest guidance on these topics can be found at
https://www.hra.nhs.uk/approvals-amendments/managing-your-approval/.

Ethical review of research sites

NHS/HSC sites

The favourable opinion applies to all NHS/HSC sites taking part in the study, subject to confirmation of Capacity and Capability (in England, Northern Ireland and Wales) or management permission (in Scotland) being obtained from the NHS/HSC R&D office prior to the start of the study (see "Conditions of the favourable opinion" below).

Non-NHS/HSC sites

I am pleased to confirm that the favourable opinion applies to any non-NHS/HSC sites listed in the application, subject to site management permission being obtained prior to the start of the study at the site.

Approved documents

The final list of documents reviewed and approved by the Committee is as follows:

Document	*Version*	*Date*
Copies of materials calling attention of potential participants to the research [MANCAN2 Patient Texts & Emails]	1	01 November 2021
Covering letter on headed paper [Cover Letter for REC]		18 October 2021
GP/consultant information sheets or letters [MANCAN2 GP Letter]	1	02 October 2021
Interview schedules or topic guides for participants [MANCAN2 Topic Guide Pre Intervention (CNS)]	1	02 October 2021
Interview schedules or topic guides for participants [MANCAN2 Topic Guide Post Intervention (CNS)]	1	02 October 2021
Interview schedules or topic guides for participants [MANCAN2 Topic Guide Post Intervention (Medic)]	1	02 October 2021
Interview schedules or topic guides for participants [MANCAN2 Topic Guide Post Intervention (Manager)]	1	02 October 2021
Interview schedules or topic guides for participants [MANCAN2 Topic Guide Post Intervention (Patient)]	1	02 October 2021
IRAS Application Form [IRAS_Form_19102021]		19 October 2021
Letter from funder [MANCAN2 Letter from Funder (NIHR)]		09 July 2020
Letter from sponsor [MANCAN2 Sponsorship in Principle]		22 April 2021
Letters of invitation to participant [MANCAN2 Site Contacts Form]	1	02 October 2021
Letters of invitation to participant [MANCAN2 SWAT Cover Letter (6 Month Questionnaires)]	1	25 August 2021
Letters of invitation to participant [MANCAN2 SWAT Cover Letter CONTROL (6 Month Questionnaires)]	1	02 October 2021
Non-validated questionnaire [MANCAN2 Eligibility Checklist]	1	02 October 2021
Non-validated questionnaire [MANCAN2 Baseline Form]	1	02 October 2021
Non-validated questionnaire [MANCAN2 End of Study Form]	1	02 October 2021
Non-validated questionnaire [MANCAN2 Patient Evaluation Questionnaire]	1	02 October 2021
Non-validated questionnaire [MANCAN2 Resource Usage Questionnaire]	V1	02 October 2021
Other [Application Clarification]		03 November 2021
Other [Cover Letter (Response to Initial Submission Feedback)]		08 December 2021
Other [Patient Information Sheet]	Version 2	08 December 2021
Other [Tracked Patient Information Sheet]	Version 2	08 December 2021

	(Tracked)	
Other [Tracked Patient Informed Consent Form_Version 2]	Version 2 (Tracked)	08 December 2021
Other [Tracked Invite Pack_Version 2]	Version 2 (Tracked)	08 December 2021
Other [Invite Pack]	Version 2	08 December 2021
Other [Patient Informed Consent Form]	Version 2	08 December 2021
Other [CBT Booklet Version 2]	Version 2	08 December 2021
Other [NoMAD Pre Intervention]	Version 1	21 June 2021
Other [NoMAD Post Intervention]	Version 1	21 June 2021
Other [Baseline Questionnaires]	Version 2	08 December 2021
Other [6 week Questionnaires]	Version 2	08 December 2021
Other [6 month Questionnaires]	Version 2	08 December 2021
Other [Process Evaluation]	Version 2 (Tracked)	08 December 2021
Other [MANCAN2 CBT Booklet (Tracked Changes)]	Version 2 (Tracked)	08 December 2021
Other [MANCAN2_Process Evaluation and Health Economics Manual]	Version 2	08 December 2021
Participant consent form [MANCAN2 Informed Consent Form (CNS/Medic/Manager)]	1	02 October 2021
Participant information sheet (PIS) [MANCAN2 Particpant Information Sheet CNS Team Member]	1	02 October 2021
Participant information sheet (PIS) [MANCAN2 Participant Information Sheet Medic]	1	02 October 2021
Participant information sheet (PIS) [MANCAN2 Participant Information Sheet Manager]	1	02 October 2021
Research protocol or project proposal [MANCAN2 Protocol]	1	02 October 2021
Sample diary card/patient card [MANCAN2 Patient Trial Card]	1	01 November 2021
Summary CV for Chief Investigator (CI) [Chief Investigator Summary CV]	NA	16 September 2019
Summary, synopsis or diagram (flowchart) of protocol in non technical language [MANCAN2 Patient Flow]	1	02 October 2021
Summary, synopsis or diagram (flowchart) of protocol in non technical language [MANCAN2 Site Instructions (Patient Evaluation Questionnaire)]	1	02 October 2021
Summary, synopsis or diagram (flowchart) of protocol in non technical language [MANCAN2 NoMAD Questionnaire SCTU Instructions]	1	02 October 2021
Validated questionnaire [QLQ C30]		
Validated questionnaire [GAD-7]		
Validated questionnaire [PHQ-9]		
Validated questionnaire [PSQI]		
Validated questionnaire [WSAS]		
Validated questionnaire [HFNS Beliefs & Behaviour Scale for Men (Appendix 1)]		
Validated questionnaire [HFNS Rating Scale]		
Validated questionnaire [NoMAD Instrument]		

Statement of compliance

The Committee is constituted in accordance with the Governance Arrangements for Research Ethics Committees and complies fully with the Standard Operating Procedures for Research Ethics Committees in the UK.

User Feedback

The Health Research Authority is continually striving to provide a high quality service to all applicants and sponsors. You are invited to give your view of the service you have received and the application procedure. If you wish to make your views known please use the feedback form available on the HRA website:
http://www.hra.nhs.uk/about-the-hra/governance/quality-assurance/

HRA Learning

We are pleased to welcome researchers and research staff to our HRA Learning Events and online learning opportunities– see details at:
https://www.hra.nhs.uk/planning-and-improving-research/learning/

**IRAS project ID: 304500**	**Please quote this number on all correspondence**

With the Committee's best wishes for the success of this project.
Yours sincerely

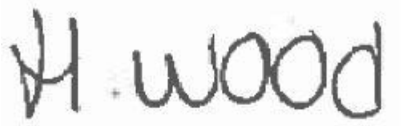

Signed on behalf of the REC
Email:southbirmingham.rec@hra.nhs.uk

Enclosures: "After ethical review – guidance for researchers"
Copy to: Miss Alannah Morgan
