# Supplementary material for: A multicentre randomised controlled trial of a guided self-help cognitive behavioural therapy to MANage the impact of hot flushes and night sweats in patients with prostate CANcer undergoing androgen deprivation therapy (MANCAN2)
Source: Trials. 2023 Jul 10;24:450. doi: 10.1186/s13063-023-07325-w (PMC10332063; doi:10.1186/s13063-023-07325-w)
Supplement: Supplementary file 3 — Additional file 3. Confirmation of NIHR Funding. [file 13063_2023_7325_MOESM3_ESM.pdf]

NIHR | National Institute for Health Research

Dr Simon Crabb
Cancer Sciences Building
Mailpoint 824
Southampton General Hospital
Southampton
SO16 6YD

CENTRAL COMMISSIONING FACILITY
Grange House
15 Church Street
Twickenham
TW1 3NL

Tel: 020 8843 8000
Fax: 020 8843 8001
Email: ccf@nihr.ac.uk
www.nihr.ac.uk/ccf

Thursday 09 July 2020

Dear Dr Crabb,

Research for Patient Benefit (RfPB) Programme: NIHR201542 – A multicentre randomised controlled trial (RCT) of a self-help cognitive behavioural therapy (CBT) intervention to reduce the impact of hot flush and night sweat (HFNS) symptoms in men with prostate cancer undergoing androgen deprivation therapy (ADT): MANaging symptoms during prostate CANcer treatment (MANCAN2)

On behalf of the National Institute for Health Research (NIHR), I am pleased to inform you that the Committee has recommended your RfPB Competition 40 Stage 2 application for funding.

<u>Committee Feedback</u>

The Committee recognised the strength of the team and the value of the proposed research. However, there are several points on which the Committee has requested the research team's response:

- The Committee commented that the proposal was highly ambitious, and sought reassurance that the project aims could be delivered within the proposed budget and timeframe. The applicants were invited to consider reducing the number of sites to facilitate the delivery of the study.
- In agreement with Reviewer 2, the eligibility criteria were considered very broad. In particular, the Committee was concerned that including prostate cancer patients at localised and advanced disease stages would make the sample too heterogeneous. The applicants were invited to provide more information about the analysis plan and clarify whether and how participants would be stratified on the basis of their treatments (*e.g.* acupuncture and hormone therapy).
- The applicants were asked to clarify how the clinical staff would be trained and supervised for delivering the intervention.
- The health economic component was still vaguely described. The applicants were asked to provide more details on how the analysis will be conducted.

- In agreement with Reviewers 1 and 2, the Committee was of the opinion that the dissemination plan was not sufficiently detailed. Specifically:
  - Provide more information on how patients and members of the public would have access to the results
  - Clarify how the intervention would be disseminated in practice if it were to be implemented in the healthcare system.

Outcomes Table

The attached outcomes table contains the anonymised mean scores and ranking from the Competition 40 Stage 2 Regional Assessment Committee meeting where your application was assessed. It is intended to aid you in contextualising your feedback from the Committee.

Your anonymised code is: **SEC-0520-2002**

Peer Review

Also attached are the external peer and public reviewers' comments relating to your application. Please note that external reviewer comments are always taken in the context of a wider discussion on a proposal and matters such as unduly harsh remarks, seeming misunderstanding or bias will have been taken into account by the Committee in coming to their decision.

I would like to take this opportunity to remind you that you should refrain from publicising this funding recommendation until a contract is in place.

If you have any questions regarding the decision of the Committee, or any matters relating to the RfPB Programme, please do not hesitate to contact me.

Yours sincerely,

Dr Laura Tornatore
Programme Manager
Email: laura.tornatore@nihr.ac.uk
Telephone: 020 8843 8148
